# Supplementary figures and images for: Functional assessment of the “two-hit” model for neurodevelopmental defects in Drosophila and X. laevis
Source: PLoS Genet. 2021 Apr 5;17(4):e1009112. doi: 10.1371/journal.pgen.1009112 (PMC8049494; doi:10.1371/journal.pgen.1009112)

**A** 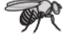 *Drosophila melanogaster*

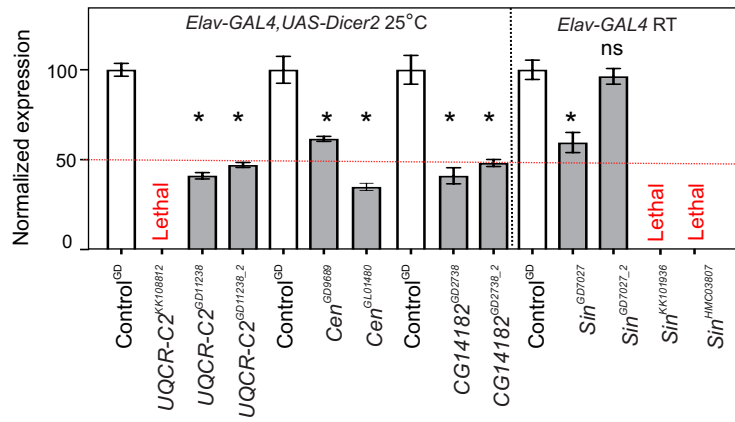

**B** 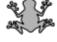 *X. laevis*

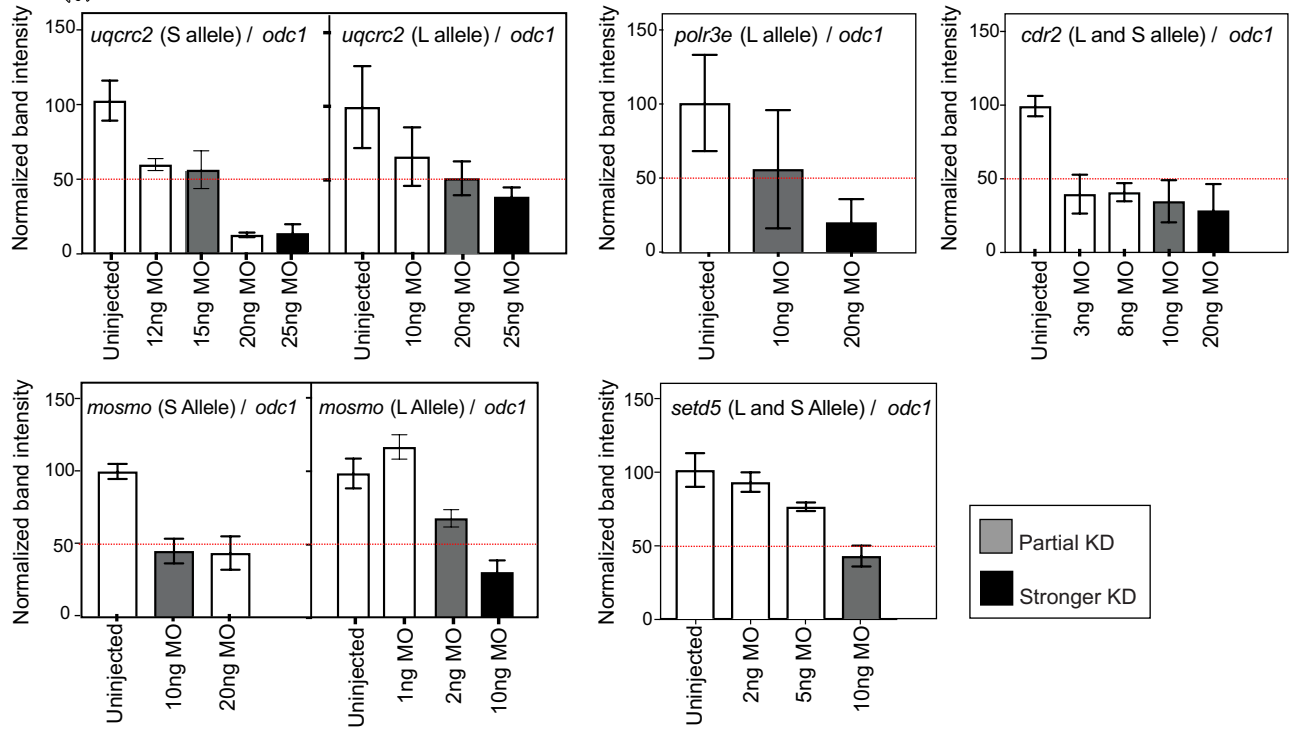

Supplement: S1 Fig — (A) Drosophila homologs of 16p12.1 genes were knocked down using nervous system-specific Elav-GAL4 driver with overexpression of Dicer2 at 25°C. RT-qPCR confirmed 40–60% knockdown of the 16p12.1 homologs (two-tailed student’s t-test, *p<0.05). As knockdown of Sin caused embryonic lethality in these conditions, all experiments in the nervous system and RT-qPCR were performed without overexpression of Dicer2 and reared at room temperature (RT). SinKK101936 and SinHMC03807 were also embryonic lethal without Dicer2. SinGD7027_2 did not show knockdown of the homolog, and the RNAi line was therefore not used for further experiments. All experiments were performed in comparison to appropriate background-specific controls. Only one control is shown per gene. GD VDRC control is shown in all cases for simplification (ControlGD). A list of full genotypes for fly crosses used in these experiments is provided in S1 File. (B) Normalized band intensity of RT-PCR of X. laevis tadpoles injected with different morpholino dosages of the 16p12.1 homologs compared to the uninjected control. Different morpholino sequences were used for the L and S alleles for uqcrc2 and mosmo, while unique sequences were used for both L and S alleles for cdr2 or setd5. As the S allele has not been annotated for polr3e, only the L allele was targeted. Colored bars represent the dosages of morpholinos used, with grey bars indicating amounts for “partial knockdown” (approximately 50% of expression) and black bars indicating amounts for “stronger knockdown”. 10 ng of morpholino was used for partial and stronger KD experiments for mosmo S allele, as increasing concentrations did not lead to differences in knockdown of the allele. Bar plots represent mean +/- SD, and red dotted lines indicate 50% expression. Statistical details are provided in S6 File. (PDF) [file pgen.1009112.s001.pdf]

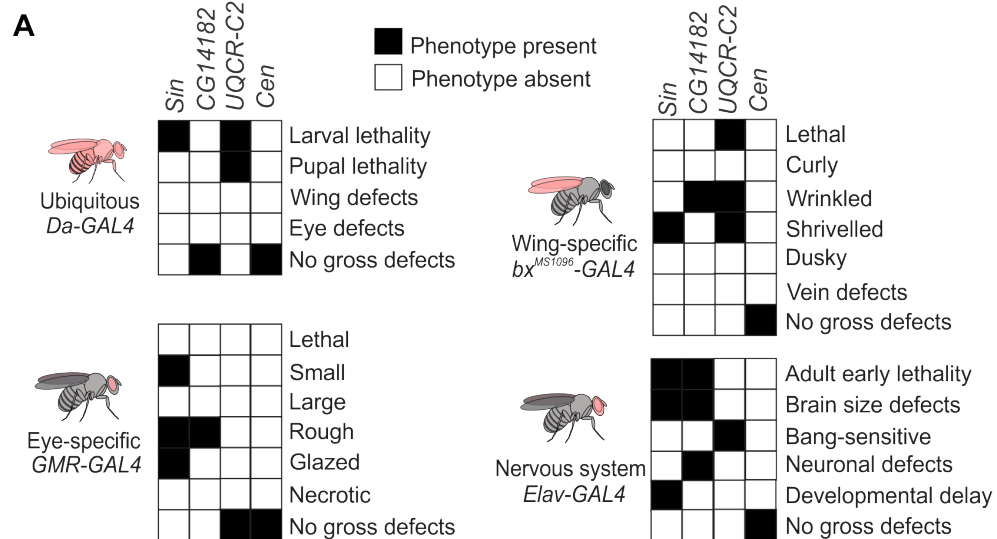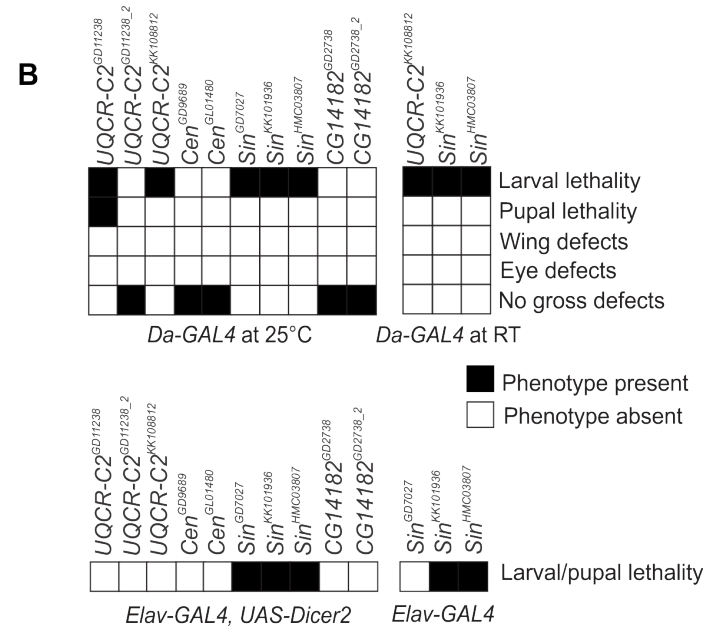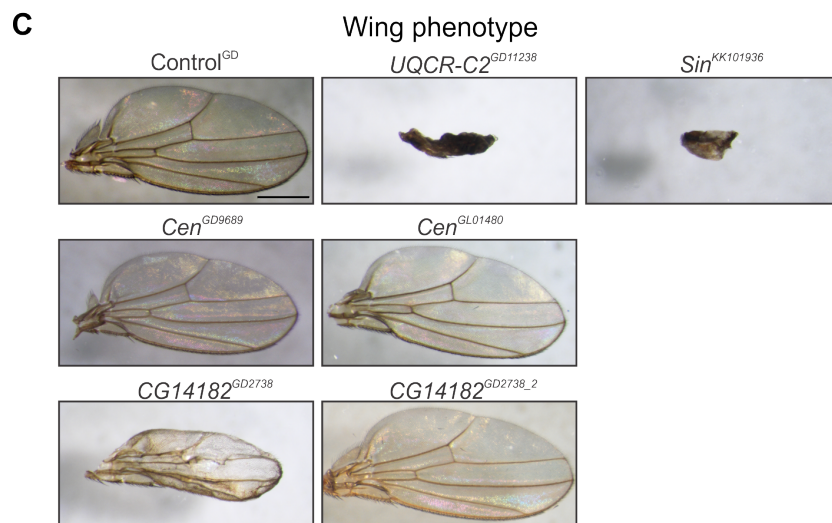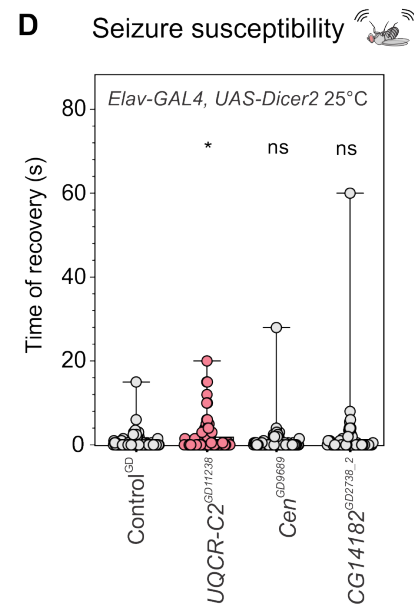

Supplement: S2 Fig — (A) Summary of phenotypes observed with tissue-specific knockdown of each of the 16p12.1 homologs. (B) Ubiquitous (Da-GAL4) and nervous system-specific (Elav-GAL4) knockdown of multiple RNAi lines for each 16p12.1 homolog showed a range of lethality and developmental defects. (C) Representative brightfield images of adult wings with knockdown of 16p12.1 homologs using the wing-specific driver bxMS1096-GAL4. Severe phenotypes were observed for UQCR-C2 and Sin, with some RNAi lines, including UQCR-C2GD11238_2, UQCR-C2KK108812 and SinGD7027, showing lethality. Scale bar represents 500μm. (D) Bang sensitivity assay for adult flies with nervous system-specific knockdown of the 16p12.1 homologs showed increased recovery time for UQCR-C2GD11238 (n = 95, two-tailed Mann-Whitney test, *p = 0.003). SinGD7027 adult flies exhibited severe motor defects and could not be tested for the phenotype. Boxplots represent all data points with median, 25th and 75th percentiles. Statistical details are provided in S6 File. A list of full genotypes for fly crosses used in these experiments is provided in S1 File. (PDF) [file pgen.1009112.s002.pdf]

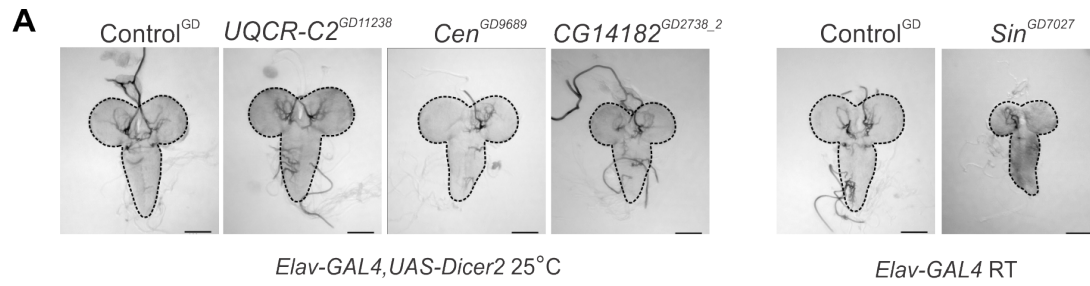

**B** Cellular processes in brain development 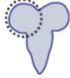

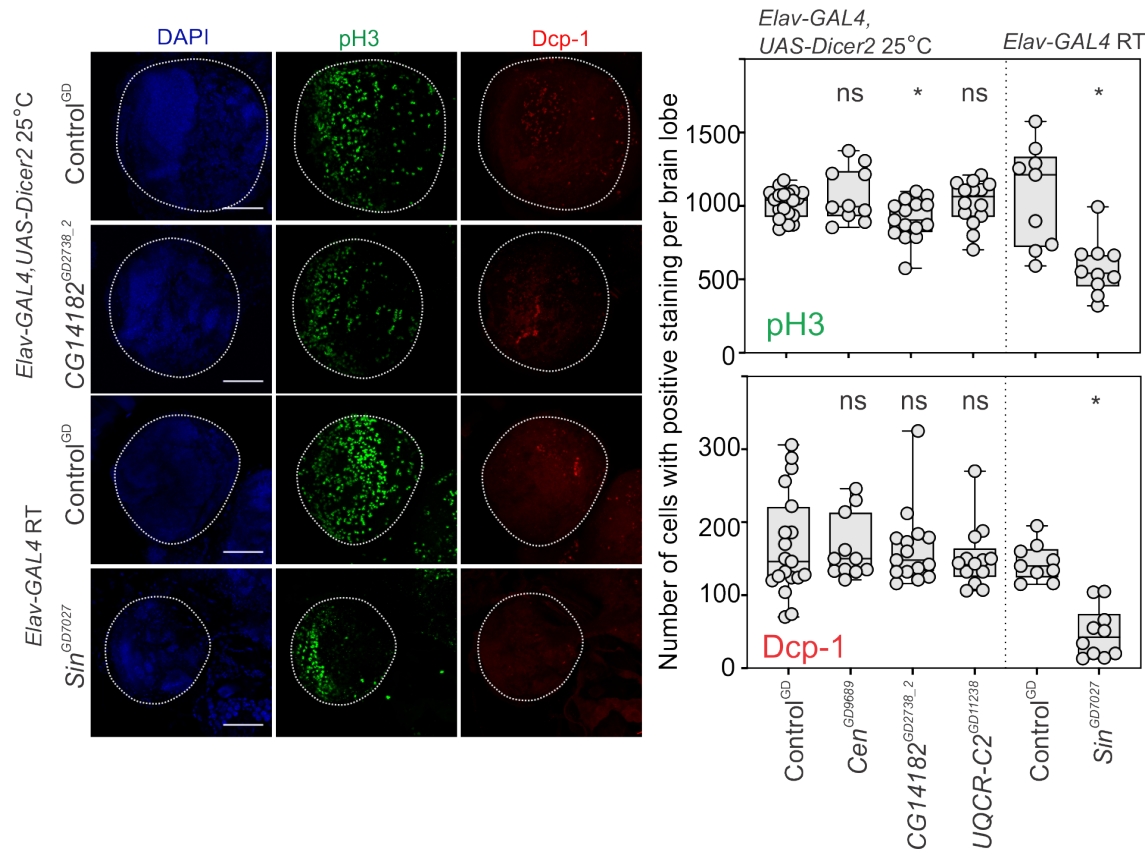

Supplement: S3 Fig — (A) Representative confocal brightfield images of nervous system-specific knockdown of 16p12.1 homologs show decreased total brain area for SinGD7027 and CG14182GD2738_2. Scale bars represent 100μm. (B) Elav-GAL4 mediated knockdown led to decreased number of phosphorylated histone-3 positive cells (pH3, green) in the brain lobe (DAPI, blue) with knockdown of SinGD7027 (n = 10, two-tailed Mann-Whitney, *p = 9.74×10^−4^) and CG14182GD2738_2 (n = 15, *p = 0.026), indicating decreased proliferation with knockdown of the homologs. Knockdown of SinGD7027 led to decreased number of Dcp-1 positive cells (*p = 2.78×10^−4^, red) in the brain lobe. Scale bar represents 50μm. Boxplots represent all data points with median, 25th and 75th percentiles. Statistical details, including sample size, confidence intervals, and p-values, are provided in S6 File. A list of full genotypes for fly crosses used in these experiments is provided in S1 File. (PDF) [file pgen.1009112.s003.pdf]

# **A** Craniofacial features with stronger knockdown

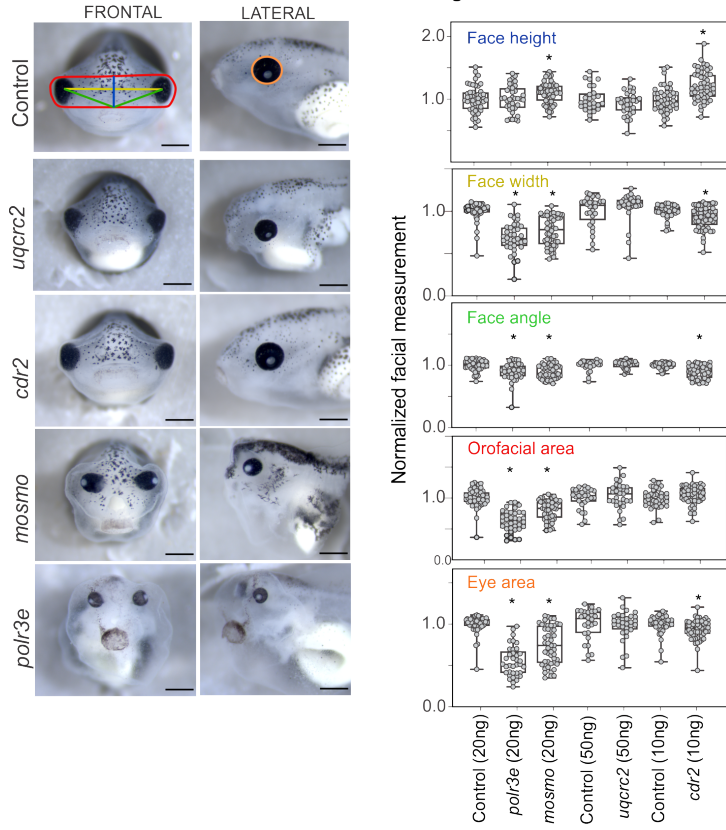

# **B**

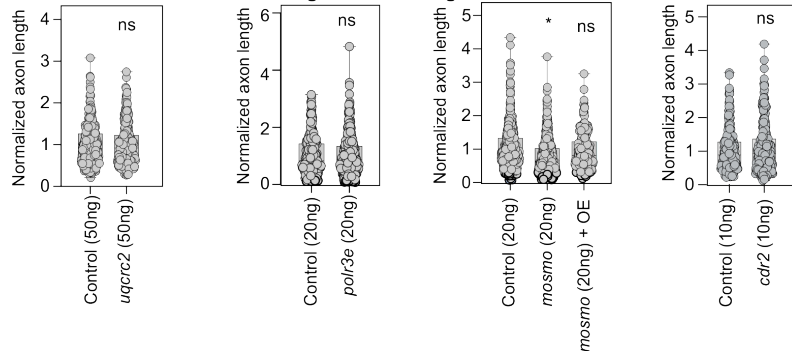

# **C**

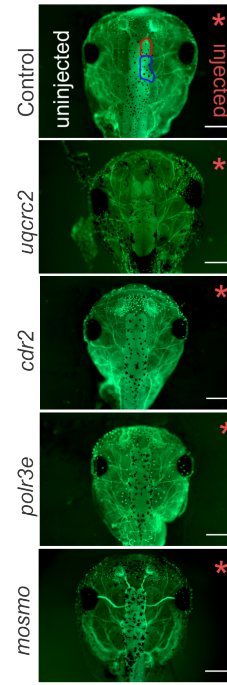

# Forebrain/midbrain area

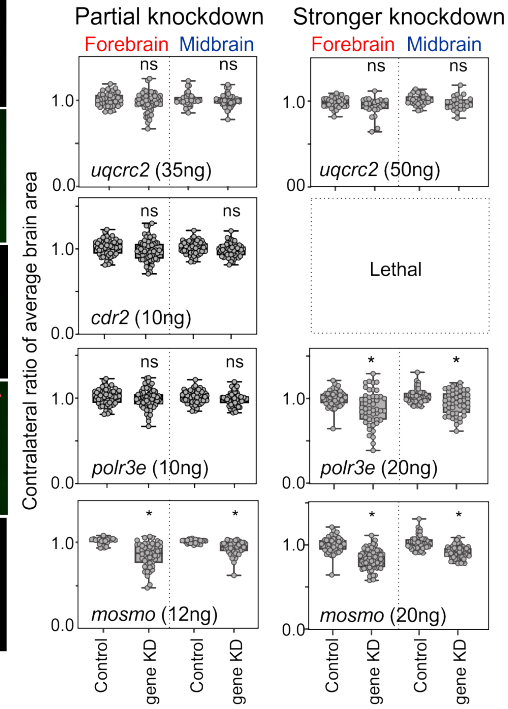

Supplement: S5 Fig — (A) Representative images of tadpoles injected with control morpholino or morpholinos for 16p12.1 homologs, indicating facial landmarks for face width (yellow), height (blue), angle (green), and orofacial (red) and eye (orange) area. Boxplots showing face height, width, angle, and orofacial and eye area of each knockdown compared to its own control. Knockdown of mosmo (n = 50, two-tailed student’s t-test, *p = 0.010) and cdr2 (n = 54, *p = 3.68×10^−6^) led to increased face height. Knockdown of cdr2 (*p = 7.75 ×10^−4^), polr3e (n = 37, *p = 1.97 ×10^−13^) and mosmo (*p = 1.36 ×10^−11^) led to decreased face width, while knockdown of cdr2 (*p = 1.03×10^−8^), polr3e (*p = 2.73×10^−4^) and mosmo (*p = 3.50×10^−7^) led to decreased face angle. Knockdown of polr3e (*p = 3.29 ×10^−16^) and mosmo (*p = 1.47 ×10^−8^) led to decreased orofacial area, and knockdown of polr3e (*p = 1.01×10^−18^), mosmo (*p = 7.23×10^−10^) and cdr2 (*p = 0.009) led to decreased eye area. Data represents strong knockdown of the 16p12.1 homologs, except for cdr2, which showed lethality and is shown for partial knockdown. All measures were normalized to their respective control injected with the same morpholino amount. Scale bars represent 500μm. (B) Boxplots showing axon length of each knockdown compared to its own control. Strong knockdown of mosmo led to decreased axon length in neural tube explants (n = 566, two-tailed student’s t-test, *p = 7.40 ×10^−12^), which was rescued by co-injection with overexpressed (OE) mRNA of the gene (n = 249, *p = 4.06×10^−5^). All measures were normalized to their respective control injected with the same morpholino amount. (C) Representative images stained with anti-tubulin show forebrain (red on control image) and midbrain (blue) areas of the side injected with morpholino (right, red asterisk), which were normalized to the uninjected side (left). Partial knockdown of mosmo led to decreased forebrain (n = 47, two tailed student’s t-test, *p = 1.18×10^−9^) and midbrain (*p = 1.45×10^−7^) ar [file pgen.1009112.s005.pdf]

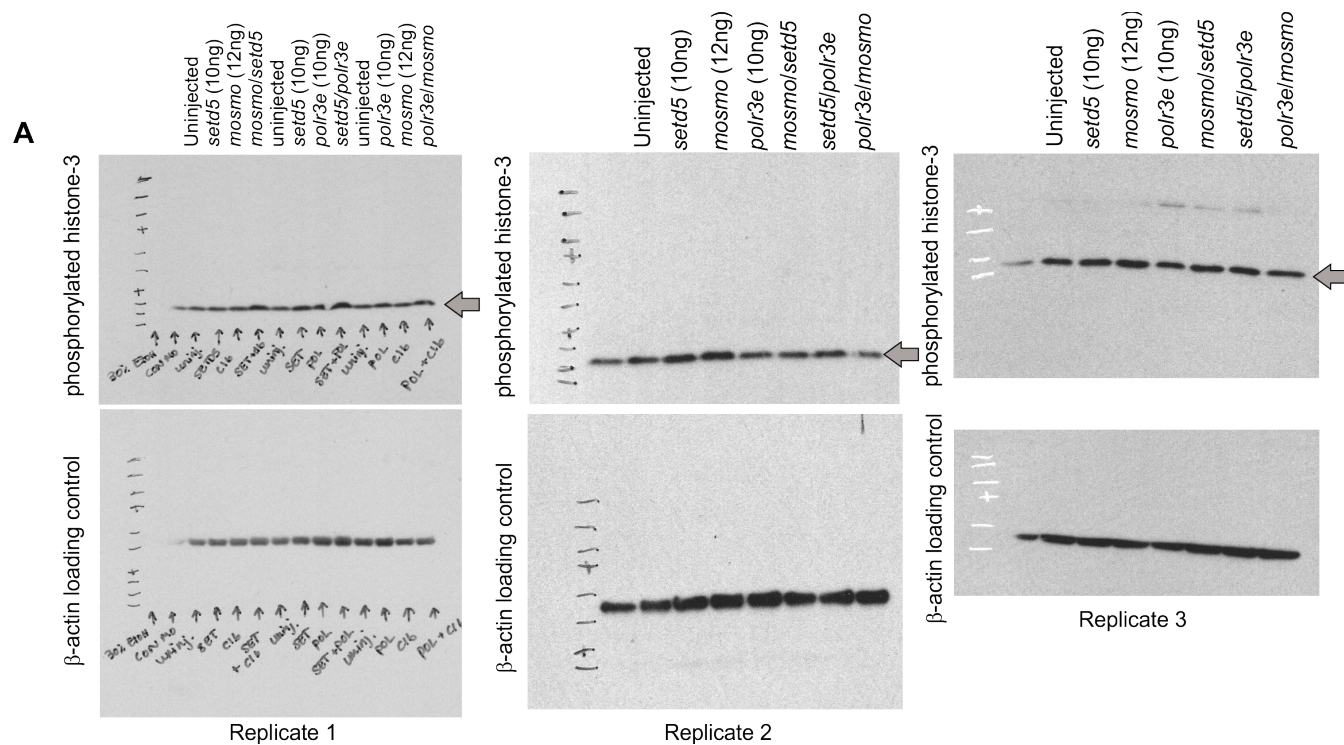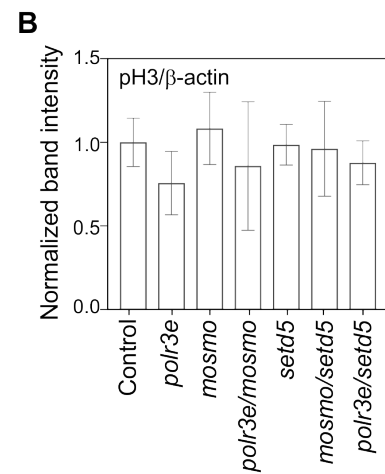

Supplement: S6 Fig — (A) Three replicate western blot experiments were performed. The intensity of bands at 17 kDa, corresponding with pH3 (top, indicated with arrow), were normalized to the β-actin loading control (bottom). (B) Partial knockdown of polr3e shows reduced band intensity with anti-pH3 antibody compared to β-actin loading control. Bar plot represents mean ± SD. (PDF) [file pgen.1009112.s006.pdf]

**A**

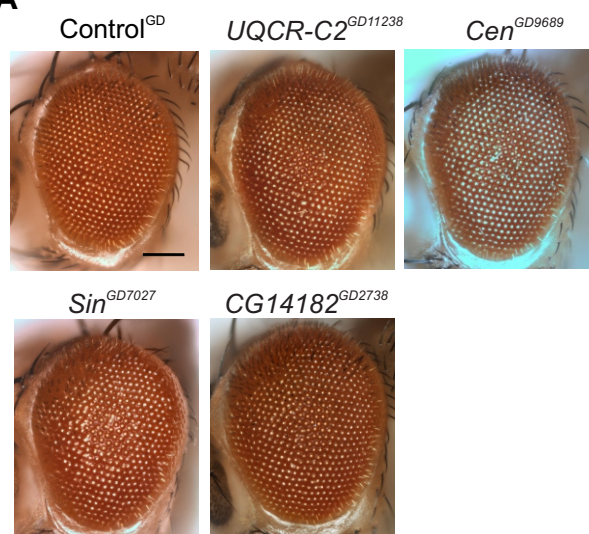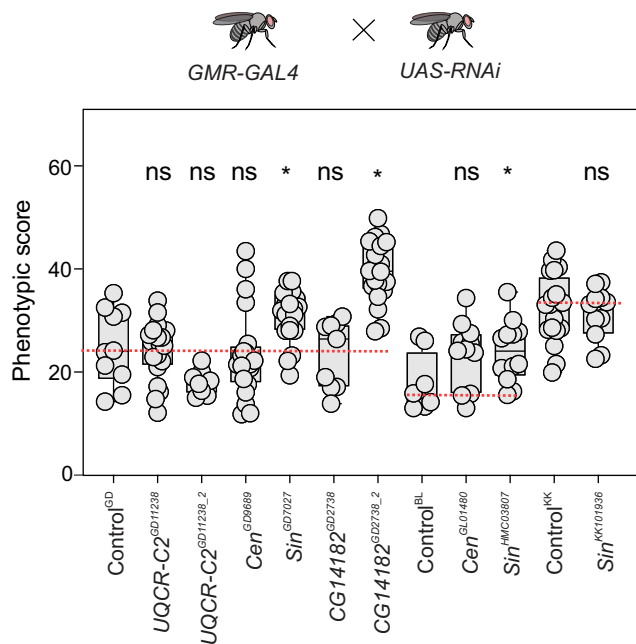

**B**

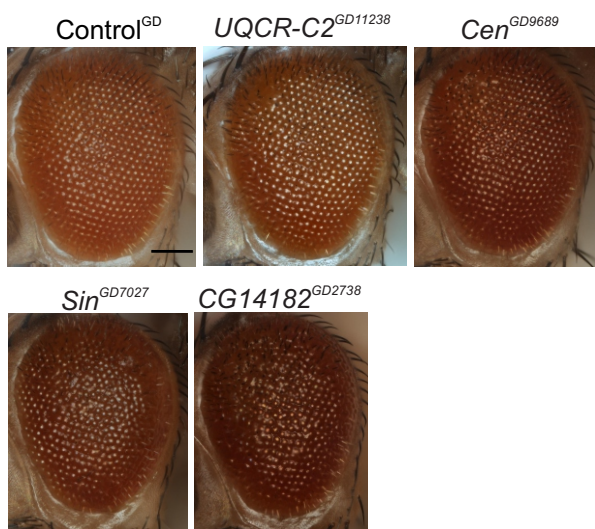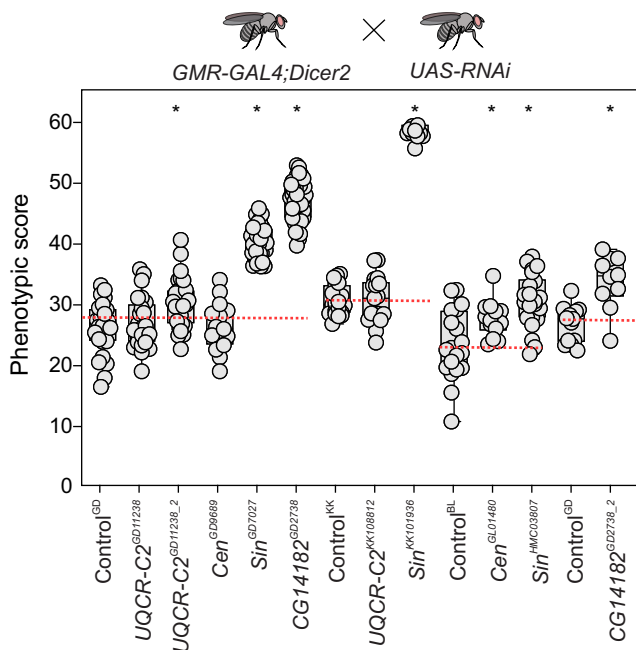

Supplement: S7 Fig — (A) Representative brightfield adult eye images and Flynotyper phenotypic scores of eye-specific knockdown of 16p12.1 homologs with GMR-GAL4 and no overexpression of Dicer2 at 30°C. A mild eye phenotype was observed with knockdown of Sin, replicated across multiple RNAi lines (two-tailed Mann-Whitney with Benjamini-Hochberg correction, *p<0.05). (B) Representative images and Flynotyper scores of eye-specific knockdown of 16p12.1 homologs with GMR-GAL4 and overexpression of Dicer2 at 30°C. Severe eye phenotypes were observed for all tested RNAi lines of Sin (*p< 1.13×10^−4^) and CG14182 (*p< 4.70×10^−4^). Scale bar represents 100 μm. Boxplots represent all data points with median, 25th and 75th percentiles, and red dotted lines indicate the control median. Statistical details, including sample size, confidence intervals, and p-values, are provided in S6 File. A list of full genotypes for fly crosses used in these experiments is provided in S1 File. (PDF) [file pgen.1009112.s007.pdf]

**A**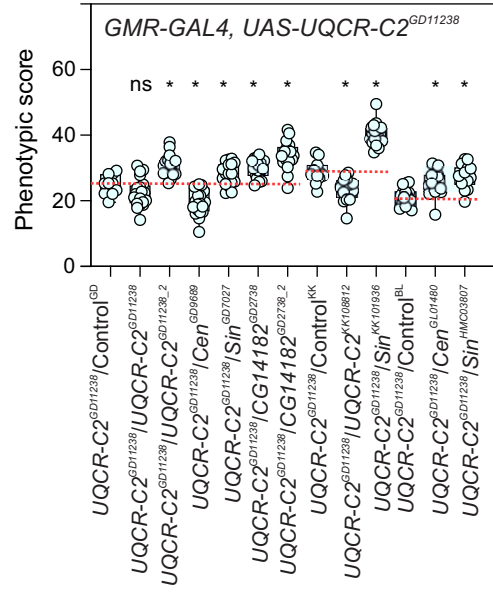**B**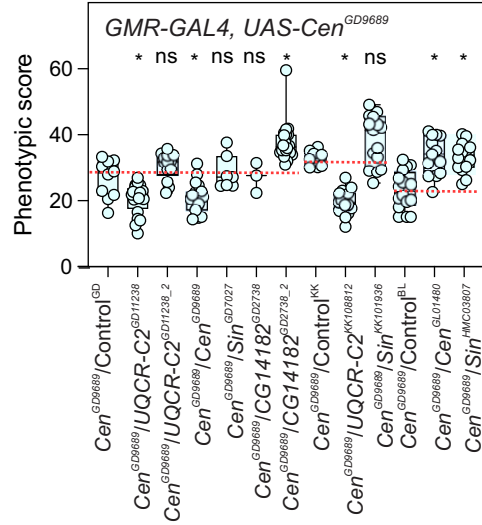**C**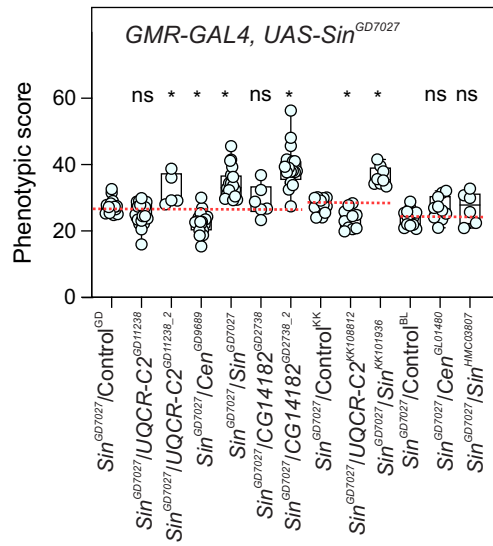**D**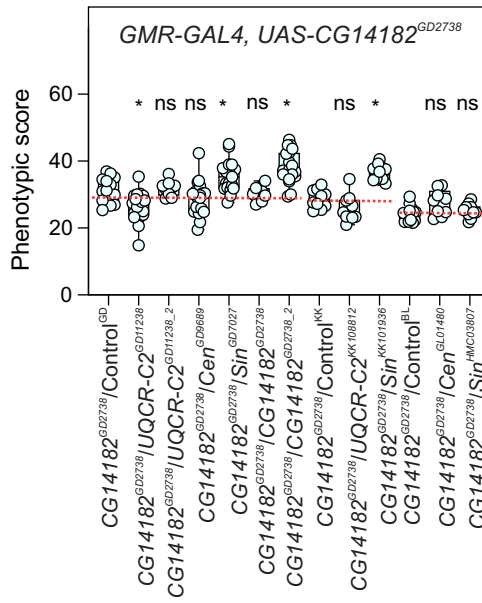

Supplement: S8 Fig — (A-D) Pairwise knockdown of homologs of 16p12.1 genes led to subtle changes in phenotypic scores, with only four significant combinations (compared to control lines) validated by multiple RNAi lines. Sin and CG14182 enhanced the eye phenotype of UQCR-C2GD11238, while Sin enhanced CG14182GD2738 eye phenotype and UQCR-C2 suppressed CenGD9689 eye phenotype (two-tailed Mann-Whitney with Benjamini-Hochberg correction, *p<0.05). Boxplots represent all data points with median, 25th and 75th percentiles. Red dotted lines indicate the median of recombinant lines crossed with control. Statistical details, including sample size, confidence intervals, and p-values, are provided in S6 File. A list of full genotypes for fly crosses used in these experiments is provided in S1 File. (PDF) [file pgen.1009112.s008.pdf]

# Distribution of flynotyper scores

Expected Observed

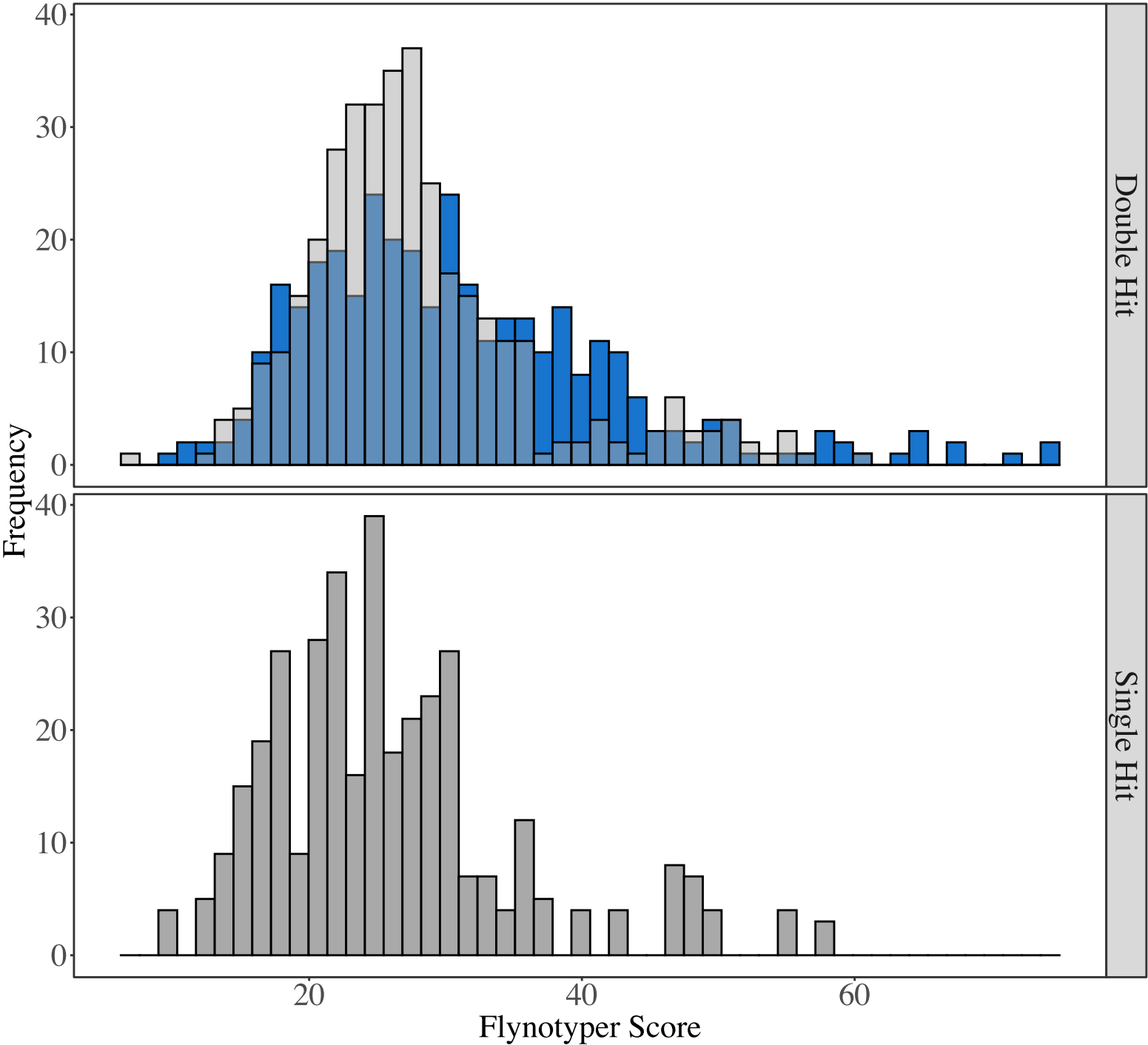

Supplement: S9 Fig — Histograms representing the distribution of observed (in grey) and expected (in blue) phenotypic scores of GMR-GAL4-mediated pairwise knockdowns of 16p12.1 homologs and interacting genes (top panel) and GMR-GAL4-mediated single knockdowns of potential interacting genes tested (bottom panel). The distribution shows an overlap of the observed and expected phenotypic scores values. (PDF) [file pgen.1009112.s009.pdf]

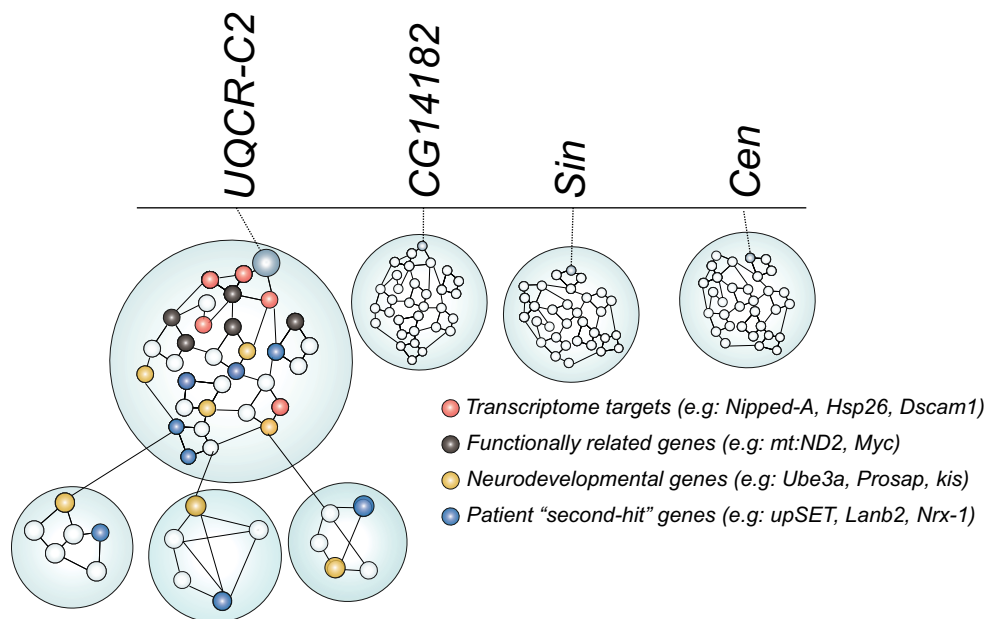

Supplement: S10 Fig — The diagram shows representative gene networks of each of the 16p12.1 homologs analyzed. UQCR-C2 is shown as an example, where the gene is closely connected to transcriptome targets and functionally related genes, while neurodevelopmental genes and patient-specific “second-hit” genes distribute more randomly between gene categories that can lead to positive and negative genetic interactions (top bubble) or combined independent effects (bottom bubble). These hypotheses aligned with our results, as we identified genetic interactions for 42/61 pairwise combinations (68.8%) with direct transcriptome targets, compared to 22/55 (40%) interactions identified with functionally related and neurodevelopmental genes (p = 0.0027, Fisher’s exact test). Moreover, we observed that homologs of genes carrying “second-hits” in severely affected children with the 16p12.1 deletion interacted with 16p12.1 homologs in 37/96 (38.5%) of the pairs, although the proportion of genetic interactions was not as high compared to those identified with functionally related genes or genes in neurodevelopmental pathways and transcriptome targets (64/101, 63%, Fisher’s exact test, p = 0.019). (PDF) [file pgen.1009112.s010.pdf]

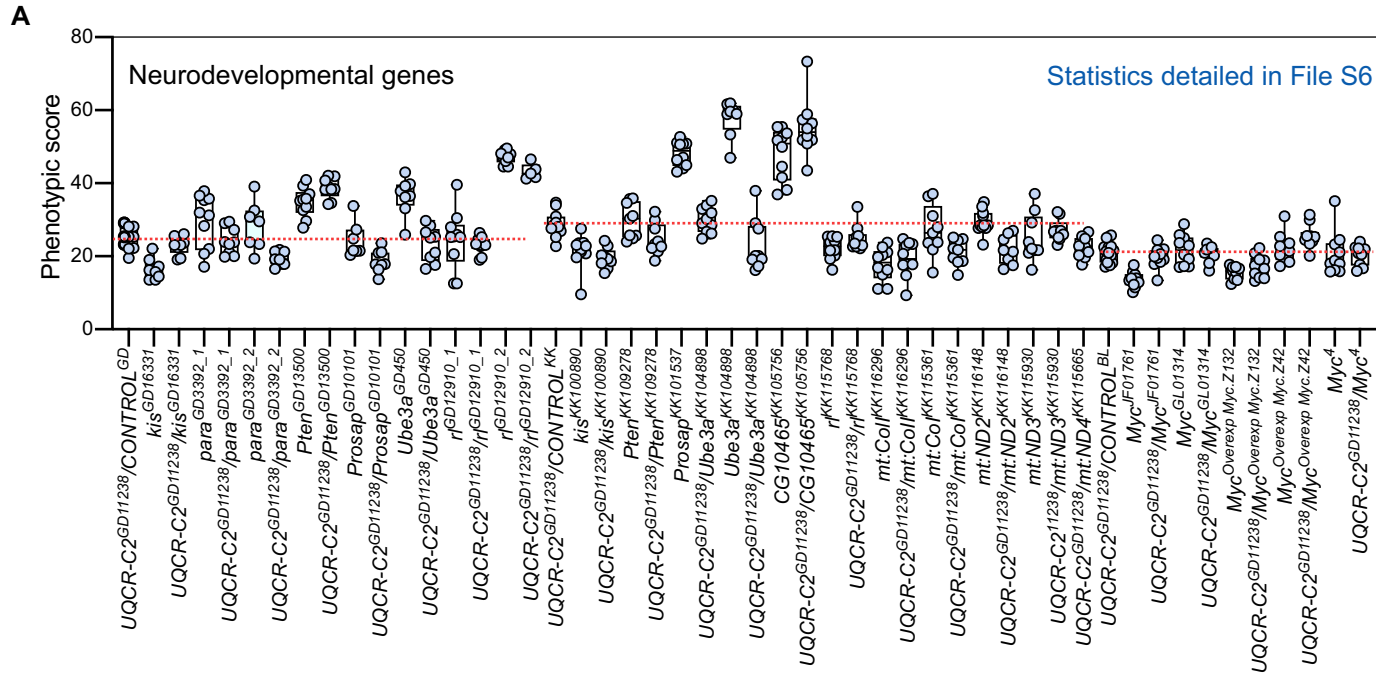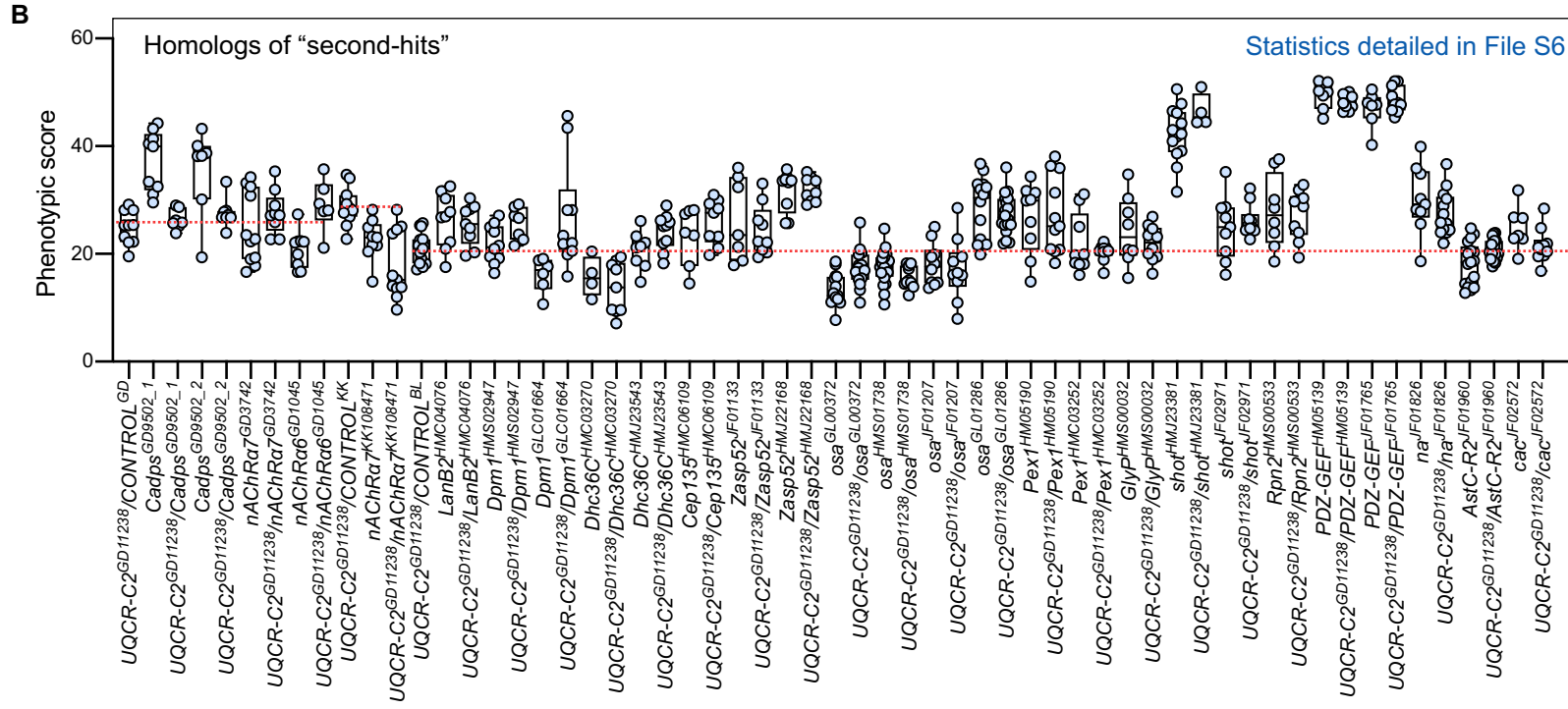

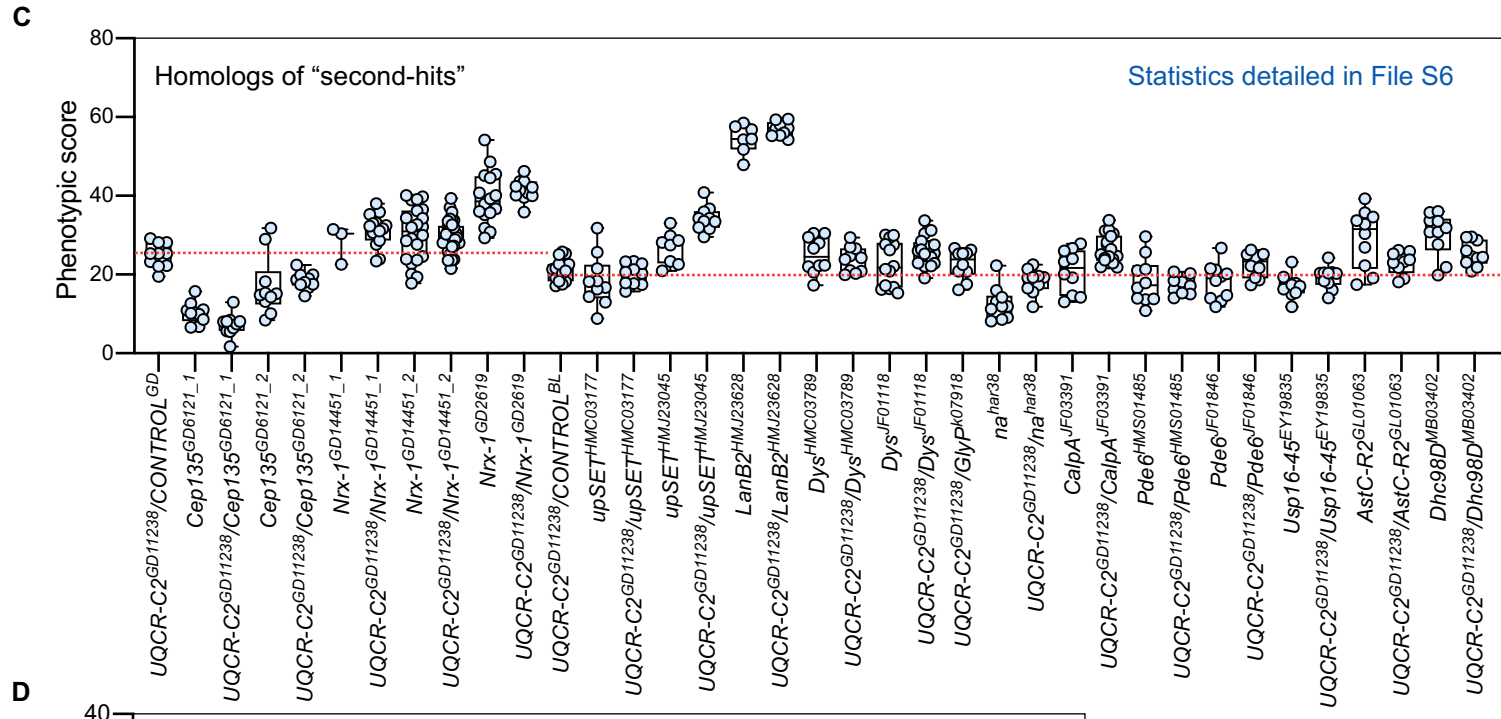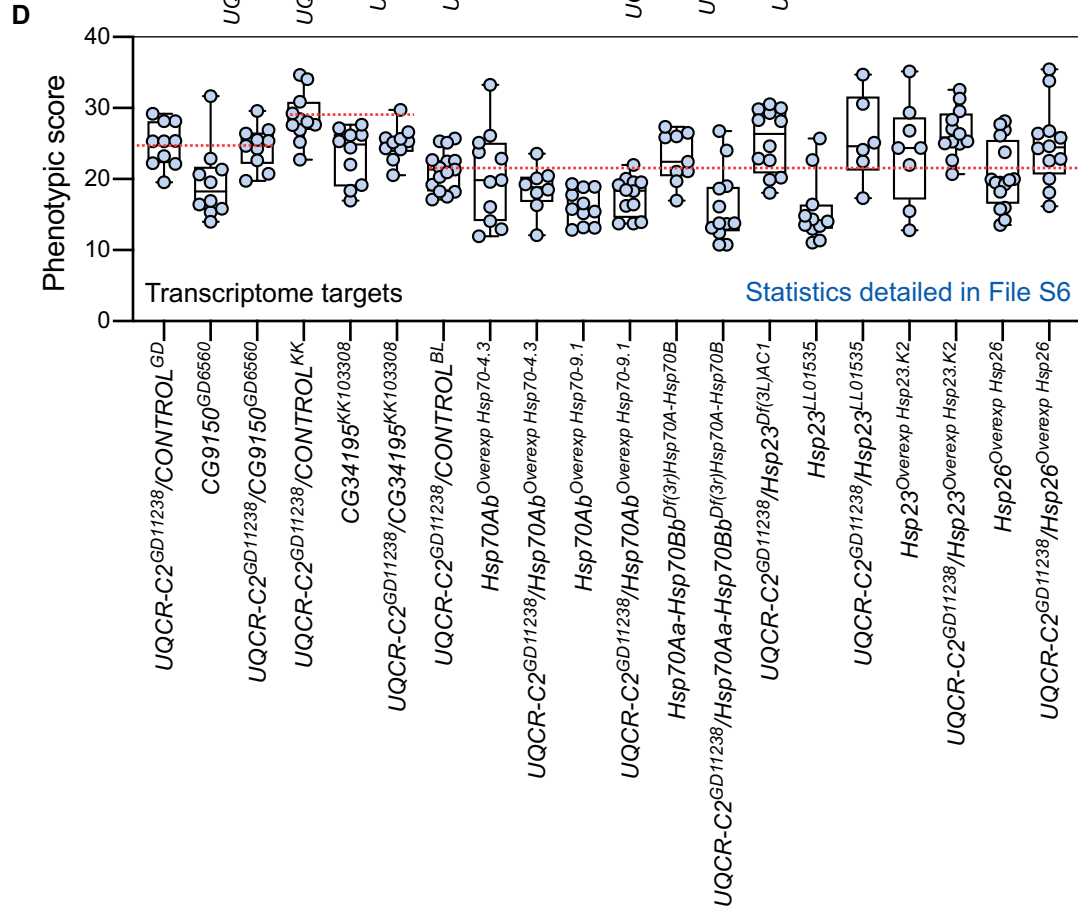

Supplement: S11 Fig — Flynotyper phenotypic scores of UQCR-C2GD11238 crossed with RNAi, mutant or overexpression lines of (A) neurodevelopmental genes or genes functionally related with UQCR-C2 function, (B and C) homologs of “second-hits” identified in children with 16p12.1 deletion, and (D) transcriptome targets and functionally related groups identified in RNA-sequencing of UQCR-C2 knockdown model. Boxplots represent all data points with median, 25th and 75th percentiles. Red dotted lines indicate the median of recombinant lines crossed with control. A list of full genotypes and statistics, including sample size, confidence intervals, and p-values, for these experiments are provided in S1 and S6 Files. (PDF) [file pgen.1009112.s011.pdf]

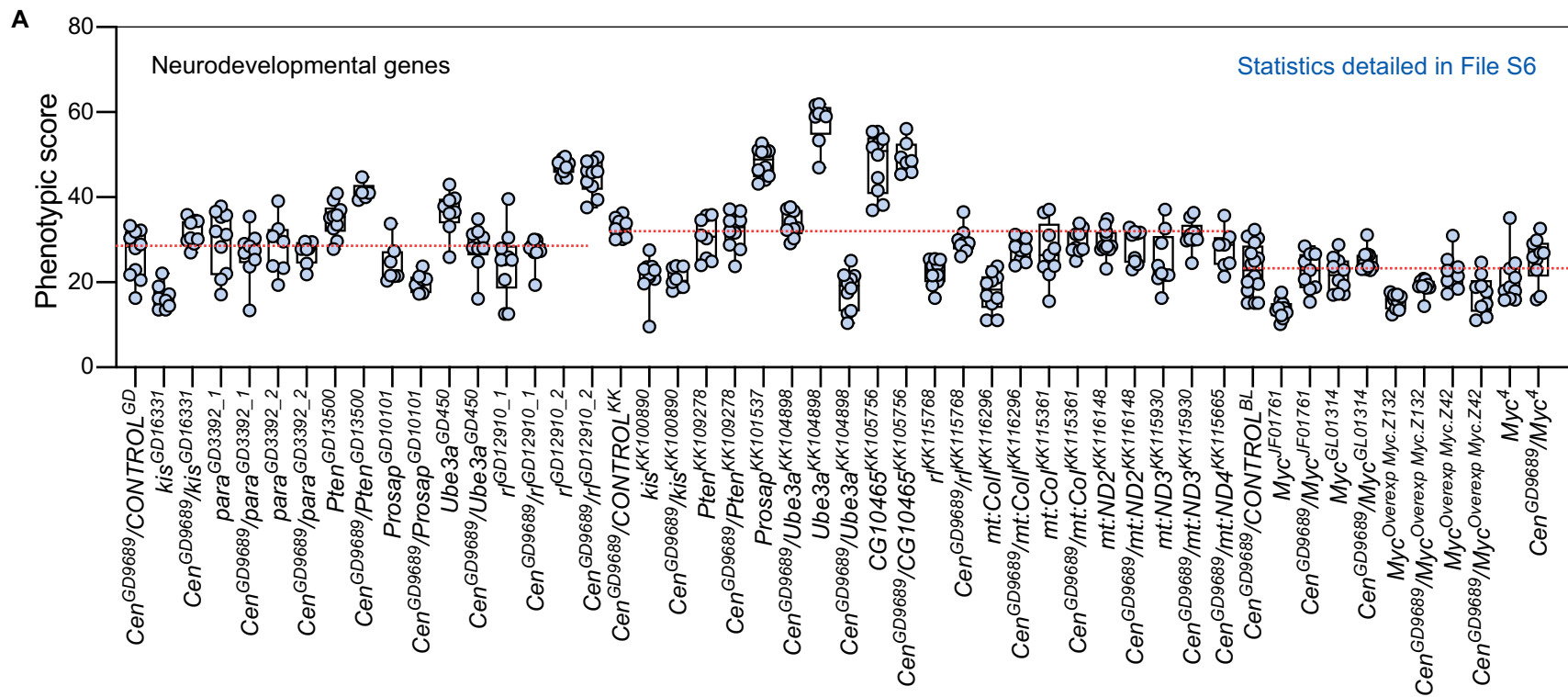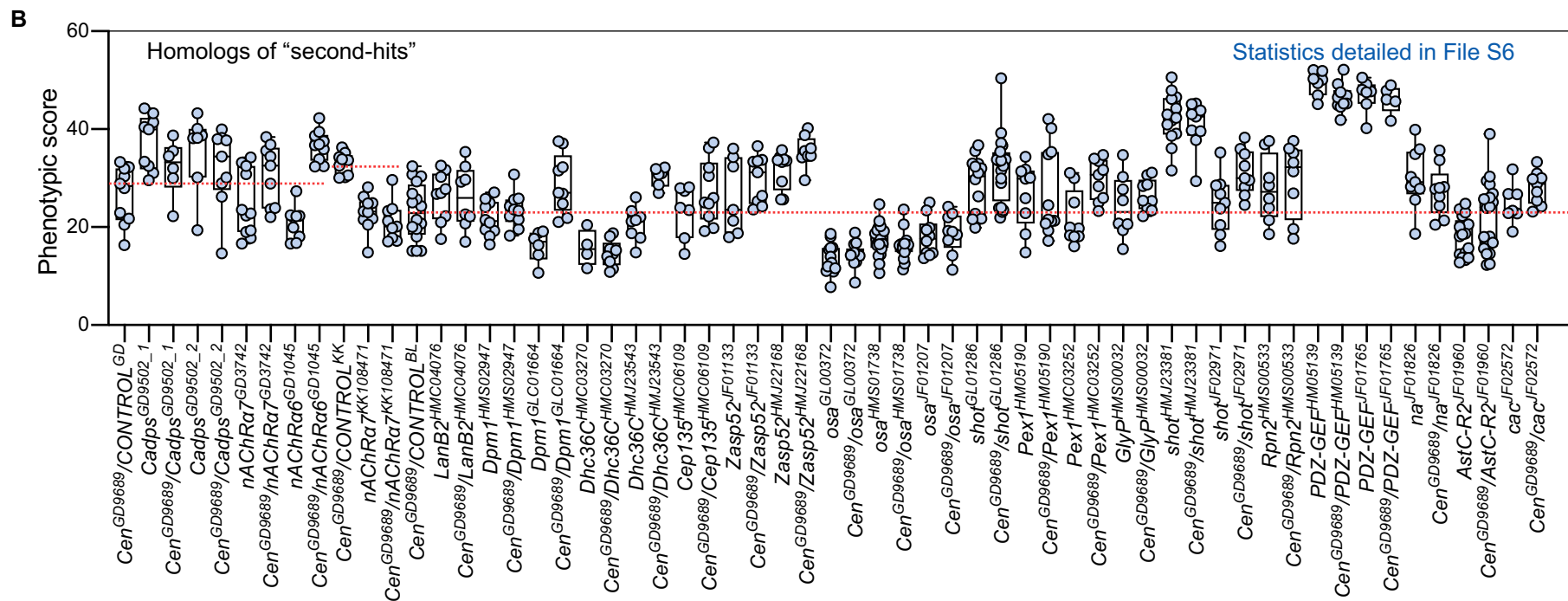

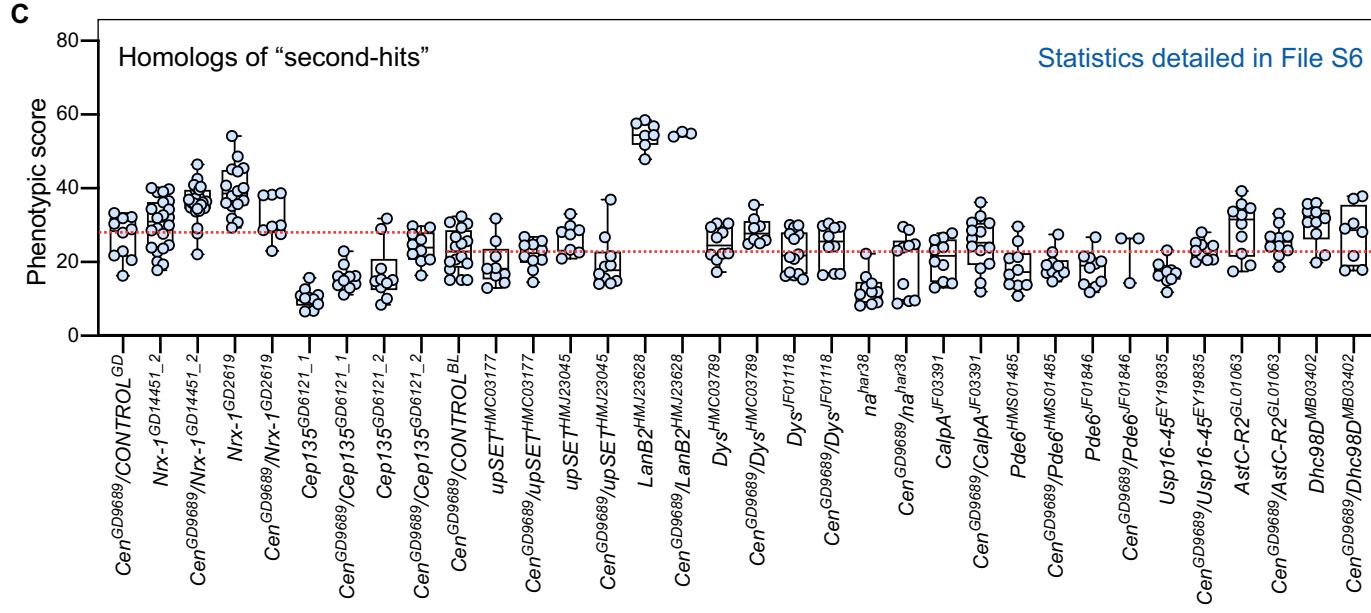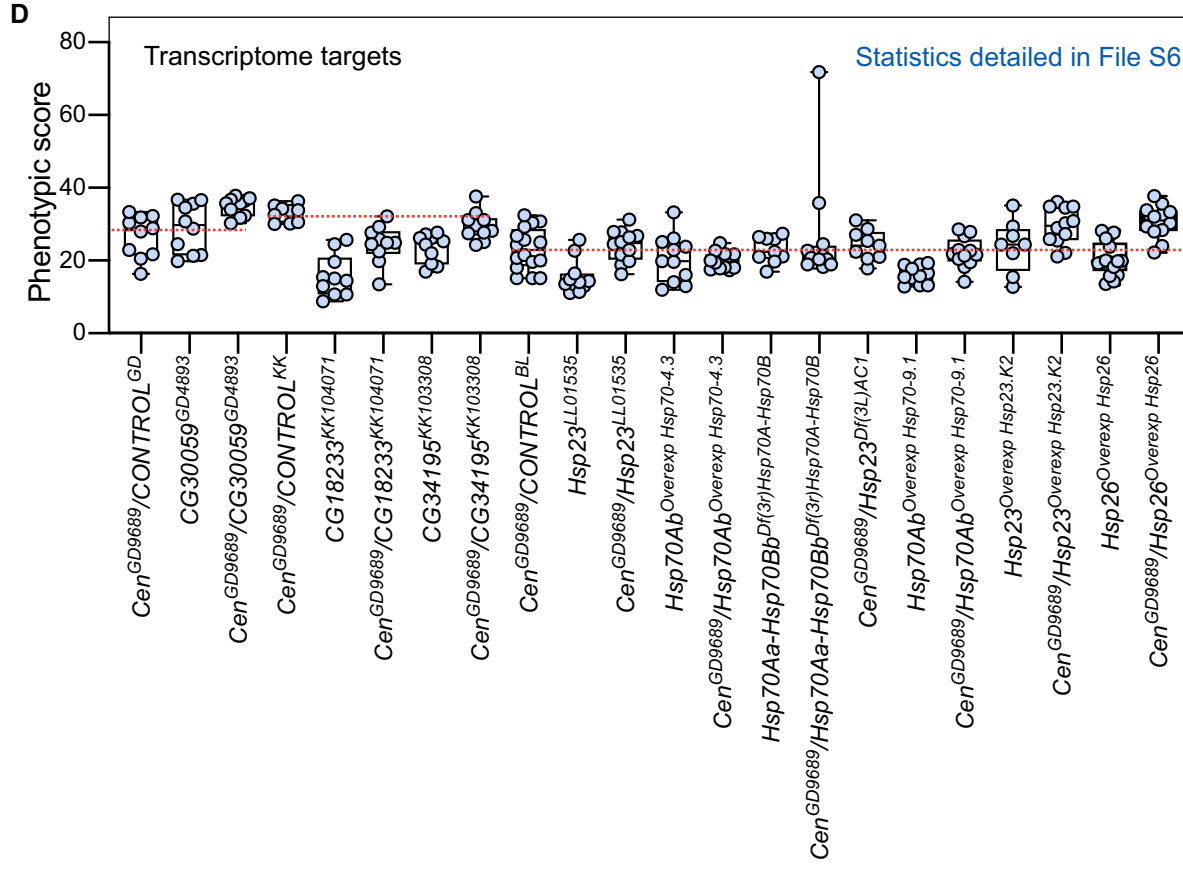

Supplement: S12 Fig — Flynotyper phenotypic scores of CenGD9689 crossed with RNAi, mutant or overexpression lines of (A) neurodevelopmental genes or genes functionally related with Cen function, (B and C) homologs of “second-hits” identified in children with 16p12.1 deletion, and (D) transcriptome targets and functionally related groups identified in RNA-sequencing of Cen knockdown model. Boxplots represent all data points with median, 25th and 75th percentiles. Red dotted lines indicate the median of recombinant lines crossed with control. A list of full genotypes and statistics, including sample size, confidence intervals, and p-values, for these experiments are provided in S1 and S6 Files. (PDF) [file pgen.1009112.s012.pdf]

**A**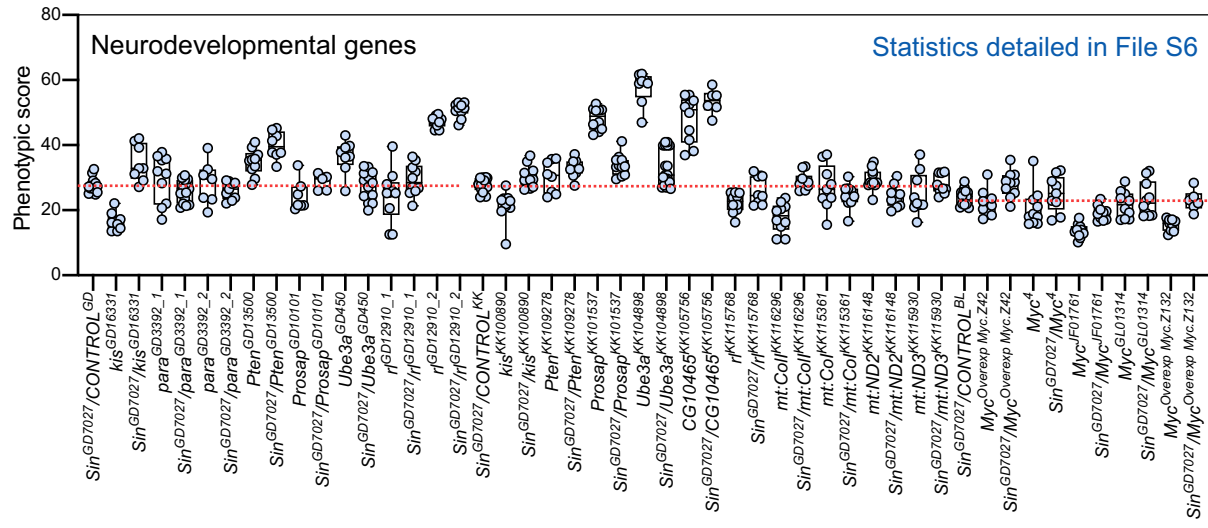**B**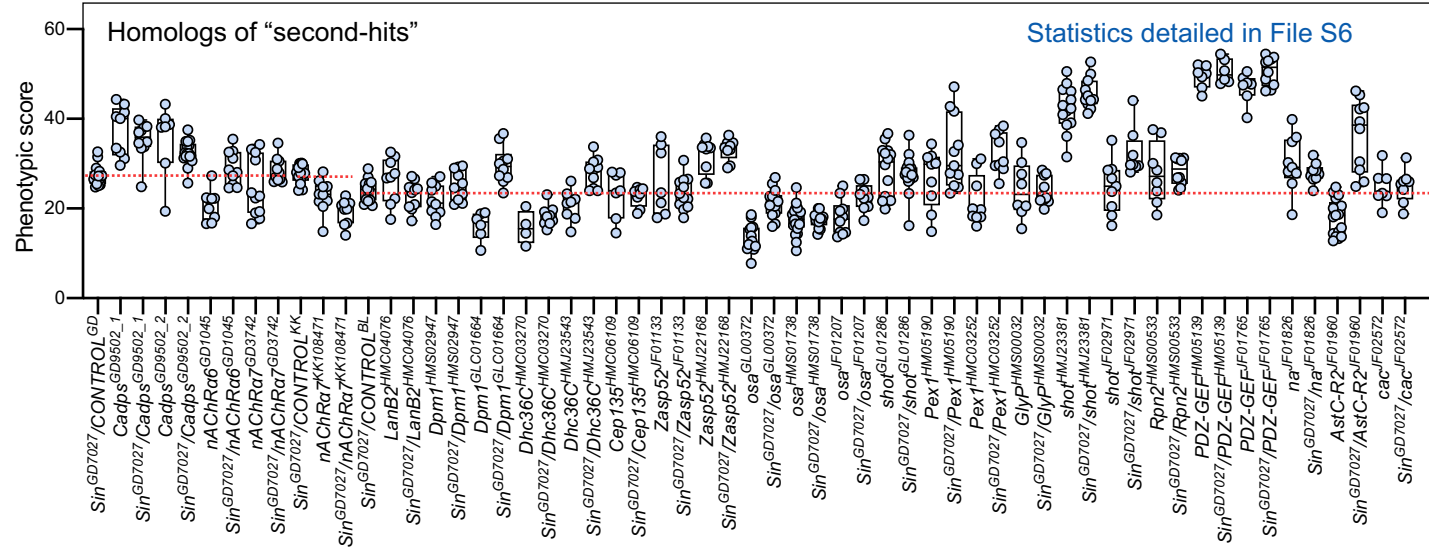**C**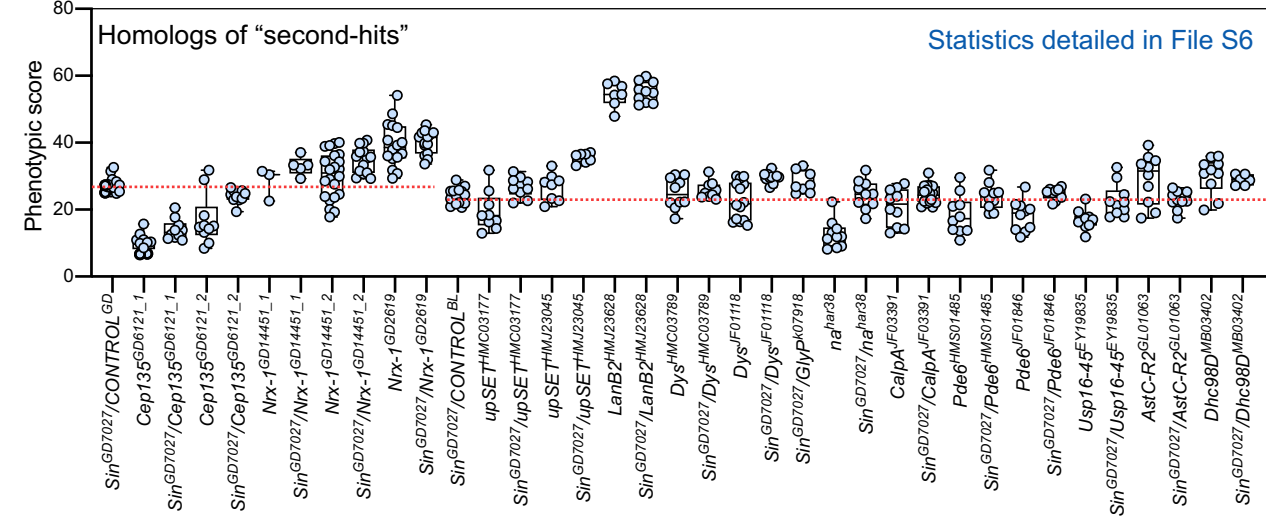

D

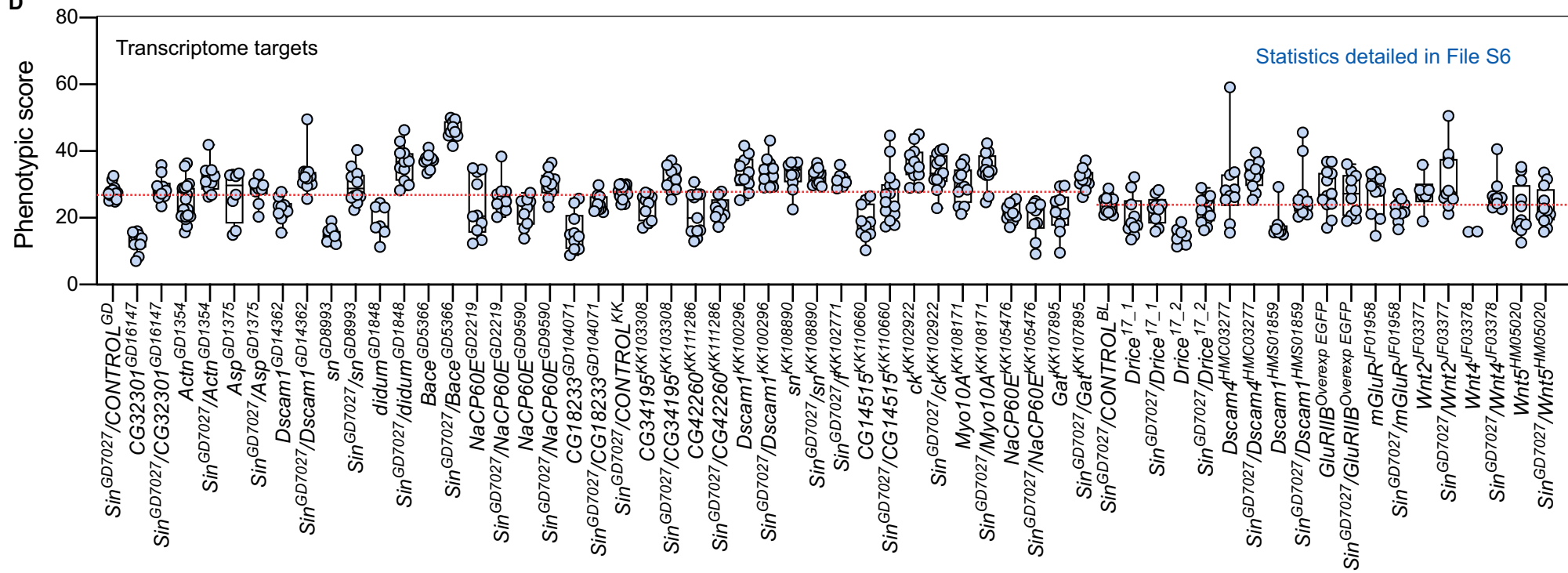

E

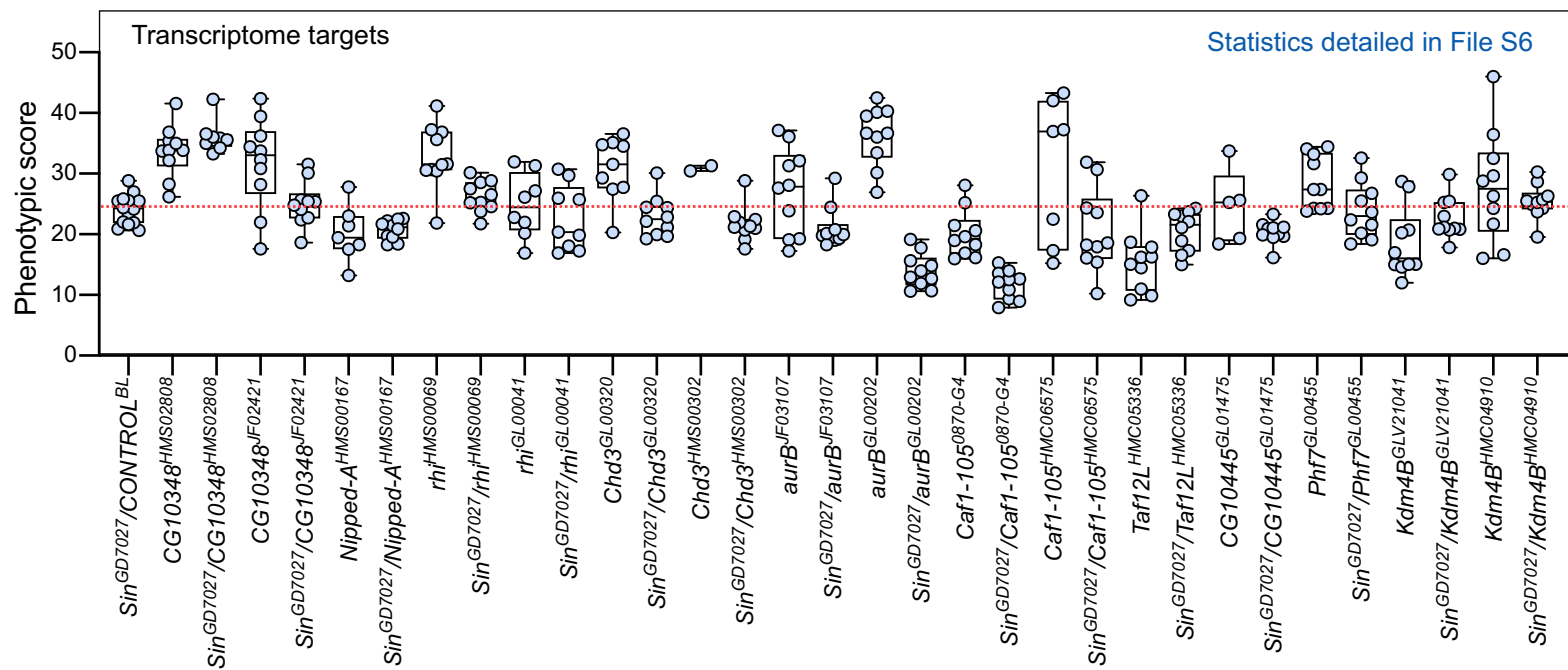

Supplement: S13 Fig — Flynotyper phenotypic scores of SinGD7027 crossed with RNAi, mutant or overexpression lines of (A) neurodevelopmental genes or genes functionally related with Sin function, (B and C) homologs of “second-hits” identified in children with 16p12.1 deletion, and (D and E) transcriptome targets and functionally related groups identified in RNA-sequencing of Sin knockdown model. Boxplots represent all data points with median, 25th and 75th percentiles. Red dotted lines indicate the median of recombinant lines crossed with control. A list of full genotypes and statistics, including sample size, confidence intervals, and p-values, for these experiments are provided in S1 and S6 Files. (PDF) [file pgen.1009112.s013.pdf]

A

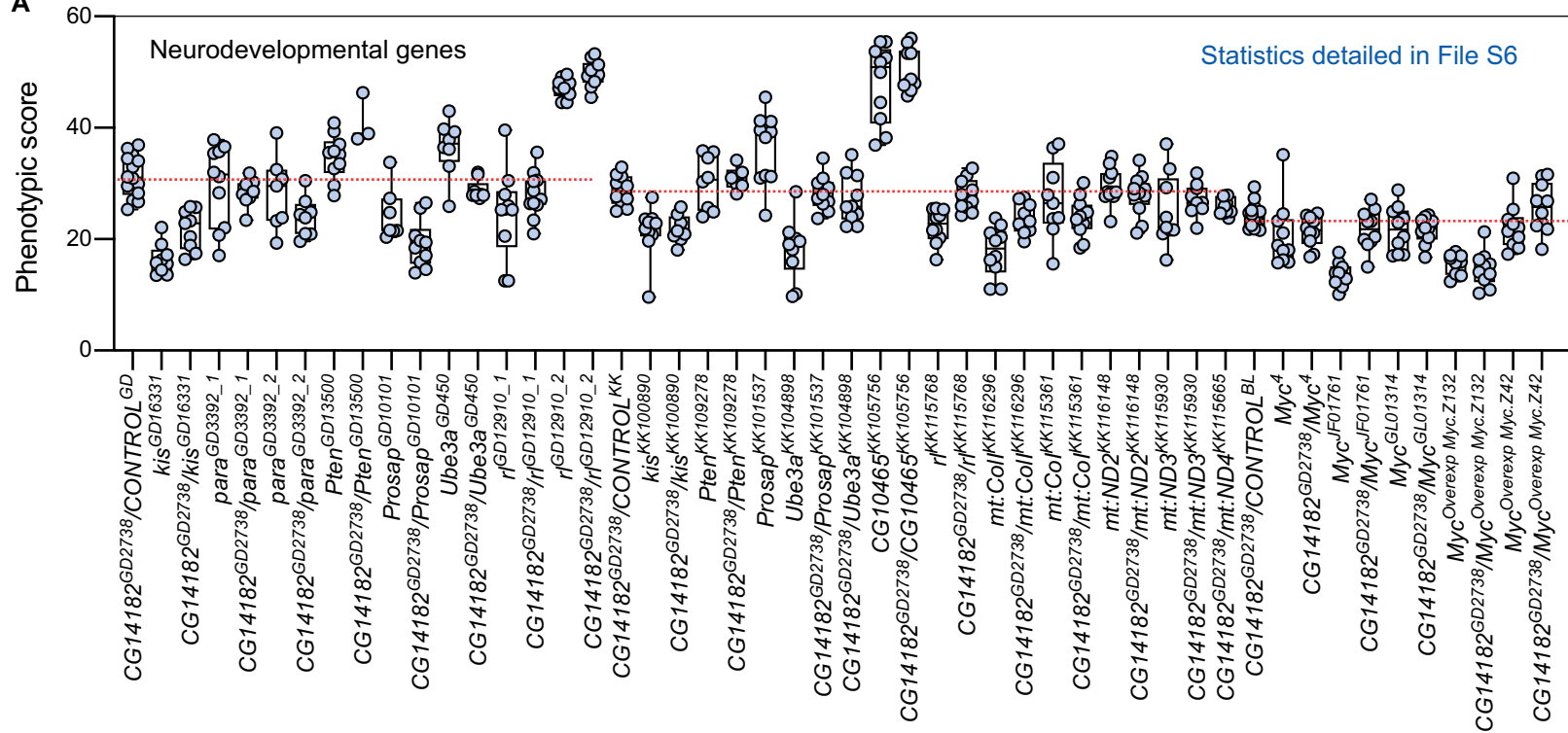

B

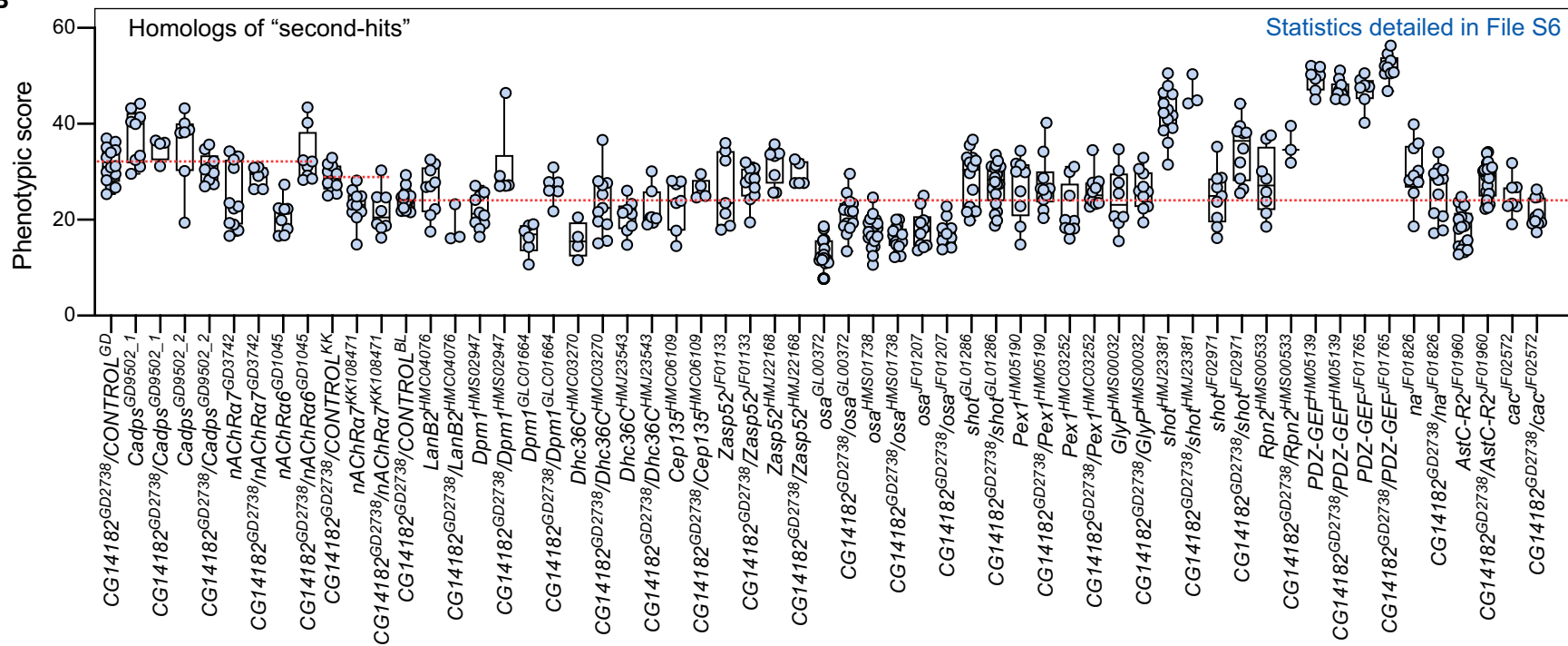

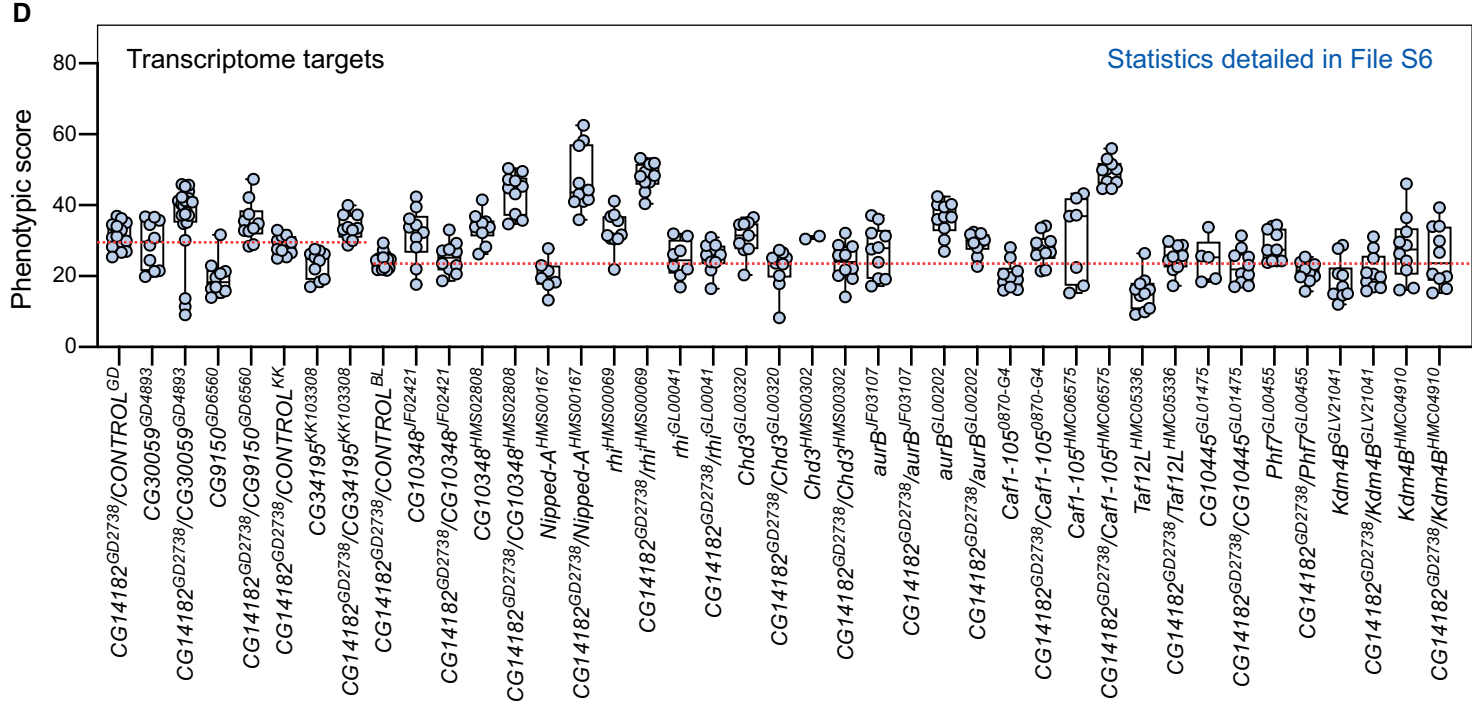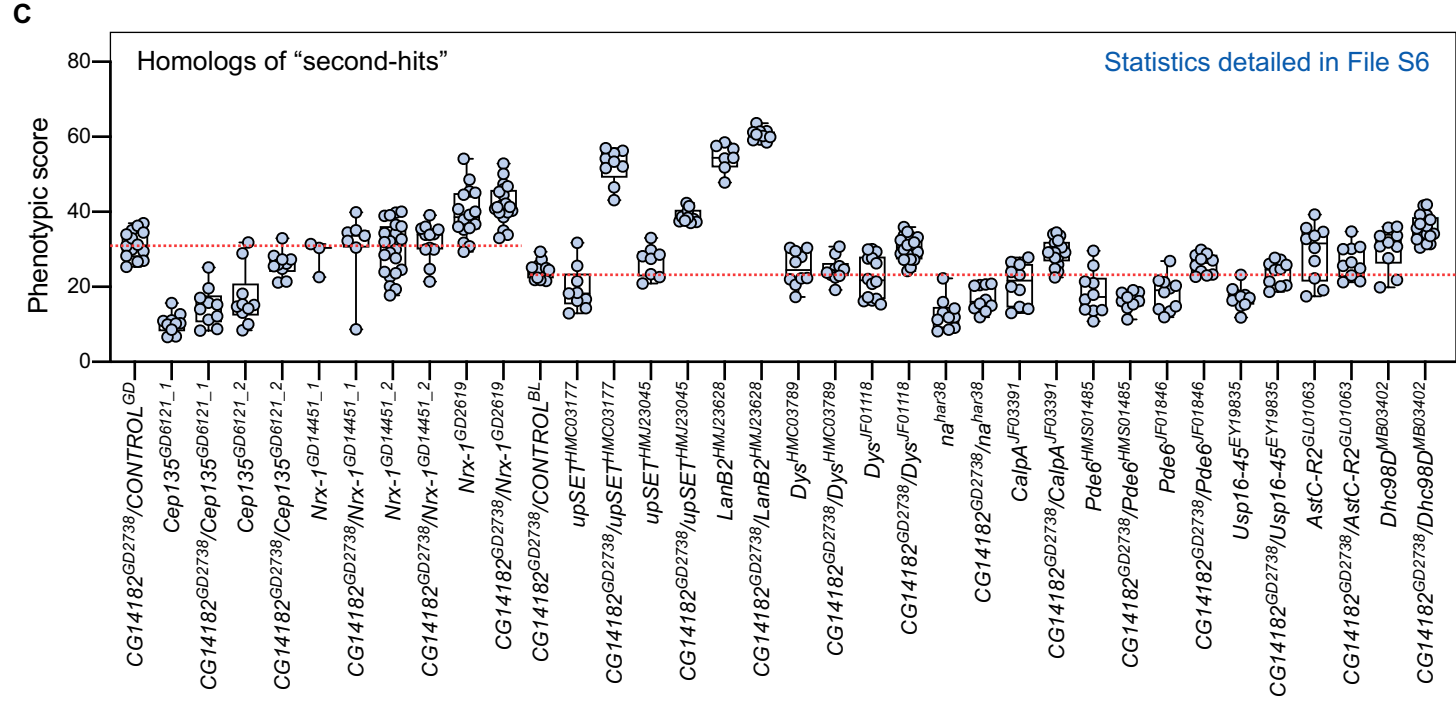

Supplement: S14 Fig — Flynotyper phenotypic scores of CG14182GD2738 crossed with RNAi, mutant or overexpression lines of (A) neurodevelopmental genes or genes functionally related with CG14182 function, (B and C) homologs of “second-hits” identified in children with 16p12.1 deletion, and (D) transcriptome targets and functionally related groups identified in RNA-sequencing of CG14182 knockdown model. Boxplots represent all data points with median, 25th and 75th percentiles. Red dotted lines indicate the median of recombinant lines crossed with control. A list of full genotypes and statistics, including sample size, confidence intervals, and p-values, for these experiments are provided in S1 and S6 Files. (PDF) [file pgen.1009112.s014.pdf]

**A**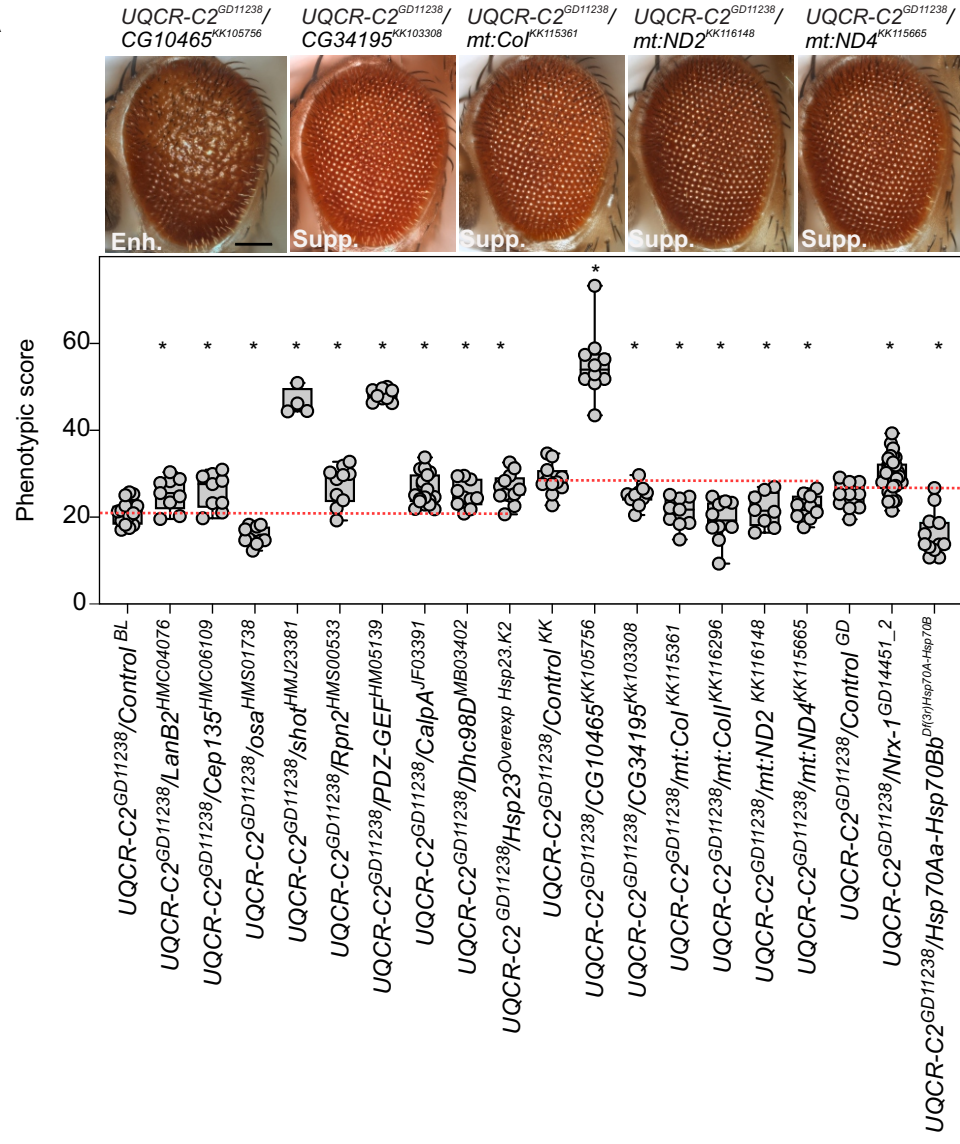**B**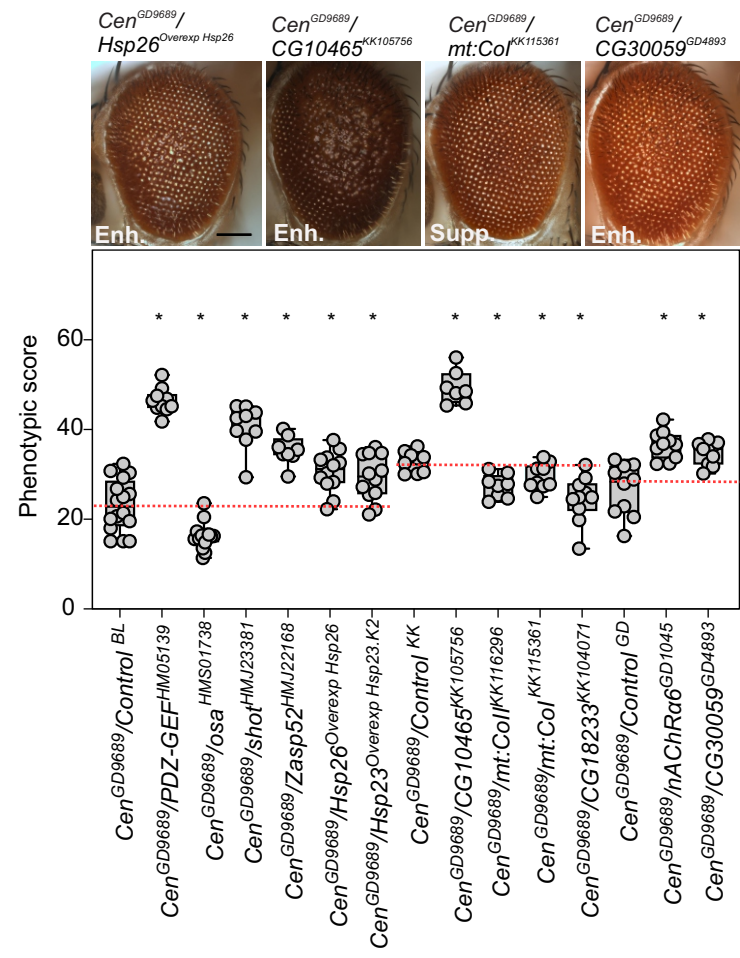

Supplement: S15 Fig — Representative brightfield adult eye images and phenotypic scores of eyes from RNAi, mutant or overexpression lines of neurodevelopmental genes, homologs of genes with “second-hits” in children with 16p12.1 deletion, and transcriptome targets that significantly enhanced (Enh.) or suppressed (Supp.) the phenotypes of recombinant lines of UQCR-C2GD11238 (A) or CenGD9689 (B) (*p<0.05, two-tailed Mann-Whitney tests with Benjamini-Hochberg correction). Scale bar represents 100 μm. Boxplots represent all data points with median, 25th and 75th percentiles. Red dotted lines indicate the median of recombinant lines crossed with control. A list of full genotypes and statistics, including sample size, confidence intervals, and p-values, for these experiments are provided in S1 and S6 Files. (PDF) [file pgen.1009112.s015.pdf]

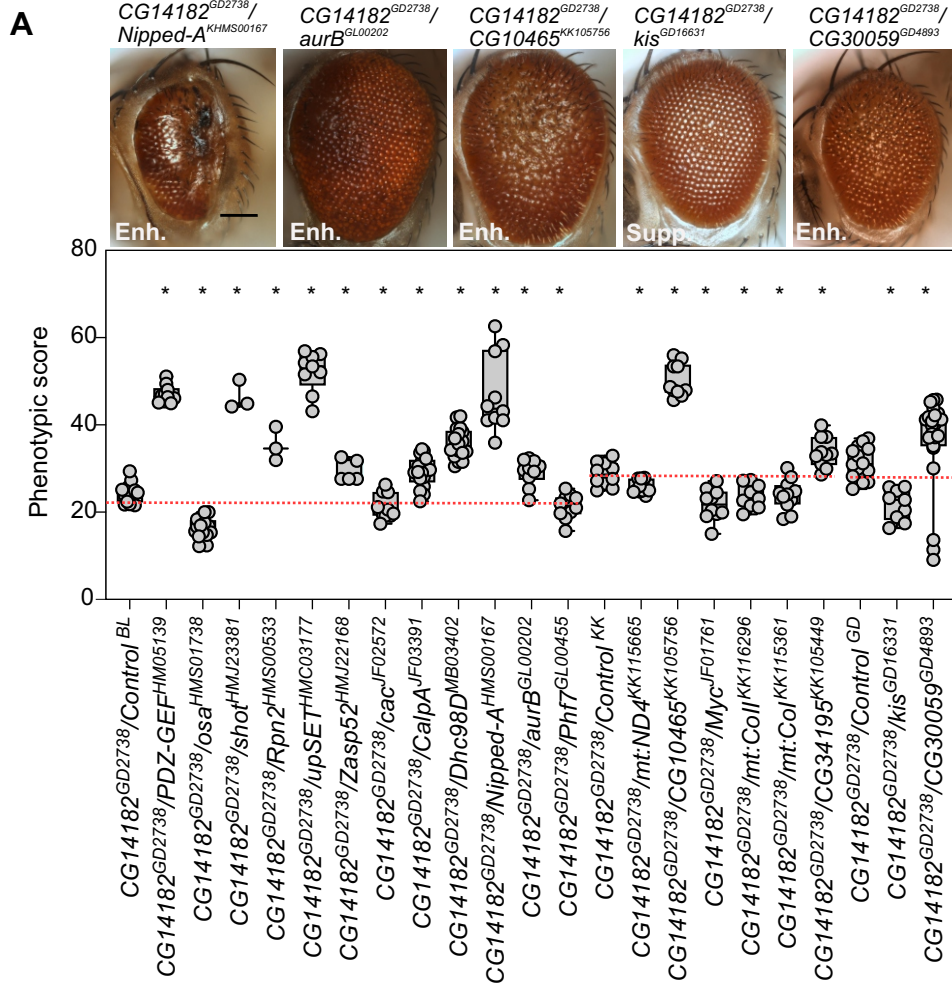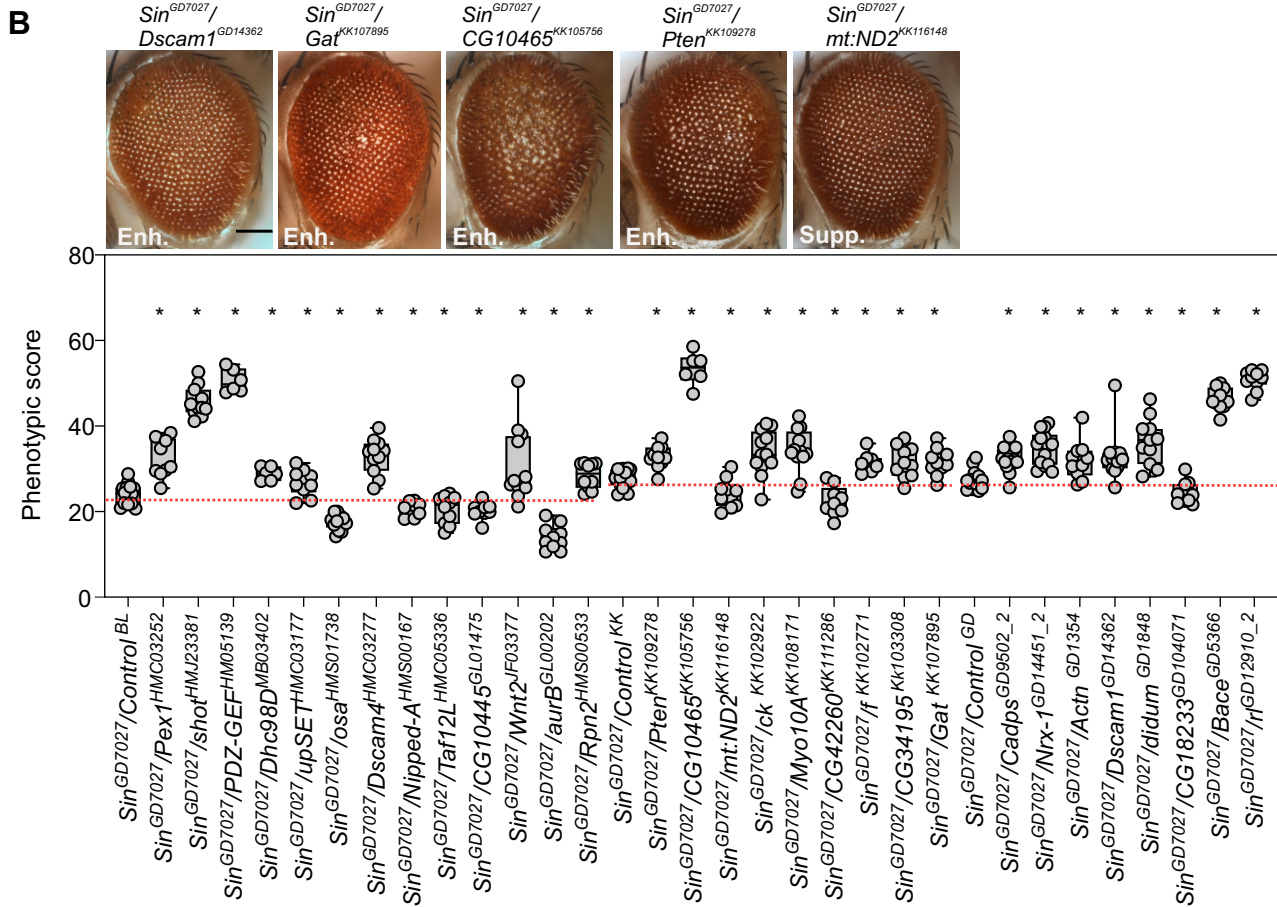

Supplement: S16 Fig — Representative brightfield adult eye images and phenotypic scores of eyes from RNAi, mutant or overexpression lines of neurodevelopmental genes, homologs of genes with “second-hits” in children with 16p12.1 deletion, and transcriptome targets that significantly enhanced (Enh.) or suppressed (Supp.) the phenotypes of recombinant lines of CG14182GD2738 (A) or SinGD7027 (B) (*p<0.05, two-tailed Mann-Whitney tests with Benjamini-Hochberg correction). Scale bar represents 100 μm. Boxplots represent all data points with median, 25th and 75th percentiles. Red dotted lines indicate the median of recombinant lines crossed with control. A list of full genotypes and statistics, including sample size, confidence intervals, and p-values, for these experiments are provided in S1 and S6 Files. (PDF) [file pgen.1009112.s016.pdf]

**A**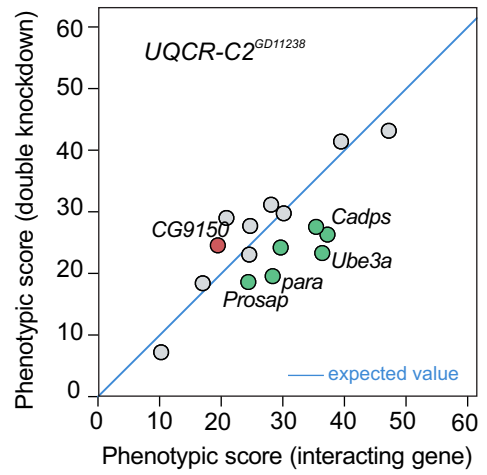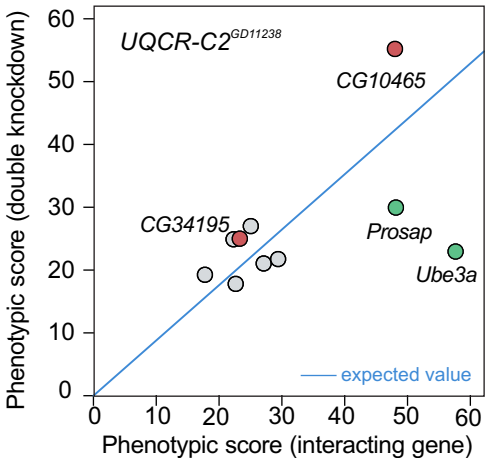**B**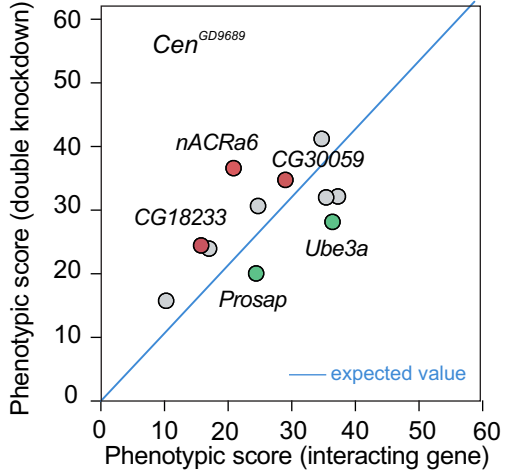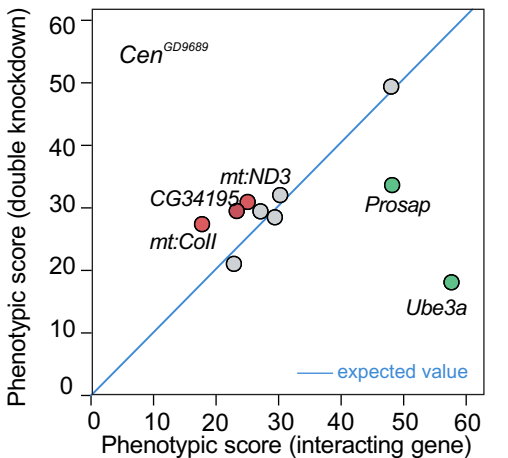**C**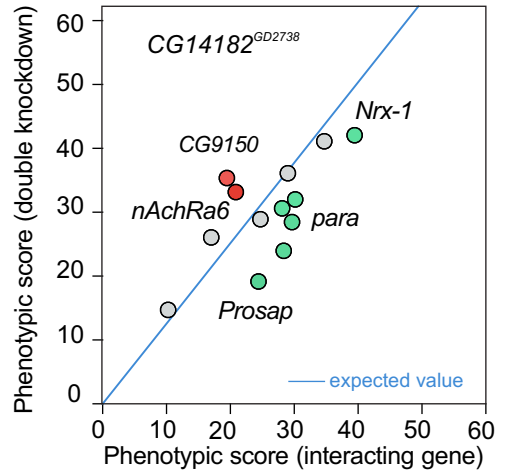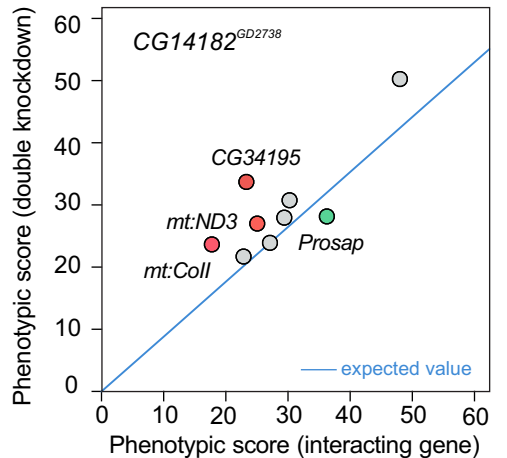**D**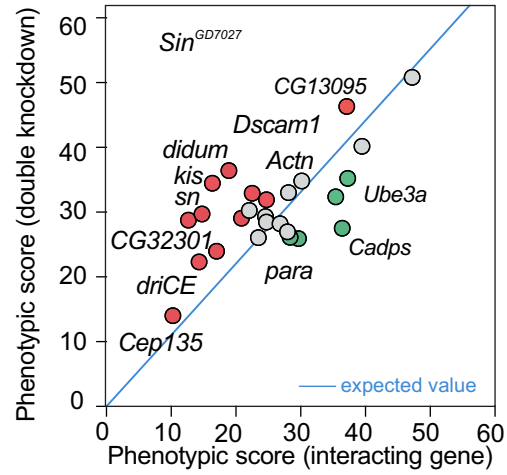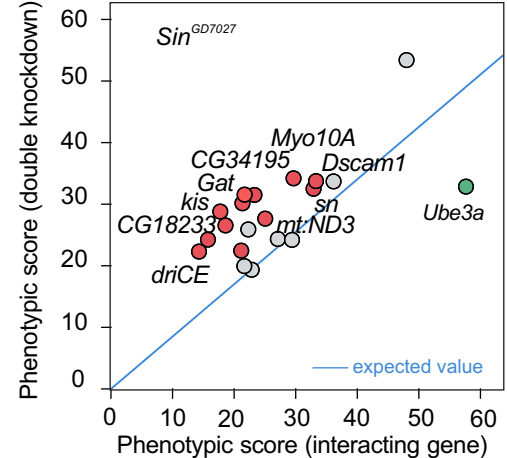

Supplement: S17 Fig — Scatter plots depict the interactions tested for GMR-GAL4 recombinant lines for UQCR-C2GD11238 (A), CenGD9689 (B), CG14182GD2738 (C), and SinGD7027 (D). The plots show the average phenotypic score of the interacting gene on the x-axis using VDRC GD (top) or KK (bottom) fly lines and the average observed phenotypic score for the double knockdown on the y-axis. Blue line represents the expected phenotypic score of the pairwise knockdown calculated as the product of the first hit phenotype (Flynotyper score of first hit crossed with control, such as UQCR-C2GD11238 X ControlGD) for each theoretical phenotypic value of interacting gene (ranging from 0 to 60) represented on x-axis. All positive and negative (validated or potential) interactions are represented in green and red, respectively, and fly lines of genes with no significant interactions are shown in grey. (PDF) [file pgen.1009112.s017.pdf]

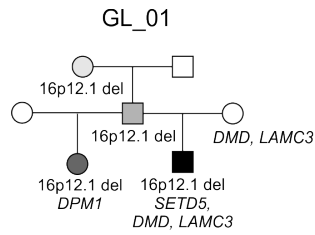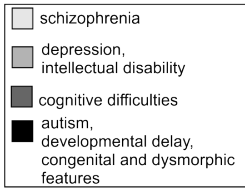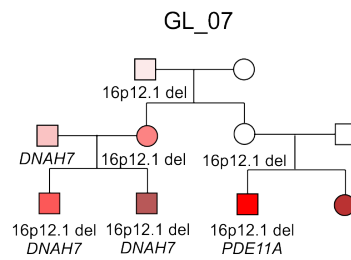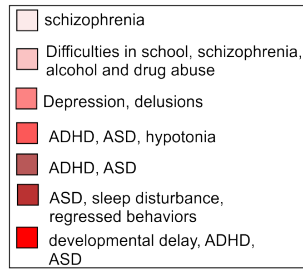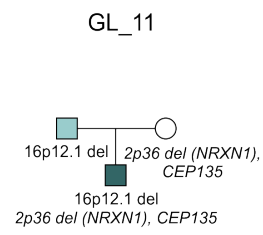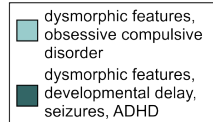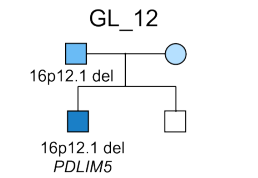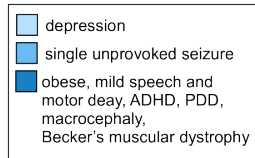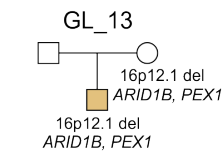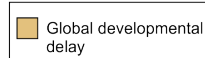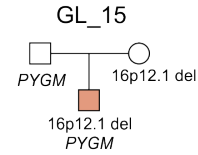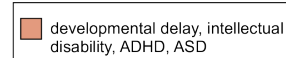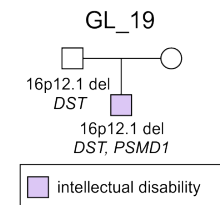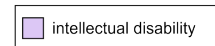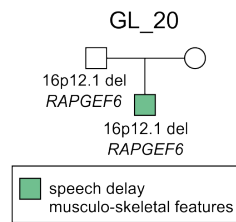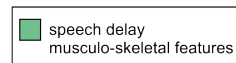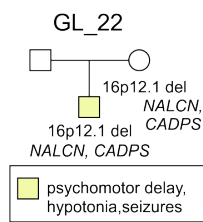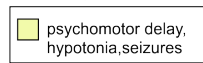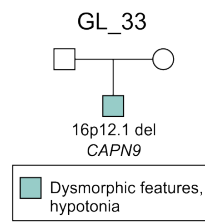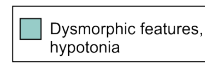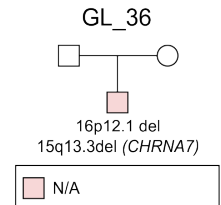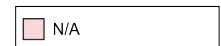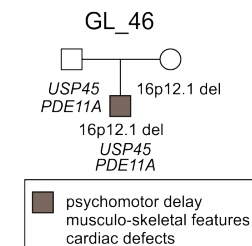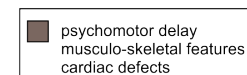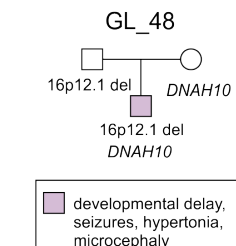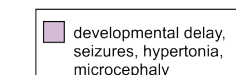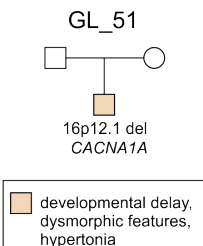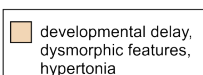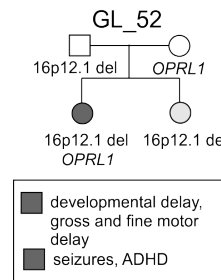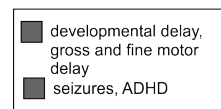

Supplement: S18 Fig — Pedigrees of 15 families with the 16p12.1 deletion, highlighting 23 genes with rare secondary likely-pathogenic mutations (CNVs and SNVs) that were identified in severely affected children with the 16p12.1 deletion. These 23 genes carrying “second-hit” mutations were selected for Drosophila experiments to test how their decreased expression affects the neurodevelopmental phenotypes observed for 16p12.1 homologs. Family members who carry either the 16p12.1 deletion or individual genes with “second-hits” are indicated in the pedigrees. Phenotypes observed for affected children and other family members are indicated below each pedigree. (PDF) [file pgen.1009112.s018.pdf]

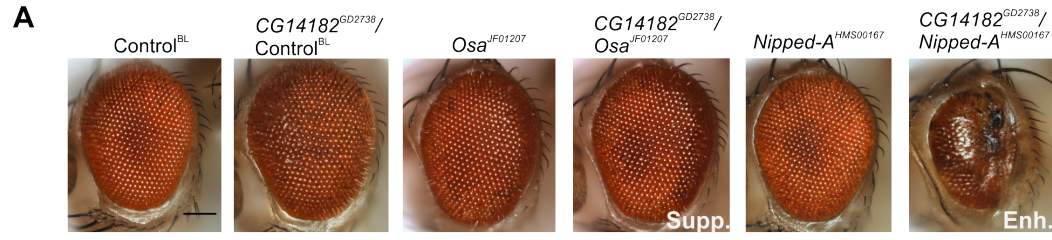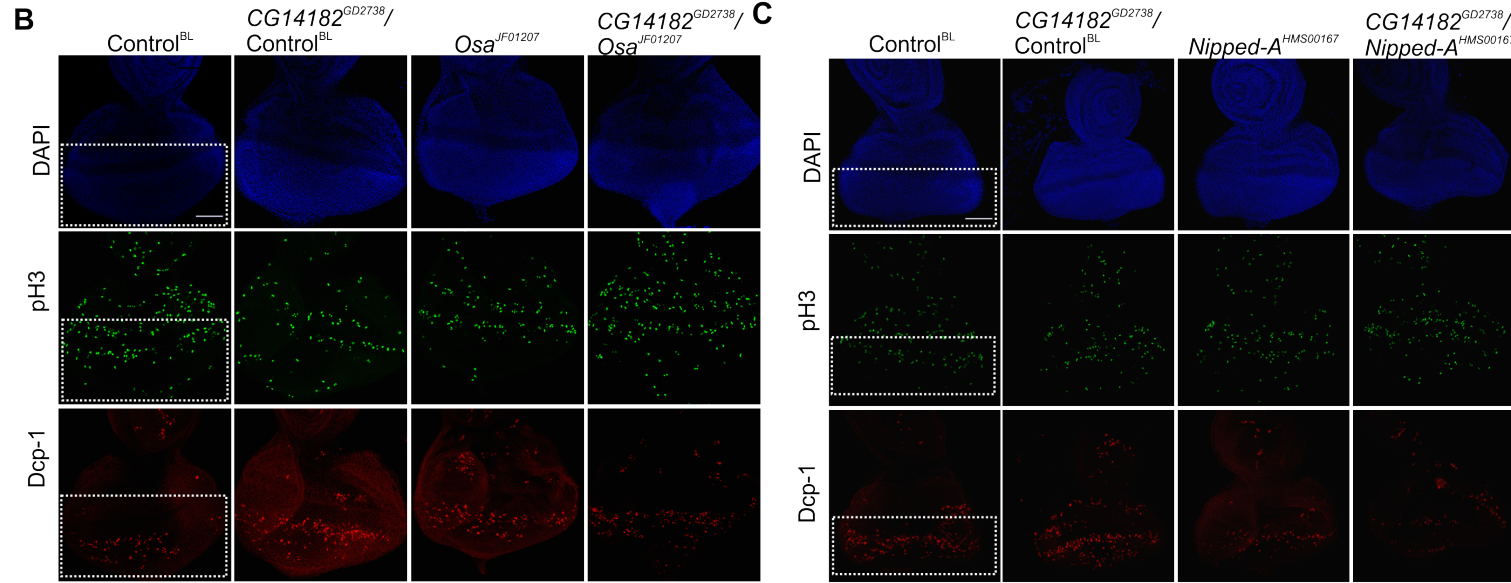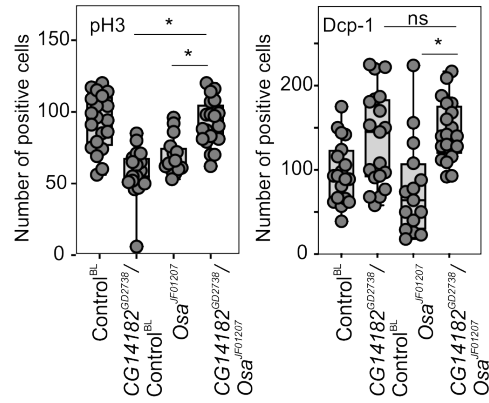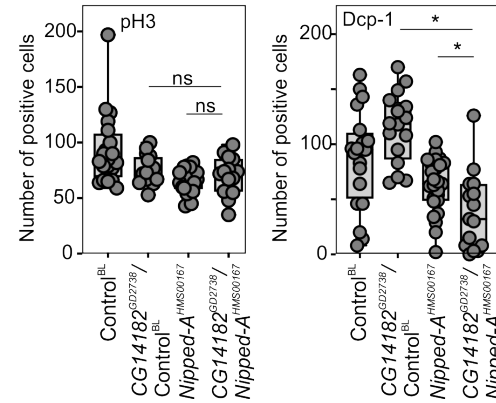

Supplement: S19 Fig — (A) Representative brightfield adult eye images show that simultaneous knockdown of CG14182GD2738 with osaJF01207 leads to a suppressed (Supp.) eye phenotype, while simultaneous knockdown of Nipped-AHMS00167 leads to synergistic enhancement (Enh.) in eye phenotype compared to single knockdown of CG14182GD2738. Scale bar represents 100 μm. (B) Representative confocal images of third instar larval eye discs stained with DAPI (blue) and anti-phosphorylated histone-3 (pH3, green) or anti-Dcp-1 (red), markers of cellular proliferation and apoptosis, respectively. Positive pH3 or Dcp-1 cells were quantified posterior to the morphogenetic furrow (indicated by white boxes). Simultaneous knockdown of CG14182GD2738 with osaJF01207 led to an increase in the number of pH3 positive cells (n = 20, two-tailed Mann-Whitney, *p = 2.33×10^−6^) compared to the single knockdown of CG14182GD2738. (C) Simultaneous knockdown of CG14182GD2738 with Nipped-AHMS00167 led to a significant reduction in the number of Dcp-1 positive cells compared to single knockdown of CG14182GD2738 (n = 15, in red, two-tailed, Mann-Whitney, *p = 2.54×10^−5^). Scale bars represent 50 μm. Boxplots represent all data points with median, 25th and 75th percentiles. Statistical details, including sample size, confidence intervals, and p-values, are provided in S6 File. A list of full genotypes for fly crosses used in these experiments is provided in S1 File. (PDF) [file pgen.1009112.s019.pdf]

**A**Forebrain/midbrain area *polr3e/setd5*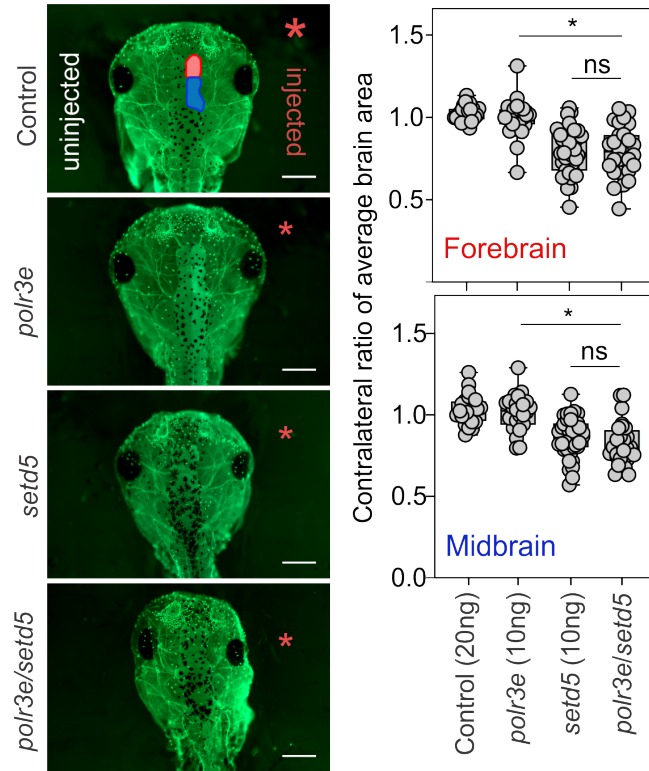**B**Axon length *mosmo/setd5*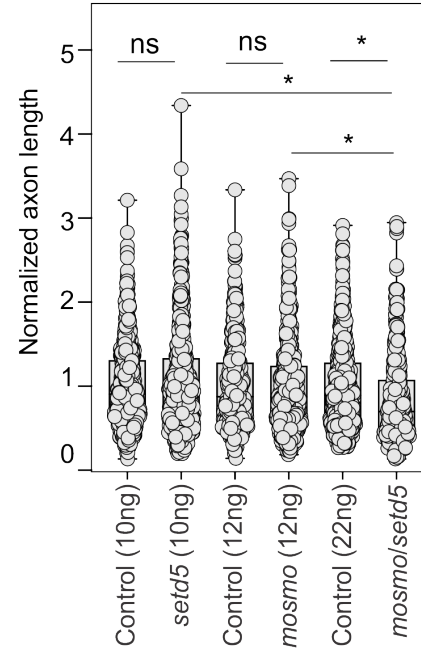**C**Axon length *polr3e/setd5*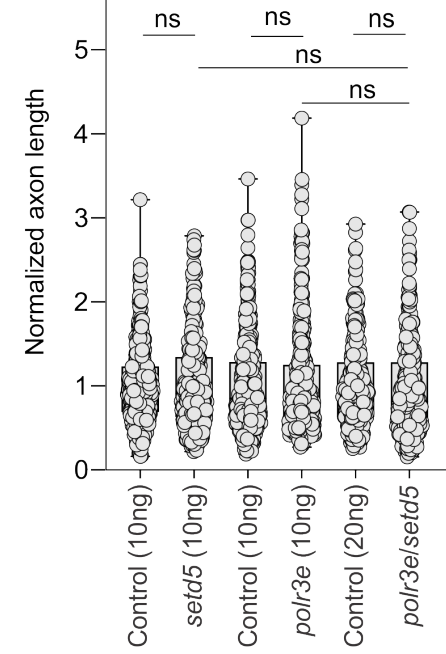

Supplement: S20 Fig — (A) Representative images stained with anti-tubulin show forebrain (red on control image) and midbrain (blue) areas of the side injected with morpholino (right, red asterisk), normalized to the uninjected side (left). Simultaneous knockdown of polr3e and setd5 in X. laevis led to decreased forebrain (n = 28, two-tailed student’s t-test, *p = 6.01×10^−7^) and midbrain area (*p = 1.67×10^−7^) compared to knockdown of polr3e alone, which were not different to the partial knockdown of setd5 alone (p>0.05). Scale bar represents 500 μm. (B) Normalized axon length of X. laevis tadpoles with simultaneous knockdown of mosmo and setd5 showed decreased axon length different from the control injected with 22ng of morpholino (n = 438, two-tailed, student’s t-test, *p = 2.95×10^−7^) and from individual knockdown of setd5 (*p = 1.86×10^−9^) or mosmo (*p = 3.34×10^−6^), showing a synergistic effect of decreased dosage of the homologs towards neuronal phenotypes. (C) Normalized axon length of X. laevis tadpoles with simultaneous knockdown of polr3e and setd5 showed no change in axon length (two-tailed student’s t-test, p>0.05). In each case, the individual knockdown was normalized and compared to the control injected with the same amount of morpholino. Boxplots represent all data points with median, 25th and 75th percentiles. Statistical details, including sample size, confidence intervals, and p-values, are provided in S6 File. (PDF) [file pgen.1009112.s020.pdf]

A

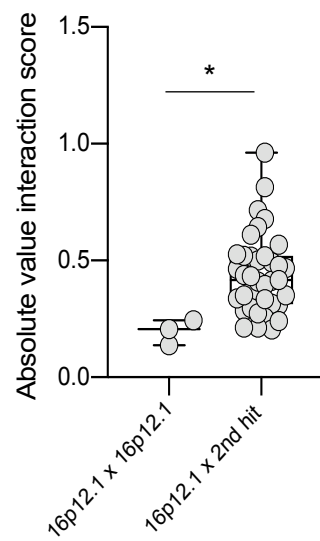

B

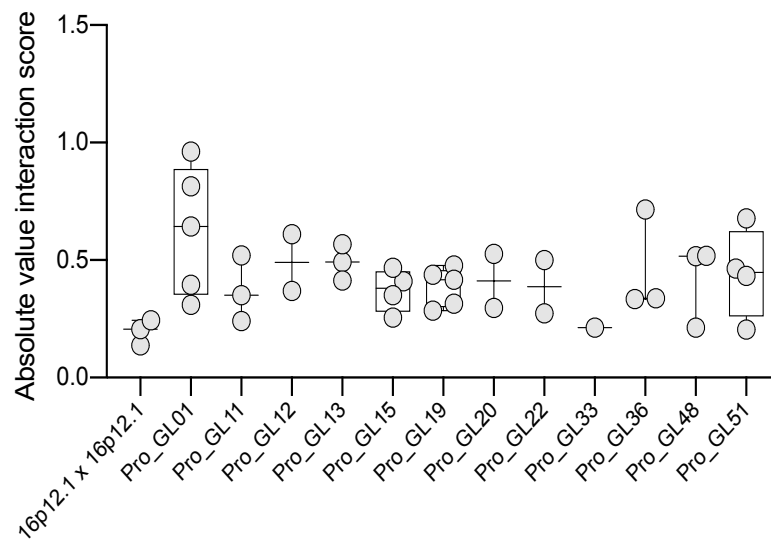

Supplement: S21 Fig — (A) Absolute values of scores of interactions identified between 16p12.1 homologs are lower compared to those identified between the 16p12.1 homologs and “second-hit” homologs (n = 3 for 16p12.1, n = 37 for interactions with “second-hits”, two-tailed Mann-Whitney, *p = 0.0032). (B) Comparison of absolute values of interaction scores between pairs of 16p12.1 homologs to those observed between 16p12.1 homologs and homologs of “second-hits” split by proband. Only one RNAi line per interacting gene is shown. (PDF) [file pgen.1009112.s021.pdf]

**A**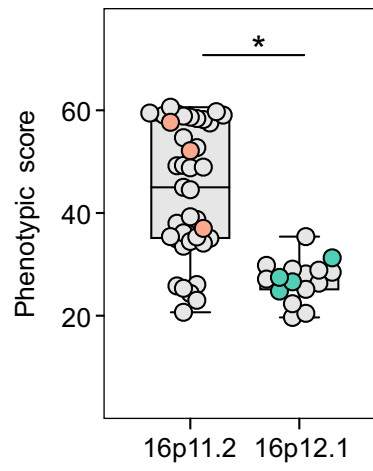**B**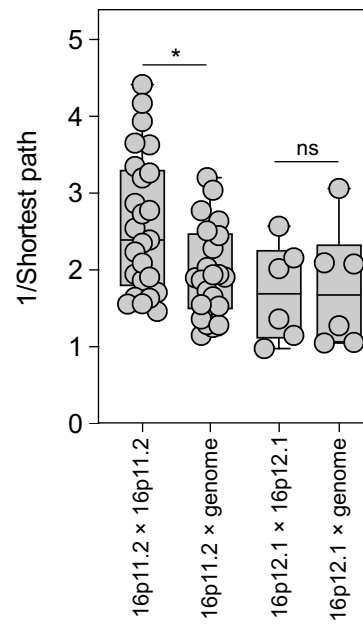

Supplement: S22 Fig — (A) Pairwise eye-specific knockdown of 16p11.2 homologs (rl, CG10465 and Pp4-19C crossed with other 16p11.2 homologs) lead to more severe phenotypic scores compared to pairwise knockdown of 16p12.1 homologs (n = 39 for 16p11.2, n = 16 for 16p12.1, one-tailed Mann-Whitney, *p = 3.51 ×10^−5^). Grey circles represent pairwise knockdowns, while 16p11.2 and 16p12.1 single-homolog knockdowns are represented in orange and green, respectively. (B) Analysis of a human brain-specific network shows higher average pairwise connectivity, measured as the inverse of the shortest path between two genes, between pairs of 16p11.2 genes compared to the connectivity of 16p11.2 genes to the rest of the genome (n = 25 two-tailed Mann-Whitney, *p = 6.64×10^−3^). This trend was not observed for 16p12.1 genes (n = 6, p>0.05). Boxplots represent all data points with median, 25th and 75th percentiles. Statistical details are provided in S6 File. (PDF) [file pgen.1009112.s022.pdf]

A

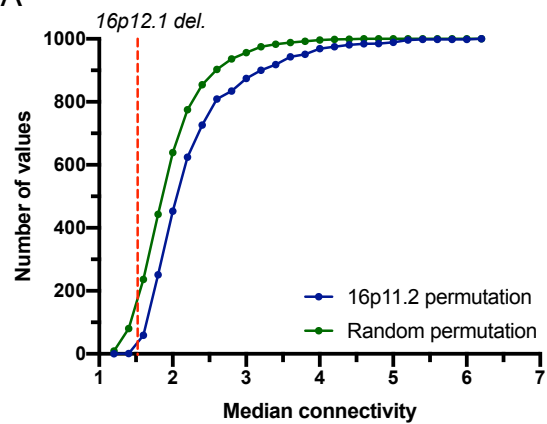

B

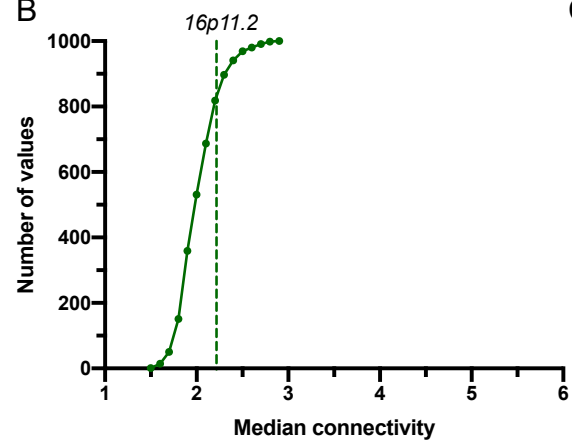

C

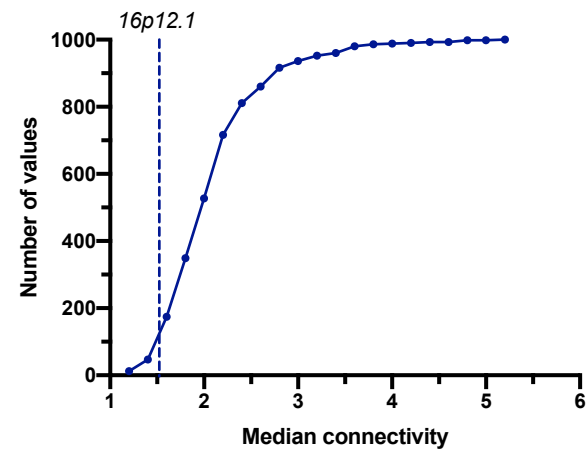

Supplement: S23 Fig — (A) Cumulative frequency plot shows low connectivity of 16p12.1 genes (observed value shown as red dotted line) in a human brain-specific interaction network when compared with 1000 permutations of six 16p11.2 genes (blue, 0.4th percentile, p = 0.117, one-tailed z-score test) and random sets of genes in the genome (green, 9.2nd percentile, p = 0.143, one-tailed z-score test). Cumulative frequency plots show 1000 simulations of bins of 25 contiguous genes (green) (B) or six contiguous genes (blue) (C), compared with the median connectivity values for 16p11.2 genes (green dotted line, 78.6th percentile, one-tailed z-score test, p = 0.246) and 16p12.1 genes (blue dotted line, 5.7th percentile, p = 0.130). (PDF) [file pgen.1009112.s023.pdf]
