# Supplementary material for: Functional assessment of the “two-hit” model for neurodevelopmental defects in Drosophila and X. laevis
Source: PLoS Genet. 2021 Apr 5;17(4):e1009112. doi: 10.1371/journal.pgen.1009112 (PMC8049494; doi:10.1371/journal.pgen.1009112)
Supplement: S4 Fig — (A) Clusters of enriched Gene Ontology (GO) Biological Process terms for differentially expressed fly genes observed with nervous system-specific knockdown of 16p12.1 homologs (left) and their human homologs (right). While some clusters of terms overlap among 16p12.1 homologs, genes dysregulated with knockdown of individual homologs exhibit unique enrichments for GO terms, suggesting their independent action towards neuronal development. Venn diagrams show overlaps of enriched GO Complete Biological Processes terms for (B) differentially-expressed fly genes observed with knockdown of individual 16p12.1 homologs, or (C) human homologs of the fly genes. We also observed that most of the (D) fly homologs or (E) human counterparts of the differentially-expressed genes were unique to each 16p12.1 homolog. A list of differentially expressed genes with knockdown of 16p12.1 homologs, as well as a list of all enriched GO terms for these gene sets, is detailed in S2 File. Venn diagrams were constructed using Venny 2.1 software (https://bioinfogp.cnb.csic.es/tools/venny). (PDF) [file pgen.1009112.s004.pdf]

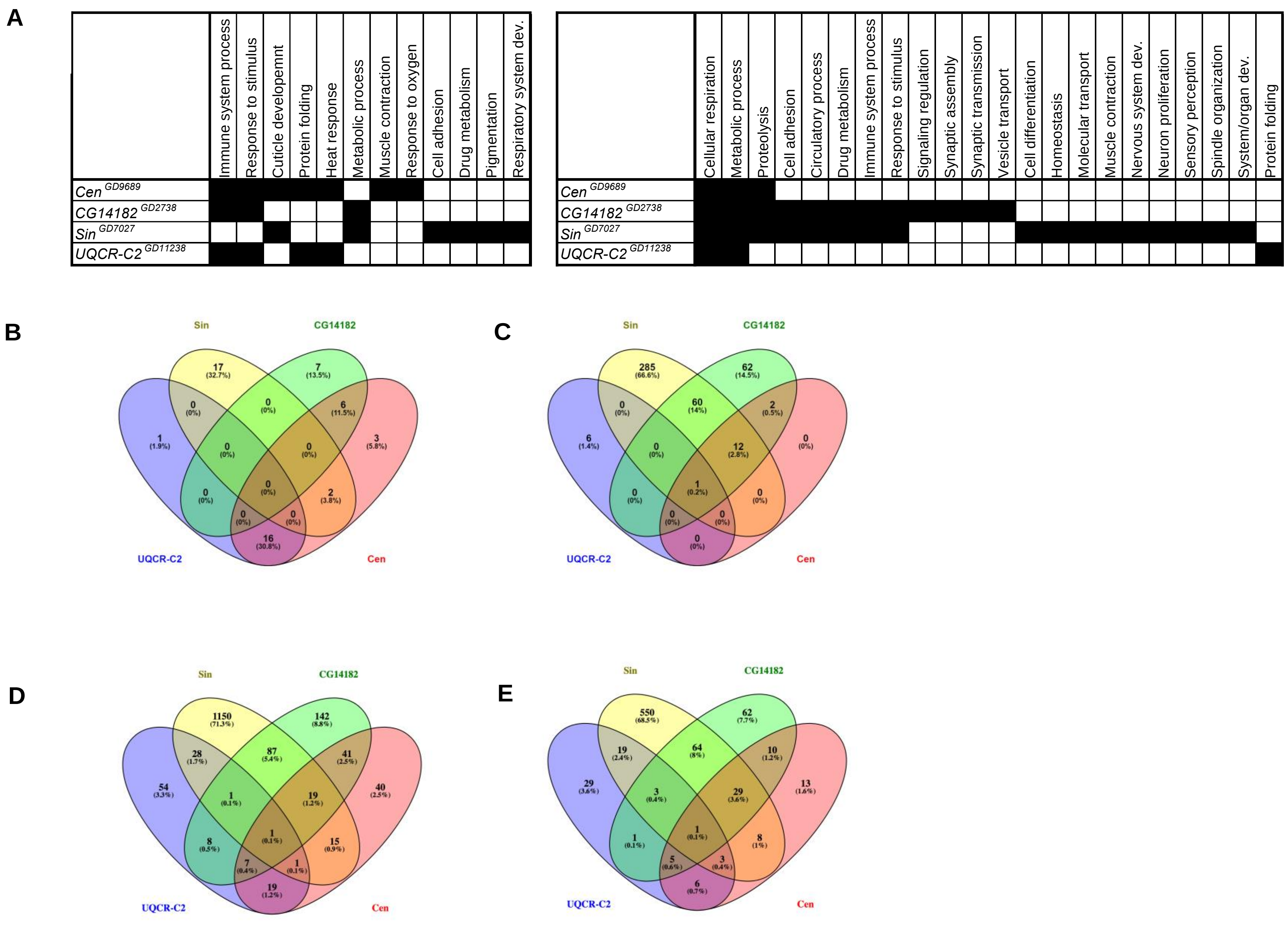
A
Immune system process
Response to stimulus
Cuticle developemnt
Protein folding
Heat response
Metabolic process
Muscle contraction
Response to oxygen
Cell adhesion
Drug metabolism
Pigmentation
Respiratory system dev.
Cen GD9689
CG14182 GD2738
Sin GD7027
UQCR-C2 GD11238
Cellular respiration
Metabolic process
Proteolysis
Cell adhesion
Circulatory process
Drug metabolism
Immune system process
Response to stimulus
Signaling regulation
Synaptic assembly
Synaptic transmission
Vesicle transport
Cell differentiation
Homeostasis
Molecular transport
Muscle contraction
Nervous system dev.
Neuron proliferation
Sensory perception
Spindle organization
System/organ dev.
Protein folding
B
C
D
E
Sin
CG14182
UQCR-C2
Cen
