# Supplementary material for: Functional assessment of the “two-hit” model for neurodevelopmental defects in Drosophila and X. laevis
Source: PLoS Genet. 2021 Apr 5;17(4):e1009112. doi: 10.1371/journal.pgen.1009112 (PMC8049494; doi:10.1371/journal.pgen.1009112)
Supplement: S1 Table — DIOPT [24] and reciprocal BLAST [82] searches were used to identify fly homologs of 16p12.1 genes. The expression of homologs in the larval central nervous system during development was assessed using FlyAtlas Anatomy microarray expression data from FlyBase [83]. (PDF) [file pgen.1009112.s024.pdf]

HGNC symbol	Organism	Homolog	% identity	DIOPT score	DIOPT rank	Larval central nervous system expression (FlyAtlas)
UQCRC2	*Drosophila*	*UQCR-C2*	31%	13.82	High	Moderate
CDR2	*Drosophila*	*Cen*	12%	8.93	High	Moderate
MOSMO	*Drosophila*	*CG14182*	51%	11.89	High	Low
POLR3E	*Drosophila*	*Sin*	32%	12.83	High	Moderate
EEF2K	*Drosophila*	NA	NA	NA	NA	NA
VWA3A	*Drosophila*	NA	NA	NA	NA	NA
PDZD9	*Drosophila*	NA	NA	NA	NA	NA
UQCRC2	*X. laevis*	*uqcrc2*	70%	---	---	---
CDR2	*X. laevis*	*cdr2*	63%	---	---	---
MOSMO	*X. laevis*	*mosmo*	82%	---	---	---
POLR3E	*X. laevis*	*polr3e*	66%	---	---	---
EEF2K	*X. laevis*	*eef2k*	72%	---	---	---
VWA3A	*X. laevis*	*vwa3a*	51%	---	---	---
PDZD9	*X. laevis*	NA	NA	---	---	---
