# Supplementary material for: Functional assessment of the “two-hit” model for neurodevelopmental defects in Drosophila and X. laevis
Source: PLoS Genet. 2021 Apr 5;17(4):e1009112. doi: 10.1371/journal.pgen.1009112 (PMC8049494; doi:10.1371/journal.pgen.1009112)
Supplement: S2 Table — This table lists the number of crosses, RNAi/mutant/overexpression lines, tested pairwise combination of genes, and genes that significantly enhanced or suppressed the phenotype of 16p12.1 homologs (S2A Table). The number of confirmed and potential negative and positive genetic interactions towards eye phenotypes using the multiplicative model is also provided (S2B Table). (PDF) [file pgen.1009112.s025.pdf]

Table S2A

Genes modulating phenotypes of 16p12.1 homologs[#]	Total	16p12.1 homologs	Patient-specific "second-hit" genes	Neurodevel/ functionally related genes	Transcriptome targets
Total number of crosses	521	39	227	119	136
RNAi/mutant/overexp lines	134	10	46	24	54
Genes (fly homologs)	80	4	24	13	39
Tested pairwise combinations of RNAi lines	388	30	181	95	82
Tested pairwise combinations of genes	224	12	96	55	61
Pairwise combinations enhancing phenotypes due to knockdown of 16p12.1 homologs	54	3	26	6	19
Pairwise combinations suppressing phenotypes due to knockdown of 16p12.1 homologs	29	1	6	12	10
Pairwise combinations that do not affect the phenotype of 16p12.1 homologs	76	3	32	17	24
Pairwise combinations whose effects on 16p12.1 homologs were not validated	65	5	32	20	8

Table S2B

Interactions*	Total	16p12.1 homologs	Patient-specific "second-hit" genes	Neurodevel/ functionally related genes	Transcriptome targets
Negative interactions (confirmed and potential)	41	0	6	10	25
Positive interactions (confirmed and potential)	63	3	31	12	17
No interaction (confirmed and potential)	61	2	30	18	11
Not validated interaction	55	7	29	12	7

[#]Genes modulating phenotypes of 16p12.1 homologs were confirmed using Mann-Whitney tests.
*Genetic interactions identified using the multiplicative model. Genes with no single knockdown *Flynotyper* phenotypes were excluded from multiplicative model testing.
