# Supplementary material for: Functional assessment of the “two-hit” model for neurodevelopmental defects in Drosophila and X. laevis
Source: PLoS Genet. 2021 Apr 5;17(4):e1009112. doi: 10.1371/journal.pgen.1009112 (PMC8049494; doi:10.1371/journal.pgen.1009112)
Supplement: S3 Table — Explanation of hypotheses, experimental design, results, and conclusions that can be drawn or hypotheses that can be further tested for each experiment performed in this study is provided. (PDF) [file pgen.1009112.s026.pdf]

**EXPERIMENT**	**Individual knockdown of 16p12.1 homologs**	**Pairwise knockdown of 16p12.1 homologs**	**Pairwise interactions of 16p12.1 homologs with "Neurodevelopmental genes"**	**Pairwise interactions of 16p12.1 homologs with "Transcriptome targets"**	**Pairwise interactions of 16p12.1 homologs with "Patient-specific second-hit genes"**	**Cellular phenotypes**
**HYPOTHESIS TESTED**	Multiple 16p12.1 homologs contribute to neurodevelopmental phenotypes	16p12.1 homologs interact towards neurodevelopmental phenotypes	16p12.1 homologs interact with genes within core neurodevelopmental pathways and functionally related genes towards neurodevelopmental phenotypes	16p12.1 homologs interact with their downstream genes towards neurodevelopmental phenotypes	Patient-specific “second-hits” can modulate neurodevelopmental phenotypes of 16p12.1 homologs through genetic interactions	Genes interact with 16p12.1 homologs towards neuronal defects by altering cellular phenotypes
**EXPERIMENTAL STRATEGY**	Individual knockdown of 16p12.1 homologs using tissue-specific drivers in *Drosophila* and whole-embryo knockdown in *X. laevis*, and assessment of multiple neuronal, cellular and developmental phenotypes	Simultaneous knockdown of 16p12.1 homologs - assessment of genetic interactions in the fly eye. Assessment of specific pairs of homologs towards brain and cellular phenotypes in *X. laevis*	Simultaneous knockdown of 16p12.1 homolog with neurodevelopmental genes and functionally related genes - assessment for genetic interactions in the fly eye	Simultaneous knockdown of 16p12.1 homolog with transcriptional targets or their functionally related genes - assessment for genetic interactions in the fly eye	Simultaneous knockdown of 16p12.1 homologs with homologs of patient-specific “second-hit” genes - assessment for genetic interactions in the fly eye	Assessment of cellular proliferation and apoptosis processes for validated two-hit interactions in the developing fly eye
			Genes in established neurodevelopmental pathways - 7 genes; Genes functionally related to 16p12.1 homologs - 6 genes	Up and down-regulated genes from transcriptome analysis of 16p12.1 homologs - 25 genes; Genes within enriched GO terms from transcriptome analysis - 14 genes	Homologs of “second-hit” genes from sequencing children with 16p12.1 deletion - 24 genes	
		12 combinations	55 combinations	61 combinations	96 combinations	3 combinations
**OUTCOME**	Global and homolog-specific phenotypes	3/12 positive interactions	22/55 interactions	42/61 interactions	37/96 interactions	Alterations of proliferation and apoptosis processes compared to single knockdown of 16p12.1 homolog

**EXPERIMENT**	**Individual knockdown of 16p12.1 homologs**	**Pairwise knockdown of 16p12.1 homologs**	**Pairwise interactions of 16p12.1 homologs with "Neurodevelopmental genes"**	**Pairwise interactions of 16p12.1 homologs with "Transcriptome targets"**	**Pairwise interactions of 16p12.1 homologs with "Patient-specific second-hit genes"**	**Cellular phenotypes**
**CONCLUSIONS**	Each 16p12.1 homolog contributes towards a range of developmental, neuronal, and cellular defects in flies/*X. laevis*	16p12.1 homologs contribute towards neurodevelopmental phenotypes through mild genetic interactions and additive effects	16p12.1 homologs interact with genes in core pathways towards neurodevelopmental phenotypes	Differentially expressed genes interact with 16p12.1 homologs towards neurodevelopmental phenotypes	Patient-specific second-hits can modify phenotypes of 16p12.1 homologs through synergistic and alleviating interactions	Interacting genes modulate proliferation and apoptosis phenotypes of 16p12.1 homologs during development
**HYPOTHESIS-GENERATING RESULTS**	Validation of phenotypes associated with each homologs in other model organisms	Assessment of identified interactions in specific neuronal cell types	Follow up of identified interactions (such as those involving homologs of *MOSMO* and *SETD5*) in other model organisms and human cell lines/organoids.			Assessment of mechanisms connecting genes and interactions to cellular defects in specific neuronal cell types
