# Supplementary material for: Functional assessment of the “two-hit” model for neurodevelopmental defects in Drosophila and X. laevis
Source: PLoS Genet. 2021 Apr 5;17(4):e1009112. doi: 10.1371/journal.pgen.1009112 (PMC8049494; doi:10.1371/journal.pgen.1009112)
Supplement: S4 Table — Genes carrying “second-hits” were previously identified [9] through exome sequencing and SNP microarrays in 15 children with the 16p12.1 deletion, and selected as disease-associated genes carrying rare (ExAC frequency ≤1%) copy-number variants, loss-of-function (frameshift, stopgain or splicing) mutations, or de novo or likely-pathogenic (Phred-like CADD ≥25) missense mutations. (PDF) [file pgen.1009112.s027.pdf]

Human gene	Chr.	Position	Ref.	Alt.	Variant Type	Family	Phenotype change of 16p12.1 homologs	Genetic interaction with 16p12.1 genes
SETD5	chr3	9488832	T	TAC	Frameshift insertion	GL_01	Enhancer *Sin*; Enhancer *CG14182*	Positive: *Cen* Negative: *CG14182*
LAMC3	chr9	133927967	C	T	Stopgain	GL_01	Enhancer *UQCR-C2*	Positive: *UQCR-C2* and *Sin*
DMD	chrX	33229421	C	T	Stopgain	GL_01	No change/Not validated	Positive: *Sin*
DPM1	chr20	49574926	C	T	Stopgain	GL_01	No change/Not validated	No interaction/Not validated
DNAH7	chr2	196759808	A	AT	Stopgain	GL_07	No change/Not validated	No interaction/Not validated
PDE11A	chr2	178879181	G	A	Stopgain	GL_07	No change/Not validated	No interaction/Not validated
CEP135	chr4	56877651	CAG	C	Frameshift deletion	GL_11	Suppressor *UQCR-C2*	Negative: *Cen* and *Sin*
NRXN1	chr2	50837494	NA	NA	Deletion	GL_11	Enhancer *UQCR-C2* and *Sin*	Positive: *CG14182*
PDLIM5	chr4	95575739	A	G	Nonsynonymous	GL_12	Enhancer *Cen*, *CG14182*	Positive: *UQCR-C2* and *Sin*
ARID1B	chr6	157099425	A	AGC	Frameshift insertion	GL_13	Suppressor *UQCR-C2*, *Cen*, *CG14182*, and *Sin*	Positive: *Cen* and *CG14182*
PEX1	chr7	92123885	GAGGAGCA	G	Frameshift deletion	GL_13	Enhancer *Sin*	Positive: *CG14182*
PYGM	chr11	64527223	G	A	Stopgain	GL_15	No change/Not validated	Potential positive *UQCR-C2*, *Cen*, *Sin*, *CG14182*
DST	chr6	56480833	G	A	Stopgain	GL_19	Enhancer *UQCR*-C2, *Cen*, *Sin*, and *CG14182*	Positive: *UQCR-C2*, *Cen*, *Sin*
PSMD1	chr2	231949783	T	A	Stopgain	GL_19	Enhancer *UQCR-C2*, *Sin*, and *CG14182*	Potential positive: *UQCR-C2*, *Sin*
RAPGEF6	chr5	130771676	G	A	Stopgain	GL_20	Enhancer *UQCR-C2*, *Cen*, *Sin*, and *CG14182*	Positive: *UQCR-C2*, *CG14182*
NALCN	chr13	101763037	G	C	Stopgain	GL_22	No change/Not validated	No interaction/Not validated
CADPS	chr3	62739381	C	T	Nonsynonymous	GL_22	Enhancer *Sin*	Positive: *UQCR-C2* and *Sin*
CAPN9	chr1	230895256	A	G	Splicing	GL_33	Enhancer *UQCR-C2* and *CG14182*	Potential positive: *Sin*

Human gene	Chr.	Position	Ref.	Alt.	Variant Type	Family	Phenotype change of 16p12.1 homologs	Genetic interaction with 16p12.1 genes
CHRNA7	chr15	30936285	NA	NA	Deletion	GL_36	Enhancer *Cen*	Potential negative *Cen*, *Sin*, *CG14182*
USP45	chr6	99891524	C	A	Stopgain	GL_46	No change/Not validated	Potential no interaction
PDE11A	chr2	178681632	CA	C	Frameshift deletion	GL_46	No change/Not validated	No interaction/Not validated
DNAH10	chr12	124289588	G	C	Splicing	GL_48	Enhancer *UQCR-C2* and *Sin*, Enhancer *CG14182*	Potential positive: *UQCR-C2*, *Cen*, *CG14182*
CACNA1A	chr19	13342531	G	A	Nonsynonymous	GL_51	Suppressor *CG14182*	Potential positive *UQCR-C2, Cen*, *Sin*, *CG14182*
OPRL1	chr20	62729293	GAC	G	Frameshift deletion	GL_52	No change/Not validated	No interaction/Not validated
