# Supplementary material for: Functional assessment of the “two-hit” model for neurodevelopmental defects in Drosophila and X. laevis
Source: PLoS Genet. 2021 Apr 5;17(4):e1009112. doi: 10.1371/journal.pgen.1009112 (PMC8049494; doi:10.1371/journal.pgen.1009112)
Supplement: S5 Table — Developmental and neuronal phenotypes observed with individual knockdown of 16p12.1 homologs in Drosophila and X. laevis. (PDF) [file pgen.1009112.s028.pdf]

Experiment		Knockdown of *Drosophila* homologs of 16p12.1 genes			
**Phenotype**	**Assay**	*UQCR-C2*	*Cen*	*Sin*	*CG14182*
Adult eye morphology	Eye phenotype (overexpression of *Dicer2*)	Normal	Normal	Rough eye	Rough eye
	Eye phenotype (no overexpression of *Dicer2*)	Normal	Normal	Moderate rough eye	Normal
Role in development	Ubiquitous knockdown	Larval lethal	Normal	Larval lethal	Normal
	Wing development	Lethal	Normal	Severe phenotype	Normal
Neuronal phenotypes	Lifespan	Increased	Normal	Reduced	Reduced
	Developmental timing	Normal	Normal	Delayed / larval lethality	Partial larval lethality
	Seizure susceptibility	Increased	Normal	Normal	Normal
	Complexity of dendritic arbors	Normal	Normal	Normal	Reduced
	Brain size	Normal	Normal	Reduced	Reduced
Cellular proliferation (developing brain)	pH3 staining	NA	NA	Reduced	Reduced
Apoptosis (developing brain)	Dcp-1 staining	NA	NA	Reduced	Normal
RNA sequencing (adult heads)	Differential gene expression (fly homologs)	Protein folding, heat shock	Protein folding, heat shock protein, muscle contraction	Cell adhesion, respiratory system development	No clear relevant functional enrichment
	Differential gene expression (human homologs)	Protein folding	Proteolysis	Muscle contraction, nervous system development, system/organ development	Synapse assembly and transmission, histone methyltransferase function, small nucleolar ribonuclear complex

**Experiment**		**Knockdown of *X. laevis* homologs of 16p12.1 genes**			
**Phenotype**	**Assay**	*uqcrc2*	*cdr2*	*polr3e*	*mosmo*
Craniofacial features	Face width	Normal	Decreased	Decreased	Decreased
	Face height	Normal	Increased	Normal	Increased
	Orofacial area	Normal	Normal	Decreased	Decreased
	Eye area	Normal	Decreased	Decreased	Decreased
	Face angle	Normal	Decreased	Decreased	Decreased
Brain phenotypes	Forebrain size (partial KD)	Normal	Normal	Normal	Reduced
	Midbrain size (partial KD)	Normal	Normal	Normal	Reduced
	Forebrain size (stronger KD)	Normal	Lethal	Reduced	Reduced
	Midbrain size (stronger KD)	Normal	Lethal	Reduced	Reduced
Axon outgrowth	Axon length (stronger KD)	Normal	Lethal	Normal	Decreased
Cellular proliferation (developing embryo)	pH3 staining - western blot	NA	NA	Reduced	Normal
