# Supplementary material for: Functional assessment of the “two-hit” model for neurodevelopmental defects in Drosophila and X. laevis
Source: PLoS Genet. 2021 Apr 5;17(4):e1009112. doi: 10.1371/journal.pgen.1009112 (PMC8049494; doi:10.1371/journal.pgen.1009112)
Supplement: S6 Table — (PDF) [file pgen.1009112.s029.pdf]

Organism	Human gene	Gene	Primer name	Primer sequence (5'-3')
D. melanogaster	*UQCRC2*	*UQCR-C2*	UQCR-C2_Fwd1	TCTGTCAAGGCTGTGAATGCC
D. melanogaster			UQCR-C2_Rev1	AAAACCGAACAGACCAGCGT
D. melanogaster	*CDR2*	*Cen*	Cen_Fwd1	GCAGACGGACAACTCCATCC
D. melanogaster			Cen_Rev1	TCACCATGGGAGAGCCATTC
D. melanogaster	*MOSMO*	*CG14182*	CG14182_Fwd	TCCCGACTGGATCATCACG
D. melanogaster			CG14182_Rev	AGTCCCAATCGGATGTCACC
D. melanogaster	*POLR3E*	*Sin*	Sin_Fwd1	AAACGTGGCATCATGGACAA
D. melanogaster			Sin_Rev1	GGTTATGGAACGCGAGCTTG
D. melanogaster	*RP49*	*Rp49*	rp49_Fwd	GCAAGCCCAAGGGTATCGA
D. melanogaster			rp49_Rev	ACCGATGTTGGGCATCAGA
X. laevis	*MOSMO*	*mosmo*	MOSMO_Fwd_L_S	CTTTGCCATCGCCAGTATCG
X. laevis			MOSMO_Rev_L	GGTAATTTGTAGGGTTGGCCTC
X. laevis			MOSMO_Rev_S	GGATGTTTGTTTCTGGCAGC
X. laevis	*UQCRC2*	*uqcrc2*	UQCRC2_Fwd_L	CCGTGGAATTGAAGCTGTTG
X. laevis			UQCRC2_Rev_L	TAATCCAACCAGTGCCATCC
X. laevis			UQCRC2_Fwd_S	ATTACTCGCCCTCATCCAAG
X. laevis			UQCRC2_Rev_S	CAGTACAAGGAGTTAGCCAGTG
X. laevis	*POLR3E*	*polr3e*	POLR3E_Fwd_L	GGAAAATGAAGATGACGATCC
X. laevis			POLR3E_Rev_L	GATGTGCCATCAACATTCAGG
X. laevis	*CDR2*	*cdr2*	CDR2_Fwd_L_S	GACAGCAACGTGGAGGAGTTC
X. laevis			CDR2_Rev_L_S	TGCTCATTCATCCGACGCAG
X. laevis	*SETD5*	*setd5*	SETD5_Fwd_L_S	ATCCCTCTGGGAGTCACCAC
X. laevis			SETD5_Rev_L_S	TGAGTGAATCCCATTGTGCTCTG
X. laevis	*ODC1*	*ODC1*	ODC1_Fwd	GCCATTGTGAAGACTCTCTCCATTC
X. laevis			ODC1_Rev	TTCGGGTGATTCCTTGCCAC
