# Supplementary material for: Functional assessment of the “two-hit” model for neurodevelopmental defects in Drosophila and X. laevis
Source: PLoS Genet. 2021 Apr 5;17(4):e1009112. doi: 10.1371/journal.pgen.1009112 (PMC8049494; doi:10.1371/journal.pgen.1009112)
Supplement: S7 Table — (PDF) [file pgen.1009112.s030.pdf]

**Morpholino target**	**Morpholino sequence**
mosmo L allele	5-ACAATTGACATCCACTTACTGCCGG-3
mosmo S allele	5- CACCTTCCCTACCCCGCTACTTAC-3
polr3e L allele	5-ACTGTAAGCCTCTTTTGCCTTACCT-3
uqcrc2 L allele	5-ACAGTGTCTCTAAAGCACAGATACA-3
uqcrc2 S allele	5-CCCCTAACCCATTAAACATATACCT-3
cdr2 S and L alleles	5-CATCCCTCCCATACACTCACCTTG-3
setd5 S and L alleles	5-TGATTCAGGGCTGTAGAGGAGAAAA-3
standard control morpholino	5-CCTCTTACCTCAGTTACAATTTATA-3
