# Supplementary material for: Functional assessment of the “two-hit” model for neurodevelopmental defects in Drosophila and X. laevis
Source: PLoS Genet. 2021 Apr 5;17(4):e1009112. doi: 10.1371/journal.pgen.1009112 (PMC8049494; doi:10.1371/journal.pgen.1009112)
Supplement: Attachment — Submitted filename: Rebuttal_Pizzo_etal_2020.docx [file pgen.1009112.s037.docx]

**RESPONSE TO EDITORS’ AND REVIEWERS’ COMMENTS**

We thank the reviewers for their comments on our manuscript. Please find below **a summary of new analyses and revisions**, followed by a **point-by-point response to reviewers’ comments**.

1. Assessment of interactions using a multiplicative model: We performed a more formal assessment of genetic interactions using a previously described multiplicative model. We find that this model is not only concordant with the interactions identified in the submitted version using ANOVA-based tests, but also identified additional interactions. The multiplicative model allowed us to distinguish combinatorial effects that can be due to genetic interactions from the expected additive effects in a rigorous manner. The overall results have not changed, but the clarity of our interpretations of results has significantly improved. We have updated the **Methods**, **Results**, and **Discussion,** provided new **Figure 3D**, revised **Figures 4, 5** and **6**, and revised **S2 Table**, **S3 File**, **S6 File**, **S9 Figure,** and **S17 Figure**.
2. Summary of overall hypotheses, experimental design, results, and conclusions of this work: We have revised text in the **Introduction**, **Results**, and **Discussion**, as well as included new **S10 Figure** and **S3 Table**, detailing the hypothesis driving our experiments and results and interpretations that are conclusive or hypothesis-generating.
3. Clarification of how categories of genes for interaction studies were selected: We have revised the **Results** to provide more details on how genes within established neurodevelopmental pathways, transcriptome targets, and patient-specific “second-hits” were chosen for interaction experiments. We have also included new **S2 Table** detailing the total number of genes studied, number of crosses, and pairwise gene interactions assessed.
4. Contextualizing interactions of 16p11.2 homologs compared to that of 16p12.1 homologs: We used the previously described metric called “*interaction scores*” to compare the magnitude of interactions between 16p11.2 homologs with those between 16p12.1 homologs. We have modified the **Methods**, **Results**, and **Discussion,** and included a new **Figure 7C** and a new **S4 File**.
5. Gene-focused analysis of gene ontology (GO) enriched categories: In addition to providing a list of enriched GO terms, we also present a gene-focused analysis of differentially expressed genes identified from RNA-sequencing experiments. We have modified the **Results** and **S4 Figure** to include these analyses.
6. Analysis of genes that do not have fly homologs, and contextualizing the observations in relation to fly results: We analyzed the connectivity of 16p12.1 genes (*EEF2K* and *VWA3A*) that do not have fly homologs within the context of a human brain-specific interaction network. We have modified the **Discussion** and provide connectivity data for these genes in **S5 File**.
7. General assessment of RNAi effects on eye phenotypes: We analyzed the phenotypes observed for all RNAi lines used in our study, to demonstrate that the use of RNAi lines does not always lead to eye phenotypes, and provide details in **Table** **R7**.
8. Clarification for selection of RNAi lines: We provide details in the **Results** and **Methods** on criteria used to select the RNAi lines of 16p12.1 homologs for main experiments and validations.
9. Modifications to figures to improve presentation: We have modified **Figure 2A** to include the *GAL4* lines used to assess the reported phenotypes; modified **Figure 2B** to better illustrate the control line, which was not clearly visible before; and modified all **main and supplemental figures** to accurately label background-specific controls.

**POINT-BY-POINT RESPONSE TO REVIEWER COMMENTS**

**REVIEWER 1**

“*This interesting and ambitious paper aims to assess interaction among genes of the 16p12.1 CNV with each other and with potential ‘second-hit’ candidate genes using Drosophila and Xenopus models. Although they present some intriguing data and hypotheses, substantial clarification could help the reader understand the scope, results, and interpretation of the study*”.

***Q1. ‘****The manuscript includes terms like ‘interaction’, ‘epistasis’, ‘additive’, and ‘synergistic’ that need to be defined and then used precisely and supported, e.g. test whether the combined effects are significantly different than an additive prediction. Currently, the distinctions among these terms are unclear and may be misused (by a statistical definition), such as ‘additive interaction’. Other examples include results that polr3e and mosmo pairwise knockdown do not show more extreme phenotypes than one gene alone, yet this is referred to as ‘additive’, which typically means the sum of the effects of both. If one of the genes had no effect to begin with, the conclusion should just be no interaction”*

**Authors’ response:** We thank the reviewer for this very important comment. Several methods have been proposed to assess genetic interactions, including ANOVA-based tests for linear/additive effects and a *multiplicative model* (*Clark et al, Genetics, 1997; Mackay et al, Nature Rev Genet, 2014*). To address the reviewer’s question, we applied the multiplicative model to perform a formal analysis of our two-hit experiments in the fly eye to assess whether the observed effects due to simultaneous knockdown of two genes deviated significantly from the expected prediction of no interaction or independent combined effects, i.e., “additive” effects, of individual genes (*Clark et al, Genetics, 1997; Mackay et al, Nature Rev Genet, 2014; Dixon et al, Annu. Rev. Genet., 2009*). According to the multiplicative model, the expected effect is calculated as the product of the phenotypes observed with the individual mutants/knockdowns (*Dixon et al., Annu. Rev. Genet., 2009*). Mani and colleagues compared methods for defining genetic interactions and concluded that the multiplicative model is better at identifying functional relationships than other methods (*Mani et al*., *PNAS*, 2008). In fact, this model has been applied to estimate the expected phenotypes for quantitative traits such as fitness, cell count, and protein folding in endoplasmic reticulum, and to identify genetic interactions in *E. coli*, yeast, and *Drosophila* (*Jonikas et al., Science, 2009; Elena et al., Nature 1997; Mani et al, PNAS, 2008; Baryshnikova et al., Nat. Methods., 2010; Horn et al. Nat Methods, 2011*). We present additional analyses here and in the revised manuscript to show the utility of the model **(**in **A**&**B)**, interpretation **(C**&**D)**, and concordance with ANOVA-based predictions **(E)**.

**(A) The multiplicative model for interactions of 16p12.1 homologs:** *First*, we normalized phenotype values from *Flynotyper* scores for each RNAi line (16p12.1 homolog and interacting second hits) and pairwise knockdowns to its background-specific control to obtain “percentage values compared to controls” (as in *Horn et al. Nat Methods, 2011; Baryshnikova et al., Nat. Methods., 2010)*. *Then*, we calculated expected phenotypic scores for all pairwise interactions of 16p12.1 homologs as the product of average scores from individual knockdowns, and compared the observed values with the expected scores for any significant deviation using a one-sample Wilcoxon signed-rank test with Benjamini-Hochberg correction. We have modified the **Methods** (except below) and added statistical details for this analysis **(S6 File)**.

***Materials and Methods, Page 32****: “The nature of genetic interactions between pairs of gene knockdowns was determined using a multiplicative model, which has been widely used for evaluating genetic interactions for quantitative phenotypes in yeast, Drosophila, and E. coli models (55-58, 98). This model tests whether the observed phenotypic effect of simultaneously knocking down two genes is different from the expected product of effects due to knockdown of individual genes…* *The magnitude of a genetic interaction was measured using “interaction scores”, calculated as the log2 ratio between the observed and expected values (S4 File)... Interactions assessed with only one fly line were considered as “potential negative”, “potential positive” or “potential no interaction” (S3 File).”*

**(B) Utility of multiplicative model for *Flynotyper* scores**: We tested the application of this model for assessing eye-specific interactions using *Flynotyper* scores (*Iyer et al, G3, 2016*). We found that all expected values calculated using *Flynotyper* scores from single knockdown lines were within the dynamic range of *Flynotyper* and overlapped with the observed values (**Figure R1**). We have included the distribution of the expected and observed phenotypic scores for single and pairwise knockdowns experiments in **S9 Figure** (also shown as **Figure R1**).

***Figure R1.*** *Range of observed and expected phenotypic scores using a multiplicative model. Histograms representing the distribution of observed (in grey) and expected (in blue) phenotypic scores of GMR-GAL4-mediated pairwise knockdowns of 16p12.1 homologs and interacting genes (top panel) and GMR-GAL4-mediated single knockdowns of potential interacting genes tested (bottom panel). The distribution shows an overlap of the observed and expected phenotypic scores values.*

**(C) Interpreting interactions of 16p12.1 homologs**: We applied the previously reported definitions that classified genetic interactions (i.e. deviation from “additive” or combined individual effects) as *positive* and *negative* (*Mani et al, PNAS, 2008; Baryshnikova et al., Nat. Methods., 2010; Jonikas et al., Science, 2009; Horn et al. Nat Methods, 2011; Dixon et al, Annu. Rev. Genet., 2009)*. *Positive* or alleviating genetic interactions were defined as those where the observed score from pairwise knockdown was significantly lower than the expected score, while *negative*, aggravating, or synergistic interactions were defined as those where the observed values was significantly higher than expected (**Figure R2**). *No interactions* were defined as pairwise effects with no significant difference between the observed and the expected value. We considered interactions to be “validated” when the results were confirmed in more than one fly line. When only one RNAi line was available for testing interactions, the outcomes were labeled as “potential positive”, “potential negative”, or “potential no interaction”. For example, the multiplicative model analysis showed that *Sin* and *CG14182,* fly homologs of *POLR3E* and *MOSMO*, respectively, exhibited a positive interaction with each other, validated using 2/3 RNAi lines. The results of analysis for interactions are in **S3 File (Table R1)**, along with a description of how the simultaneous knockdown modulated the phenotype of the 16p12.1 homolog, while a summary of the number of interactions identified for each functional category of genes is presented in **S2 Table (Table R2)**. Modified **Figure 3D** (**Figure R2**) uses an example to explain the multiplicative model, and modified **Figures 4-6** (**Figure R3-R5**) and **S17 Figure** (**Figure R6**) show interactions of 16p12.1 homologs using the multiplicative model (excerpts shown below):

(1) We have modified **Figure 3D** (shown below as **Figure R2**) to explain the expected results and hypothetical positive and negative interactions for homolog A and homolog B.

***Figure R2.*** ***Homologs of 16p12.1 genes contribute both additively and interactively towards neurodevelopmental defects. (D)*** *We applied a multiplicative model to identify the nature of genetic interactions for the pairwise knockdowns tested. The expected phenotype from simultaneous knockdown of homolog A and homolog B, or when the combined effect is additive indicating no genetic interaction (in blue), was calculated as the product of the normalized phenotypic scores (i.e. percentage of control) observed from knockdown of individual genes. Positive or alleviating genetic interactions were identified for combinations where the observed phenotype was significantly milder than expected (in green), while negative or aggravating interactions were identified when the combined phenotypes were significantly more severe than expected (in red). One-sample Wilcoxon signed rank tests with Benjamini-Hochberg correction for multiple testing were used to identify significant interactions.*

| (2) **Table R1. Summary of genetic interactions identified with 16p12.1 homologs in *Drosophila*#* (excerpt)** | | | | | | |
| --- | --- | --- | --- | --- | --- | --- |
| **Functional association** | **Human gene** | **Fly homolog** | ***UQCR-C2*** | ***Cen*** | ***CG14182*** | ***Sin*** |
| 16p12.1 homolog | *UQCRC2* | *UQCR-C2* | Not validated (1/2) | Not validated (1/2) | Not validated (1/2) | No interaction (2/2) |
| 16p12.1 homolog | *CDR2* | *Cen* | Positive (2/2) | Not validated (1/2) | Not validated (1/2) | Positive (2/2) |
| 16p12.1 homolog | *POLR3E* | *Sin* | Not validated (1/3) | No interaction (2/3) | Positive (2/3) | No interaction (2/3) |
| 16p12.1 homolog | *MOSMO* | *CG14182* | Not validated (1/2) | Not validated (1/2) | Not validated (1/2) | Not validated (1/2) |
| Homolog of "second-hit" gene | *SETD5* | *upSET* | No interaction (2/2) | Positive (2/2) | Negative (2/2) | No interaction (2/2) |
| Homolog of "second-hit" gene | *LAMC3* | *LanB2* | Positive (2/2) | Not validated (1/2) | Not validated (1/2) | Positive (2/2) |
| Homolog of "second-hit" gene | *DMD* | *Dys* | Not validated (1/2) | No interaction (2/2) | Not validated (1/2) | Positive (2/2) |

*numbers in parenthesis indicate number of lines with the result/number of lines tested

\# Representative rows from S3 File

(3) **Table R2A. Number of experiments performed, and genetic interactions identified in this study**

| **Genes modulating phenotypes of 16p12.1 homologs^#^** | **Total** | **16p12.1 homologs** | **Patient-specific "second-hit" genes** | **Neurodevel/ functionally related genes** | **Transcriptome targets** |
| --- | --- | --- | --- | --- | --- |
| Total number of crosses | 521 | 39 | 227 | 119 | 136 |
| RNAi/mutant/overexp lines | 134 | 10 | 46 | 24 | 54 |
| Genes (fly homologs) | 80 | 4 | 24 | 13 | 39 |
| Tested pairwise combinations of RNAi lines | 388 | 30 | 181 | 95 | 82 |
| Tested pairwise combinations of genes | 224 | 12 | 96 | 55 | 61 |
| Pairwise combinations enhancing phenotypes due to knockdown of 16p12.1 homologs | 54 | 3 | 26 | 6 | 19 |
| Pairwise combinations suppressing phenotypes due to knockdown of 16p12.1 homologs | 29 | 1 | 6 | 12 | 10 |
| Pairwise combinations that do not affect the phenotype of 16p12.1 homologs | 76 | 3 | 32 | 17 | 24 |
| Pairwise combinations whose effects on 16p12.1 homologs were not validated | 65 | 5 | 32 | 20 | 8 |
|  |  |  |  |  |  |
| **Table R2B: Types of genetic interactions of 16p12.1 homologs identified in this study** | | | | | |
| **Interactions*** | **Total** | **16p12.1 homologs** | **Patient-specific "second-hit" genes** | **Neurodevel/ functionally related genes** | **Transcriptome targets** |
| Negative interactions (confirmed and potential) | 41 | 0 | 6 | 10 | 25 |
| Positive interactions (confirmed and potential) | 63 | 3 | 31 | 12 | 17 |
| No interaction (confirmed and potential) | 61 | 2 | 30 | 18 | 11 |
| Not validated interaction | 55 | 7 | 29 | 12 | 7 |

^#^Genes modulating phenotypes of 16p12.1 homologs were confirmed using Mann-Whitney tests.

*Genetic interactions identified using the multiplicative model. Genes with no single knockdown *Flynotyper* phenotypes were excluded from multiplicative model testing.

**(4)** We have modified the **Results** to include the genetic interactions identified between 16p12.1 homologs (excerpt below).

***Results, page 12****: “In order to quantitatively assess whether the phenotypes observed with pairwise knockdowns could be attributed to either genetic interactions or expected additive effects, we applied a “multiplicative” model to the Flynotyper scores of pairwise knockdowns. The multiplicative model estimates the expected combined effect (i.e., no interaction) of two gene mutations as the product of the phenotypes observed with individual gene mutations (Fig 3D, S9 Fig), and identifies any deviation of the observed phenotypes from the expected values as positive (ameliorating the phenotype) or negative/synergistic (aggravating the phenotype) interactions...* *After applying the multiplicative model to our pairwise interaction data, we identified five pairwise combinations of 16p12.1 homologs that were validated using multiple RNAi lines (S2 Table). Two pairwise combinations corresponded with no interactions or additive effects, while the remaining three were positive genetic interactions, with an observed phenotype milder than expected (S3 File). Only one out of the four pairwise knockdowns that resulted in significant changes in eye severity compared with single hit recombinant lines crossed with control (S3 File) corresponded with a genetic interaction, while the rest were not validated across multiple fly lines tested.”*

**(5)** We modified the **Results** and **Figures** to include genetic interactions between homologs of 16p12.1 genes and neurodevelopmental genes, genes identified as transcriptomic targets, and homologs of patient-specific “second-hit” genes identified in families with 16p12.1 deletion. We have modified the **Results** in three locations (excerpts below), and revised **Figure 4** (shown below as **Figure R3**), **Figure 5D** (shown as **Figure R4**), **Figure 6A** (shown as **Figure R5**), and **S17 Figure** (shown as **Figure R6**) to present the new results.

***Results, page 15****: “Using Flynotyper to quantify adult eye defects and the multiplicative model to identify genetic interactions, we identified interactions specific to an individual 16p12.1 homolog or those involving multiple homologs (S11-S16 Fig, S3 File). For example, CG10465 (KCTD13) negatively interacted with UQCR-C2 and CG14182, leading to significantly more severe phenotypes than expected using the multiplicative model (Fig 4B). Similarly, simultaneous knockdown of Sin with kis led to an exaggerated eye phenotype, suggesting negative interactions between the genes (Fig 4B). Overall, we confirmed 22 interactions out of the 55 pairwise combinations (40%) tested, including both positive (12/55) and negative (10/55) effects (Fig 4C-D, S17 Fig, S2 Table).”*

***Results, page 15:*** *“Next, to correlate 16p12.1 homologs to developmental functions and pathways (S10 Fig, S3 Table), we screened for interactions of the homologs with 25 dysregulated fly genes selected from our transcriptome studies as well as 14 genes within enriched Gene Ontology categories, such as nervous system development and function (Dscam1, Asp, mGluR, NaCP60E), protein folding (Hsp23, Hsp26, Hsp70Ab), and muscle contraction (Actn, ck). We identified interactions for 42 out of 61 tested pairs (68.8%, S2 Table), validated using additional lines when available (S3 File)… Overall, we identified 64 pairwise interactions between the 16p12.1 homologs and genes from established neuronal functions and transcriptome targets (Fig 4C-D, S2 Table), further suggesting an involvement of these homologs in multiple neurodevelopmental pathways.”*

***Results, page 16****: “Out of the 96 combinations tested, we identified 32 pairwise knockdowns that modulated the phenotype of a 16p12.1 homolog, confirmed with additional lines when available, for 11 out of 15 families carrying “second-hit” genes (Fig 5A-C, S2-S3 Tables). In fact, the phenotypic effects of 16 out of 32 combinations were attributed to genetic interactions (Fig 4D, S3 File)… Furthermore, upSET, homolog of SETD5, led to enhancements of the phenotypes caused by knockdown of Sin and CG14182 (S3 File). Interestingly, while the phenotype caused by simultaneous knockdown of Sin and upSET was not different from expected using the multiplicative model, upSET synergistically interacted with CG14182, leading to an enhanced eye phenotype with pairwise knockdown (Fig 6A).”*

***Figure R3. Homologs of 16p12.1 genes show complex interactions with conserved neurodevelopmental genes and homologs of patient-specific “second-hit” genes.***

*(A) We evaluated how homologs of genes outside of the CNV region (Gene B), including genes carrying “second-hit” variants in children with the 16p12.1 deletion, genes within conserved neurodevelopmental pathways, and transcriptome targets, affect the phenotypes observed for homologs of 16p12.1 genes. We crossed eye-specific recombinant lines for each homolog with a total of 124 RNAi, mutant or overexpression lines for 76 interacting genes to test a total of 212 pairwise gene combinations. (B) Representative brightfield images of Drosophila adult eyes for recombinant lines of 16p12.1 homologs crossed with background-specific controls (ControlKK,, also represented as CKK) or RNAi lines for kis and CG10465, are shown as examples of genetic interactions between the 16p12.1 homologs and homologs of neurodevelopmental genes. Bar plots show normalized phenotypes (median ± interquartile range) for 16p12.1 recombinant lines crossed with background-specific control or with RNAi lines for interacting genes. SinGD7027 negatively interacted with kisKK100890 and led to a more severe phenotype (two-tail one-sample Wilcoxon signed rank test with Benjamini-Hochberg correction, n=11, *p=0.012, in red) than expected (in blue) under a multiplicative model. Similarly, CG10465KK105756 negatively interacted with UQCR-C2GD11238 (n=10, *p=0.024) and CG14182GD2738 (n=10, *p=0.015), leading to more severe eye phenotypes than expected. Phenotypes are represented as percentage of average, i.e. normalized to Flynotyper scores from control flies carrying the same genetic background as the interacting gene. Scale bar represents 100 μm. (C) Heatmaps show interaction scores calculated as the log2 ratio between the average of observed and expected phenotypic scores. Positive scores represent negative aggravating genetic interactions (in red), while negative scores represent positive alleviating interactions (in green). Grey boxes indicate pairwise crosses that were not tested or were not validated by multiple lines. A complete list of interaction scores is provided in S4 File. (D) Scatter plots depict interactions tested for 16p12.1 homologs. The plots show the average phenotypic score of the interacting gene on the x-axis, and the average observed phenotypic score for the pairwise knockdown on the y-axis. The blue line represents the expected phenotypic score of the pairwise knockdown calculated for each 16p12.1 homolog (value of first hit crossed with control, such as UQCR-C2GD11238 X ControlBL), and all possible phenotypic scores (ranging from 0 to 60) of the interacting genes are represented on the x-axis. All positive and negative (validated or potential) interactions are represented in green and red, respectively, and fly lines of genes with no significant interactions are shown in grey. Only lines from the BDSC stock center are represented here; S17 Fig. shows scatter plots representing VDRC stock lines.*

***Figure R4. Homologs of patient-specific “second-hits” modulate phenotypes of 16p12.1 homologs through additive and interactive effects. (D)*** *Bar plots show normalized phenotypes (median ± interquartile range) for the 16p12.1 recombinant lines crossed with background-specific controls (ControlGD or ControlBL, also represented as CGD, CBL, respectively) or with RNAi lines for Nrx-1GD14451_2, Cep135GD6121_2, Zasp52HMJ22168, shotHMJ23381, Rpn2HMS0533, Dhc98DMB03402, as examples of genetic interactions identified between the 16p12.1 homologs and homologs of patient-specific “second-hit” genes. SinGD7027 negatively interacted with Cep135GD6121_2 and led to a more aggravating phenotype (two-tail one-sample Wilcoxon signed rank test with Benjamini-Hochberg correction, n=10, *p=0.012, in red) than expected (in blue) under a multiplicative model, while other examples of pairwise knockdowns with homologs “second-hit” genes shown here led to positive genetic interactions (*p<0.05, in green). Details of number of homologs, fly lines and crosses, as well as a list of full genotypes for all interaction experiments are provided in S1 File. Statistical details, including sample size, confidence intervals, and p-values, are provided in S6 File.*

***Figure R5. Homolog of SETD5 synergistically interact with homologs of MOSMO to modify neurodevelopmental defects.*** *(A) Pedigree of a family with 16p12.1 deletion, with the proband also carrying a de novo pathogenic mutation in SETD5. Representative brightfield adult eye images for control and GMR-GAL4 knockdown of CG14182GD2738, upSETHMC03177, and CG14182GD2738/upSETHMC03177 are shown. Data show a negative genetic interaction with simultaneous knockdown of CG14182GD2738 and upSETHMC03177. Bar plots show normalized phenotypes (median ± interquartile range) for recombinant lines of CG14182GD2738 and SinGD7027 crossed with background-specific control (ControlBL, also represented as CBL) or upSETHMC03177. An aggravating phenotype is observed with CG14182GD2738/upSETHMC03177 (two-tailed one-sample Wilcoxon signed rank test with Benjamini-Hochberg correction, n=9, *p=0.018, in red) than expected (in blue).*

***Figure R6. Genetic interactions between 16p12.1 homologs and neurodevelopmental genes, transcriptome targets and homologs of patient-specific “second-hit” genes.*** *Scatter plots depict the interactions tested for GMR-GAL4 recombinant lines for UQCR-C2GD11238 (A), CenGD9689 (B), CG14182GD2738 (C), and SinGD7027 (D). The plots show the average phenotypic score of the interacting gene on the x-axis using VDRC GD (top) or KK (bottom) fly lines and the average observed phenotypic score for the double knockdown on the y-axis. Blue line represents the expected phenotypic score of the pairwise knockdown calculated as the product of the first hit phenotype (Flynotyper score of first hit crossed with control, such as UQCR-C2GD11238 X ControlGD) for each theoretical phenotypic value of interacting gene (ranging from 0 to 60) represented on x-axis. All positive and negative (validated or potential) interactions are represented in green and red, respectively, and fly lines of genes with no significant interactions are shown in grey.*

**(D) Quantifying the magnitude of interactions using *interaction scores*:** To quantify the magnitude of the interactions, we defined an “*interaction score*” as the log_2_ ratio between the observed and expected scores from the pairwise knockdowns, as previously reported (for example, *Horn et al. Nat Methods, 2011*). We have included a new **S4 File** (**Table R3**) detailing the observed values, expected values, and interaction scores (excerpt below), as well as a heatmap that shows all interaction scores for validated and potential interactions (**Figure R3C/Figure 4C**).

| **Table R3. Interaction scores and observed and expected normalized phenotypes* (excerpt)** | |
| --- | --- |
| **Double knockdown** | **Interaction score** |
| *UQCR-C2^GD11238^/Cen^GD9689^* | -0.24 |
| *UQCR-C2^GD11238^/Cen^GD01480^* | -0.17 |
| *UQCR-C2^GD11238^/CG14182^GD2738^* | 0.35 |
| *UQCR-C2^GD11238^/CG14182^GD2738_2^* | -0.22 |
| *UQCR-C2^GD11238^/Sin^GD7027^* | -0.14 |
| *UQCR-C2^GD11238^/Sin^HMC03807^* | -0.07 |

*****Representative rows from S4 File

**(E) Comparison of results from ANOVA-based tests and multiplicative model for classifying interactions:** Previous studies have used ANOVA as a statistical measure to identify deviations from the expected additive effects, conforming to a linear model (*Mackay et al, Nature Reviews Genetics, 2014; Clark and Wang, Genetics, 1997*). Our initial analysis was performed using ANOVA to identify genetic interactions. Here, we compared our initial analysis defined by ANOVA to test the linearity of combined independent effects with those defined by the multiplicative model to assess the concordance of interactions of 16p12.1 homologs. We found that 18 of the 21 interactions (85.7%) defined by ANOVA were also captured by the multiplicative model. Further, an additional 28 interactions were identified by the multiplicative model that were missed by ANOVA. A table of interactions identified from ANOVA and multiplicative model and their overlap is shown in **Table R4**.

**Table R4. Overlap between interactions tested using ANOVA and the multiplicative model.**

| **16p12.1 gene** | **Second-hit gene** | **ANOVA FDR (BH corr)** | **Significance** | **Multi-model FDR (BH corr.)** | **Significance** | **Concordance** |
| --- | --- | --- | --- | --- | --- | --- |
| *Cen^GD9689^* | *CG10465^KK105756^* | 0.852 | ns | 0.811 | ns | concordant |
| *Cen^GD9689^* | *CG30059^GD4893^* | 0.523 | ns | 0.03 | * | discordant |
| *Cen^GD9689^* | *Hsp23^Overexp Hsp23.K2^* | 0.970 | ns | 0.27 | ns | concordant |
| *Cen^GD9689^* | *Hsp26^Overexp Hsp26^* | 0.264 | ns | 0.045 | * | discordant |
| *Cen^GD9689^* | *nAChRα6^GD1045^* | 0.002 | * | 0.029 | * | concordant |
| *Cen^GD9689^* | *PDZ-GEF^HM05139^* | 0.040 | * | 0.03 | * | concordant |
| *Cen^GD9689^* | *PDZ-GEF^JF01765^* | 0.238 | ns | 1 | ns | concordant |
| *Cen^GD9689^* | *shot^GL01286^* | 0.767 | ns | 0.029 | * | discordant |
| *Cen^GD9689^* | *shot^HMJ23381^* | 0.134 | ns | 0.029 | * | discordant |
| *Cen^GD9689^* | *shot^JF02971^* | 0.852 | ns | 0.569 | ns | concordant |
| *Cen^GD9689^* | *Zasp52^HMJ22168^* | 0.767 | ns | 0.031 | * | discordant |
| *Cen^GD9689^* | *Zasp52^JF01133^* | 0.767 | ns | 0.054 | ns | concordant |
| *CG14182^GD2738^* | *aurB^GL00202^* | 6.73E-05 | * | 0.015 | * | concordant |
| *CG14182^GD2738^* | *CalpA^JF03391^* | 0.685 | ns | 0.478 | ns | concordant |
| *CG14182^GD2738^* | *CG10465^KK105756^* | 0.191 | ns | 0.015 | * | discordant |
| *CG14182^GD2738^* | *CG30059^GD4893^* | 0.910 | ns | 0.263 | ns | concordant |
| *CG14182^GD2738^* | *CG34195^KK103308^* | 1.65E-04 | * | 0.015 | * | concordant |
| *CG14182^GD2738^* | *Dhc98D^MB03402^* | 0.794 | ns | 0.01 | * | discordant |
| *CG14182^GD2738^* | *Nipped-A^HMS00167^* | 7.63E-05 | * | 0.015 | * | concordant |
| *CG14182^GD2738^* | *PDZ-GEF^HM05139^* | 0.001 | * | 0.015 | * | concordant |
| *CG14182^GD2738^* | *PDZ-GEF^JF01765^* | 0.685 | ns | 0.018 | * | discordant |
| *CG14182^GD2738^* | *Rpn2^HMS00533^* | 0.852 | ns | 0.466 | ns | concordant |
| **16p12.1 gene** | **Second-hit gene** | **ANOVA FDR (BH corr)** | **Significance** | **Multi-model FDR (BH corr.)** | **Significance** | **Concordance** |
| *CG14182^GD2738^* | *shot^GL01286^* | 0.025 | * | 0.008 | * | concordant |
| *CG14182^GD2738^* | *shot^HMJ23381^* | 0.685 | ns | 0.226 | ns | concordant |
| *CG14182^GD2738^* | *shot^JF02971^* | 0.546 | ns | 0.869 | ns | concordant |
| *CG14182^GD2738^* | *upSET^HMC03177^* | 4.75E-09 | * | 0.018 | * | concordant |
| *CG14182^GD2738^* | *upSET^HMJ23045^* | 0.059 | ns | 0.015 | * | discordant |
| *CG14182^GD2738^* | *Zasp52^HMJ22168^* | 0.014 | * | 0.079 | ns | discordant |
| *CG14182^GD2738^* | *Zasp52^JF01133^* | 0.194 | ns | 0.015 | * | discordant |
| *Sin^GD7027^* | *Actn^GD1354^* | 0.264 | ns | 0.022 | * | discordant |
| *Sin^GD7027^* | *Bace^GD5366^* | 0.040 | * | 0.012 | * | concordant |
| *Sin^GD7027^* | *Cadps^GD9502_1^* | 0.267 | ns | 0.012 | * | discordant |
| *Sin^GD7027^* | *Cadps^GD9502_2^* | 0.168 | ns | 0.012 | * | discordant |
| *Sin^GD7027^* | *CG10465^KK105756^* | 0.023 | * | 0.05 | ns | discordant |
| *Sin^GD7027^* | *CG34195^KK103308^* | 1.65E-04 | * | 0.012 | * | concordant |
| *Sin^GD7027^* | *ck^KK102922^* | 0.672 | ns | 0.09 | ns | concordant |
| *Sin^GD7027^* | *Dhc98D^MB03402^* | 0.040 | * | 0.05 | ns | discordant |
| *Sin^GD7027^* | *didum^GD1848^* | 4.18E-04 | * | 0.012 | * | concordant |
| *Sin^GD7027^* | *Dscam1^GD14362^* | 0.040 | * | 0.012 | * | concordant |
| *Sin^GD7027^* | *Dscam1^KK100296^* | 0.166 | ns | 0.012 | * | discordant |
| *Sin^GD7027^* | *Dscam4^HMC03277^* | 0.685 | ns | 0.012 | * | discordant |
| *Sin^GD7027^* | *Gat^KK107895^* | 9.58E-05 | * | 0.012 | * | concordant |
| *Sin^GD7027^* | *Myo10A^KK108171^* | 0.008 | * | 0.012 | * | concordant |
| *Sin^GD7027^* | *Nrx-1^GD14451_1^* | 0.725 | ns | 0.226 | ns | concordant |
| *Sin^GD7027^* | *Nrx-1^GD14451_2^* | 0.685 | ns | 0.278 | ns | concordant |
| *Sin^GD7027^* | *Nrx-1^GD2619^* | 0.685 | ns | 0.012 | * | discordant |
| *Sin^GD7027^* | *PDZ-GEF^HM05139^* | 0.134 | ns | 0.05 | ns | concordant |
| *Sin^GD7027^* | *PDZ-GEF^JF01765^* | 0.519 | ns | 0.012 | * | discordant |
| *Sin^GD7027^* | *Pex1^HM05190^* | 0.910 | ns | 0.158 | ns | concordant |
| *Sin^GD7027^* | *Pex1^HMC03252^* | 0.304 | ns | 0.237 | ns | concordant |
| *Sin^GD7027^* | *Pten^GD13500^* | 0.615 | ns | 0.494 | ns | concordant |
| *Sin^GD7027^* | *Pten^KK109278^* | 0.040 | * | 0.012 | * | concordant |
| *Sin^GD7027^* | *rl^GD12910_1^* | 0.719 | ns | 0.356 | ns | concordant |
| *Sin^GD7027^* | *rl^GD12910_2^* | 0.794 | ns | 0.198 | ns | concordant |
| *Sin^GD7027^* | *Rpn2^HMS00533^* | 0.146 | ns | 0.012 | * | discordant |
| **16p12.1 gene** | **Second-hit gene** | **ANOVA FDR (BH corr)** | **Significance** | **Multi-model FDR (BH corr.)** | **Significance** | **Concordance** |
| *Sin^GD7027^* | *shot^GL01286^* | 0.038 | * | 0.012 | * | concordant |
| *Sin^GD7027^* | *shot^HMJ23381^* | 0.479 | ns | 0.012 | * | discordant |
| *Sin^GD7027^* | *shot^JF02971^* | 0.804 | ns | 0.343 | ns | concordant |
| *Sin^GD7027^* | *upSET^HMC03177^* | 0.848 | ns | 0.788 | ns | concordant |
| *Sin^GD7027^* | *upSET^HMJ23045^* | 0.589 | ns | 0.496 | ns | concordant |
| *Sin^GD7027^* | *Wnt2^JF03377^* | 0.246 | ns | 0.013 | * | discordant |
| *UQCR-C2^GD11238^* | *CalpA^JF03391^* | 0.675 | ns | 0.336 | ns | concordant |
| *UQCR-C2^GD11238^* | *CG10465^KK105756^* | 0.025 | * | 0.024 | * | concordant |
| *UQCR-C2^GD11238^* | *Dhc98D^MB03402^* | 0.019 | * | 0.024 | * | concordant |
| *UQCR-C2^GD11238^* | *Hsp23^Overexp Hsp23.K2^* | 0.852 | ns | 0.116 | ns | concordant |
| *UQCR-C2^GD11238^* | *LanB2^HMC04076^* | 0.255 | ns | 0.024 | * | discordant |
| *UQCR-C2^GD11238^* | *LanB2^HMJ23628^* | 0.804 | ns | 0.024 | * | discordant |
| *UQCR-C2^GD11238^* | *Nrx-1^GD14451_1^* | 0.675 | ns | 0.043 | * | discordant |
| *UQCR-C2^GD11238^* | *Nrx-1^GD14451_2^* | 0.912 | ns | 0.596 | ns | concordant |
| *UQCR-C2^GD11238^* | *Nrx-1^GD2619^* | 0.721 | ns | 0.051 | ns | concordant |
| *UQCR-C2^GD11238^* | *PDZ-GEF^HM05139^* | 0.134 | ns | 0.024 | * | discordant |
| *UQCR-C2^GD11238^* | *PDZ-GEF^JF01765^* | 0.685 | ns | 0.024 | * | discordant |
| *UQCR-C2^GD11238^* | *Rpn2^HMS00533^* | 0.403 | ns | 0.024 | * | discordant |
| *UQCR-C2^GD11238^* | *shot^GL01286^* | 0.194 | ns | 0.019 | * | discordant |
| *UQCR-C2^GD11238^* | *shot^HMJ23381^* | 0.852 | ns | 0.239 | ns | concordant |
| *UQCR-C2^GD11238^* | *shot^JF02971^* | 0.675 | ns | 0.033 | * | discordant |

***Q2.*** *“In the abstract, the authors set up a comparison with the 16p11.2 CNV, hypothesizing that its higher de novo frequency is associated with greater within-CNV interaction compared to 16p12.1 with greater inherited frequency and second-hit interaction. However, the authors do not present the 16p11.2 data for clear comparison in the results, so this major point in the abstract seems lost in the paper itself”.*

**Authors’ response:** To address the reviewer comments, we have now included in the **Results** section a comparison of genetic interactions between 16p12.1 homologs with that between 16p11.2 homologs. Using the multiplicative model, we found a higher magnitude of interactions between 16p11.2 homologs compared to that of 16p12.1 homologs, in line with our hypothesis that fewer and milder interactions among genes within the 16p12.1 deletion could correspond with the higher inheritance rates and the broader phenotypic variability of the deletion.

**(1)** We have modified the text in **Results**, in order to contextualize the relative magnitude of interaction scores observed for 16p12.1 homologs to those of 16p11.2 homologs, which we find to exhibit pervasive interactions with each other (*Iyer et al, Nature Comm, 2018*). Comparisons of the pairwise phenotypic effects among homologs of 16p11.2 or 16p12.1 genes, overlap of differentially expressed genes from RNA-sequencing experiments, and connectivity analyses of the two CNV regions in a human brain-specific interaction network are further expanded in the **Discussion**.

***Results, page 13:*** *“To contextualize these observations, we compared the strength of genetic interactions among the 16p12.1 homologs to those of homologs of genes affected by the autism-associated 16p11.2 deletion, a region with reported pervasive genetic interactions (20). We quantified the magnitude of genetic interactions using “interaction scores”, defined as the log2 ratio between the observed and expected phenotypic values from the multiplicative model (see Methods), and found significantly lower interaction scores for the 16p12.1 homologs compared to the 16p11.2 homologs (S4 File).”*

**(2)** We have included and cited **Figure 7C** (show below as **Figure R7**) in the **Discussion** section, to compare the nature of interactions between 16p11.2 homologs with those of 16p12.1 homologs.

***Figure R7. Functional relatedness of genes within disease-associated CNV regions correspond with higher pathogenicity.*** *(C) Pairwise knockdown of homologs of 16p11.2 genes (n=27) show a larger magnitude of interactions compared with those among 16p12.1 homologs (n=5, two-tailed Mann-Whitney test, *p=0.011). Interaction values of zero (blue shade) represent no interactions or additive effects, while values above or below zero represent negative (in red) and positive (in green) interactions, respectively.*

**(3)** We have modified the **Discussion** to include analysis of interactions using the multiplicative model (excerpt shown below).

***Discussion, page 22****: “In fact, using a multiplicative model, we found that the magnitude of interactions between homologs of 16p11.2 genes was stronger than that observed between 16p12.1 homologs (Fig 7C).”*

***Q3****. “Further, no context or negative control is given when reporting the number of interactions detected, e.g. are 18/55 and 29/61 unusual? What is the basis for comparison?”*

**Authors’ response**: We thank the reviewer for requesting clarification on this point. We performed genetic interaction studies to assess mechanistic connections of 16p12.1 homologs with genes in established neurodevelopmental pathways and those with direct functional relationships (55 interactions), as well as downstream transcriptomic targets of 16p12.1 homologs (61 interactions). Additionally, we assessed genetic interactions between 16p12.1 homologs with homologs of “second-hits” identified from patient studies (96 interactions).

We expected to identify a larger number of interactions with direct transcriptome targets compared to neurodevelopmental genes due to their potential direct functional relationships. In line with our hypothesis, our new analyses with the multiplicative model identified genetic interactions for 42/61 pairwise combinations (68.8%) with direct transcriptome targets, compared to 22/55 (40%) interactions identified with neurodevelopmental genes (p=0.0027, Fisher’s exact test). We also hypothesized that 16p12.1 homologs present higher numbers of genetic interactions with differentially expressed genes and functionally related genes compared with homologs of genes that carry “second-hits” in patients with the 16p12.1 deletion. We indeed detected a higher frequency of negative or positive interactions with transcriptome targets and neurodevelopmental or functionally-related genes (64/101) compared to “second-hit” genes (37/96, 38.5%, p=0.019, Fisher’s exact test) which may not be directly functionally connected to 16p12.1 homologs.

We included **S10 Figure** illustrating our hypotheses regarding the genetic interactions between 16p12.1 homologs and the different categories of interacting genes (shown below as **Figure R8**).

***Figure R8. Expected genetic interactions for each category of interacting gene analyzed in this study.*** *The diagram shows representative gene networks of each of the 16p12.1 homologs analyzed. UQCR-C2 is shown as an example, where the gene is closely connected to transcriptome targets and functionally related genes, while neurodevelopmental genes and patient-specific “second-hit” genes distribute more randomly between gene categories that can lead to positive and negative genetic interactions (top bubble) or additive effects (bottom bubble). These hypotheses aligned with our results, as we identified genetic interactions for 42/61 pairwise combinations (68.8%) with direct transcriptome targets, compared to 22/55 (40%) interactions identified with functionally related and neurodevelopmental genes (p=0.0027, Fisher’s exact test). Moreover, we observed that homologs of genes carrying “second-hits” in severely affected children with the 16p12.1 deletion interacted with 16p12.1 homologs in 37/96 (38.5%) of the pairs, although the proportion of genetic interactions was not as high compared to that identified with functionally related genes or genes in neurodevelopmental pathways and transcriptome targets (64/101, 63%, Fisher’s exact test, p=0.019).*

***Q4.*** *“Only four out of seven genes in the region can be studied in these model organisms. How does that impact the conclusions made about interactions within the region, etc., when >40% of the genes are not assayed at all?”*

**Authors’ response**: We agree with the reviewer that 16p12.1 genes without fly homologs, including *EEF2K*, *PDZD9* and *VWA3A,* may also contribute to the observed genetic complexity of the deletion, and further functional analyses in more sophisticated models are necessary to understand the effects of all genes in the deleted region. Besides *EEF2K*, little functional information is available for the other two genes that do not have fly homologs. Even though fly models cannot be studied for these genes, we were able to study the connectivity of *EEF2K* and *VWA3A* in a human brain-specific interaction network (*Greene et al., Nat Genet. 2015; Krishnan et al., Nat Neurosci 2016*). We found that *EEF2K* (percentile=29.4) and *VWA3A* (percentile=5.2) showed low average connectivity with other 16p12.1 genes compared with all pairwise interactions of genes in the network (**S5 File/Table R5**). This suggests that our observation that 16p12.1 genes generally act independently towards developmental phenotypes is also applicable to genes without fly homologs. More generally, we believe that our analysis of conserved fly homologs of 16p12.1 genes provides several important findings, such as neuronal and developmental effects of individual homologs, combined phenotypic effects and interactions with each other and with functionally-related and patient-specific hits, and altered cellular mechanisms that can be followed up in more sophisticated models. In fact, our previous work on the role of individual conserved homologs and interactions within the 16p11.2 deletion in *Drosophila* models (*Iyer et al., Nat Comm. 2018*) have been functionally and thematically replicated in both mouse and zebrafish studies (*McCammon et al, HMG, 2016; Qiu et al, Cell Rep, 2019*). We have included the following clarification on this point in the **Discussion** section of the manuscript:

***Discussion, page 20****: “Beyond the four conserved homologs evaluated in this study, little functional information is available on the other genes in the region, including VWA3A and PDZD9 as well as non-protein coding genes. Results from mouse models of EEF2K, which encodes a kinase associated with protein synthesis elongation, have postulated associations of this gene with synaptic plasticity (67), learning and memory (68), atherosclerosis-mediated cardiovascular disease (69), and depression (70). Although EEF2K could function in concert with the tested 16p12.1 genes to contribute towards neurodevelopmental features, it showed low connectivity (29th percentile) to other 16p12.1 genes in a human brain-specific interaction network compared to the pairwise connectivity of all genes in the network (71, 72) (S5 File). VWA3A showed even lower connectivity (5th percentile) to other 16p12.1 genes compared to all gene pairs, suggesting that 16p12.1 genes with no fly homologs are likely to show reduced network connectivity in the human brain. Further functional analyses that includes all protein-coding and non-coding genes are necessary for a comprehensive understanding of the consequences of the entire deleted region, as these genes may also contribute towards the pathogenicity of 16p12.1 deletion.”*

We have included the percentile of network connectivity values of *EEF2K* and *VWA3A* in **S5 File** (**Table R5**) shown below:

**Table R5. Connectivity values of *EEF2K* and *VWA3A* in a human brain-specific network**

|  | *VWA3A* connectivity | *EEF2K* connectivity |
| --- | --- | --- |
| *MOSMO* | 0.9 | 1.2 |
| *CDR2* | 1.2 | 1.7 |
| *EEF2K* | 1.0 | NA |
| *POLR3E* | 1.3 | 1.9 |
| *UQCRC2* | 1.5 | 2.4 |
| *VWA3A* | NA | 1.0 |
| Average against all 16p12.1 genes | 1.2 | 1.6 |
| Average against all genes in the genome | 1.3 | 1.6 |
| Percentile against all 16p12.1 genes | 5.2 | 29.4 |
| Percentile against all genes in the genome | 12.0 | 28.8 |

***Q5.*** *“How do the authors interpret the severity of some single-gene partial knockdowns under their hypothesis of needing second hits’? Two of the four genes tested show larval lethality with 40-60% expression, which is similar to human heterozygous levels. This doesn’t seem to square with being a good model to assess effects so modest they require interaction to have a phenotype in humans.”*

**Authors’ response:** We would like to address this conceptual question with the following points:
**(1)** The goal of our study was not to fully recapitulate human disease in a model organism, but to use the powerful genetic system of *Drosophila* to identify patterns of interactions and conserved mechanisms of 16p12.1 homologs. As in other models, the severity of features observed in fly models may be comparable to those in humans, but they do not recapitulate the exact features observed in humans. *For example*, genes that have prenatal lethality as a major heterozygous loss-of-function phenotype in mouse are more likely to show haploinsufficiency in humans (*Huang et al, PLOS Genet, 2010*). Similarly, essential genes in mouse are more likely to harbor deleterious autism-associated mutations than non-essential genes (*Ji et al, PNAS, 2016*). Furthermore, conserved genes such as *CNTNAP2*, *ATP2A2*, *DPM1*, and *CREBBP* are lethal when disrupted in flies, but human patients with loss-of-function mutations may only manifest neuropsychiatric features (*Oortveld et al., PLOS Genet, 2013*).

**(2)** Tissue-specific drivers in flies allow for testing a wide range of effects due to altered dosage and genetic interactions without compromising organism viability, as we observed with ubiquitous knockdown of *UQCR-C2* and *Sin*. Previous studies have used these drivers to assess genetic interactions among homologs of genes affected in multigenic disorders towards different phenotypes (*Grice et al., PLOS Genet, 2015; Grossman et al., PLOS Genet, 2011*). In particular, the fly eye has been a standard tissue to screen for genetic interactions in *Drosophila*, and multiple genetic modifiers have been identified using this system (*Branco et al., Hum Mol Genet, 2008; Cziko et al., Genetics, 2009*). As the 16p12.1 homologs only exhibited modest eye phenotypes (1/4 genes with significant eye roughness), we took advantage of the *Flynotyper* scores as a quantitative trait with high sensitivity and a wide dynamic range to assess for negative and positive genetic interactions. In fact, we observed an eye phenotype for only a subset of the 16p12.1 homologs when simultaneously knocked down with another gene, including *UQCR-C2* with *CG10465* (fly homolog of *KCTD13*), *Cen* with *CG30059* (homolog of *GNS*), and *CG14182* with *Nipped-A* (homolog of *TRRAP*), of which some of these changes were further characterized as genetic interactions under the multiplicative model.

Overall, we agree with the reviewer that there are limitations with using flies as a model for testing pairwise interactions, but we believe that the advantages conferred by the model provide interesting insights that we can follow-up in more detail and in other model organisms.

We have modified the **Results** section to clarify the use of the eye as a system to identify genetic interactions of the 16p12.1 homologs (excerpt below):

***Results, page 12****: “As GMR-GAL4-mediated knockdown of the 16p12.1 homologs only exhibited modest eye phenotypes, we next took advantage of Flynotyper scores as a quantitative trait with high sensitivity and a wide dynamic range to assess for genetic interactions among the 16p12.1 homologs.”*

***Q6.*** *Similarly, the authors report increased lifespan with one of the knockdowns and compare with other previously reported genes, with no explanation of the meaning of this comparison or specific genes showing the same phenotype. Likewise, craniofacial defects are noted in the Xenopus, but not interpreted with respect to the human phenotype associated with this CNV.”*

**Authors’ response:** We would like to respond to the reviewer’s comments in two parts:
**(A)** The reviewer is referring to an interesting observation regarding the increased lifespan observed with *UQCR-C2* knockdown. *UQCR-C2* is involved in the mitochondrial respiratory system, and it is interesting to note that alteration of mitochondrial activity has been associated with extended life span in *Drosophila* *(Copeland et al., Curr Bio, 2009; Mahmoudi et al., Nat Cell Biol. 2019)*. Furthermore, increased expression of heat shock proteins such as hsp26 and hsp27 have been previously associated with increased longevity in *Drosophila* (*Wang et al., PNAS, 2004*). Our RNA-sequencing analyses with nervous-system specific knockdown of *UQCR-C2* in *Drosophila* identified high expression of multiple heat shock protein genes, including *hsp26*, which could potentially be linked with the observed phenotype. We hope that these observations can be followed up in flies and other systems, but we would like to be cautious in interpreting it in our manuscript until additional details on the mechanisms are available. We have modified the **Results** to include this previous observation.

***Results, page 8:*** *“We observed early lethality in adult flies with nervous system-specific Elav-GAL4-mediated knockdown of Sin and CG14182 (MOSMO) (Fig 2B), while extended lifespan was observed with knockdown of UQCR-C2, as previously reported for Hsp26, Hsp27 (35), and SOD (36). While altered mitochondrial activity has been shown to increase lifespan in Drosophila (37, 38), further studies are necessary to understand the mechanism underlying this phenotype observed with knockdown of UQCR-C2.”*

**(B)** We previously found that >50% of children with the deletion have some form of craniofacial changes (Pizzo *et al*., *Genet. Med.*, 2019). The observation of craniofacial features in *Xenopus* models suggests a mechanistic role for 16p12.1 homologs towards craniofacial development, as has been observed for other homologs of genes affected by CNVs using zebrafish, rat, and mouse models (Qiu *et al., Cell Reports,* 2019). In fact, a manuscript detailing the role of 16p12.1 homologs in craniofacial development has currently been posted on bioRxiv (*Lasser et al, bioRxiv, 2020*). We have modified the **Results** to incorporate this explanation:

***Results, page 10****: “Reduced expression of mosmo and polr3e led to severe craniofacial defects in stage 42 tadpoles, as measured by specific facial landmarks, while milder defects were observed for cdr2 (Fig 2H, S5 Fig). This suggests a role for these homologs in key developmental processes involved in craniofacial morphogenesis, such as neural crest cell formation and migration (43-48), and could potentially explain the craniofacial changes observed in more than 50% of individuals with the 16p12.1 deletion (9).”*

***Q7****. “When the results describe enhancement or reduction of single gene phenotypes in pairwise knockdowns, no interpretation of the direction is made with respect to a priori expectation. For example, based on whether human neurodevelopmental phenotypes are caused by loss of function vs. gain of function mutations, wouldn’t you predict a direction of interaction?* *Is it surprising to find roughly equal numbers of enhancers and suppressors?”*

**Authors’ response:** We started our studies with a plan for predicting interactions based on prior knowledge from human studies. After studying fly models for the 16p11.2 (*Iyer et al., Nat Comm. 2018*) and 3q29 deletions (*Singh et al., PLOS Genet. 2020*), we realized that predicting the number of *enhancers* or *suppressors* within a CNV region is complex, as it can depend on several factors such as dosage sensitivity of the gene, its location in an interaction network, and tissue-specific contexts. Studies of high-throughput interactions in yeast and other models have shown that *enhancers* typically represent genes that likely act in parallel biological pathways affecting the same function, while *suppressors* are genes that are direct interactors with likely biochemical relationships (*Dixon et al., Annu. Rev. Genet., 2009*). Since the biological function of most CNV genes is unknown, it would be challenging to predict the relationship of 16p12.1 homologs with other interacting genes. We expect that the number and identity of genetic interactions may change when we test interactions with larger sets of genes in various tissues, cell types, and at varying doses.

***Q8.*** *“How were these known neurodevelopmental genes chosen if there was no expectation or prediction made? In general, it would be helpful to explain the specific hypothesis and how the genes were chosen to test it, e.g. neurodevelopmental or from transcriptome studies are a little vague to understand the study design. Is 12/24 enhancers in the ‘second hits’ different from 25/52 (if I’m counting this the same) other developmental genes tested?”*

**Authors’ response:** Our screening of genetic interactions between homologs of 16p12.1 genes with potential interacting genes was performed with different hypotheses, depending on the functional classes of homologs tested:

**(1) Neurodevelopmental genes and functional related genes:** This category includes seven homologs of genes in different neurodevelopmental pathways, including fly homologs of genes with chromatin modulating (*CHD8*), cellular proliferation (*PTEN*), and synapse function (*SHANK3*). We also tested six genes with *functional relatedness* to individual 16p12.1 homologs, including genes with mitochondrial function (homologs of *COX1*, *COX2*, *ND2*, *ND3* and *ND4*) due to the role of *UQCRC2* in mitochondrial function (*Duncan et al., Genomics, 1993*), as well as homolog of *MYC*, which activates transcription of RNA polymerase III (multiprotein complex containing *POLR3E*) (*Gomez-Roman et al., Nature, 2003*) and is also associated with *CDR2* function in mammalian tumors (*O’Donovan el al., PLoS One, 2010*). Due to the related biological functions of these genes with the 16p12.1 homologs, we hypothesized a relatively high number of positive or negative interactions to be identified with these homologs compared with interactions between the 16p12.1 homologs. Using the multiplicative model, we identified interactions in 22/55 (40%) pairwise knockdowns, including negative interactions of *mt:ND3* with *UQCR-C2* and positive interactions of *Ube3a* with *UQCR-C2*, *Cen,* and *Sin*. In line with our hypothesis, the effect of only 3/12 (25%) pairwise combinations of 16p12.1 homologs could be explained by a genetic interaction, although this result did not reach statistical significance (22/55 versus 3/12; p=0.512, Fisher’s exact test).

**(2) Transcriptome targets and related genes:** We tested interactions of each 16p12.1 homolog with its downstream targets identified from RNA-sequencing experiments and with genes selected from gene ontology groups that were enriched among the differentially expressed genes (for example, RNA-seq targets of *Sin* were enriched for muscle contraction and nervous system development). Overall, this category comprised 61 interactions with 39 homologs, including 33 interactions with 25 homologs that were RNA-seq targets of one or more 16p12.1 homologs. We expected to identify a larger number of interactions of 16p12.1 homologs with this category of genes compared to their interactions with neurodevelopmental genes or with other 16p12.1 homologs, due to potential direct effects. We identified genetic interactions for 42/61 pairwise combinations (68.8%), compared to 40% interactions identified with neurodevelopmental genes (p=0.0027, Fisher’s exact test). Moreover, we observed a significantly higher proportion of interactions between 16p12.1 homologs and transcriptome targets (42/61), than of those observed between 16p12.1 homologs (3/12, Fisher’s exact test, p=0.0077), in line with our hypotheses that 16p12.1 homologs are less likely to interact or connect with each other compared to their direct transcriptome targets.

**(3) Homologs of patient-specific “second-hit” genes:** We selected patient-specific “second-hit” genes as disease-associated genes carrying rare (ExAC frequency ≤ 1%) copy-number variants, loss-of-function (frameshift, stopgain or splicing) mutations, or *de novo* or likely-pathogenic (Phred-like CADD ≥25) missense mutations previously identified from exome sequencing and SNP microarrays in affected children with the 16p12.1 deletion (*Pizzo et al., Genet Med., 2019*). This selection led to a total of 24 homologs of patient-specific second-hit genes. For example, a proband with the 16p12.1 deletion who also carried a *de novo* loss-of-function mutation in *SETD5* had more severe neurodevelopmental phenotypes than their carrier parents. In fact, we have previously reported a higher burden of “second-hits” in probands with the 16p12.1 deletion compared with their mildly affected carrier parents (*Girirajan et al., NEJM, 2012*; *Pizzo et al., Genet Med., 2019*). We expected to identify that “second-hits” modulated phenotypes of 16p12.1 homologs mostly through additive effects and fewer genetic interactions (as reviewed in Veltman and Brunner, *Nature Genet.*, 2010). Since the *SETD5* mutation increased the severity of 16p12.1 deletion, we further hypothesized that one or more 16p12.1 homologs would show synergistic effects with *SETD5*. Out of the 96 combinations tested, we identified 32 pairwise knockdowns that modulated the phenotype of a 16p12.1 homolog, and 37 could be attributed to genetic interactions based on the multiplicative model. Of the 32 pairwise knockdowns that modulated the phenotype of a 16p12.1 homolog, 16 pairs corresponded to positive or negative genetic interactions.

To summarize the hypotheses tested in this part of our study, we have added a table detailing the hypothesis, rationale for each experiment, observed results, and conclusions (**S3 Table**/**Table R6**), and generated a new **S10 Figure** (**Figure R8**). We have also modified the **Results** section to include the hypothesis behind each experiment, and the **Discussion** section to compare the different results obtained in our interaction studies and their alignment with our hypotheses:

***Results, page 14:*** *“As our functional analyses showed that knockdown of each 16p12.1 homolog resulted in multiple neuronal and developmental phenotypes, we hypothesized that genes involved in conserved neurodevelopmental pathways could modulate phenotypes due to knockdown of 16p12.1 homologs through genetic interactions... First, we screened for 55 combinations between homologs of 16p12.1 genes and 13 homologs of human genes in established developmental pathways, such as synapse function (Prosap/SHANK3), cell division (Pten/PTEN), and chromatin modulation (kis/CHD8), as well as genes functionally related to 16p12.1 homologs (54, 59-61).”*

***Results, page 15:*** *“Next, to correlate 16p12.1 homologs to developmental functions and pathways (S10 Fig, S3 Table), we screened for interactions of the homologs with 25 dysregulated fly genes selected from our transcriptome studies as well as 14 genes within enriched Gene Ontology categories, such as nervous system development and function (Dscam1, Asp, mGluR, NaCP60E), protein folding (Hsp23, Hsp26, Hsp70Ab), and muscle contraction (Actn, ck).”*

***Results, page 16:*** *“We hypothesized that homologs of genes carrying patient-specific “second-hits” modulate the effects of individual 16p12.1 homologs not only additively but also through genetic interactions (S10 Fig, S3 Table)… Out of the 96 combinations tested, we identified 32 pairwise knockdowns that modulated the phenotype of a 16p12.1 homolog, confirmed with additional lines when available, for 11 out of 15 families carrying “second-hit” genes (Fig 5A-C, S2-S3 Tables). In fact, the phenotypic effects of 16 out of 32 combinations were attributed to genetic interactions (Fig 4D, S3 File).”*

***Discussion, page 19:*** *“Third, we found that the 16p12.1 homologs were less likely to interact with each other (3 interactions out of 12 pairs tested) compared to their interactions with downstream transcriptome targets (42 interactions out of 61 pairs tested, Fisher’s exact test, p=0.0077). These results suggest reduced functional overlap among the 16p12.1 homologs, an observation supported by the distinct sets of biological functions enriched among the differentially expressed genes obtained with knockdown of each individual homolog (S4 Fig).”*

***Legend S10 Figure, page 63****: “…These hypotheses aligned with our results, as we identified genetic interactions for 42/61 pairwise combinations (68.8%) with direct transcriptome targets, compared to 22/55 (40%) interactions identified with functionally related and neurodevelopmental genes (p=0.0027, Fisher’s exact test). Moreover, we observed that homologs of genes carrying “second-hits” in severely affected children with the 16p12.1 deletion interacted with 16p12.1 homologs in 37/96 (38.5%) of the pairs, although the proportion of genetic interactions was not as high compared to that identified with functionally related genes or genes in neurodevelopmental pathways and transcriptome targets (64/101, 63%, Fisher’s exact test, p=0.019).”*

**Table R6. Hypotheses, experimental design, and conclusions obtained for experiments performed in our study.**

| **EXPERIMENT** | **Individual knockdown of 16p12.1 homologs** | **Pairwise knockdown of 16p12.1 homologs** | **Pairwise interactions of 16p12.1 homologs with "Neurodevelopmental genes"** | **Pairwise interactions of 16p12.1 homologs with "Transcriptome targets"** | **Pairwise interactions of 16p12.1 homologs with "Patient-specific second-hit genes"** | **Cellular phenotypes** |
| --- | --- | --- | --- | --- | --- | --- |
| **HYPOTHESIS TESTED** | Multiple 16p12.1 homologs contribute to neurodevelopmental phenotypes | 16p12.1 homologs interact towards neurodevelopmental phenotypes | 16p12.1 homologs interact with genes within core neurodevelopmental pathways and functionally related genes towards neurodevelopmental phenotypes | 16p12.1 homologs interact with their downstream genes towards neurodevelopmental phenotypes | Patient-specific “second-hits” can modulate neurodevelopmental phenotypes of 16p12.1 homologs through genetic interactions | Genes interact with 16p12.1 homologs towards neuronal defects by altering cellular phenotypes |
| **EXPERIMENTAL STRATEGY** | Individual knockdown of 16p12.1 homologs using tissue-specific drivers in *Drosophila* and whole-embryo knockdown in *X. laevis*, and assessment of multiple neuronal, cellular and developmental phenotypes | Simultaneous knockdown of 16p12.1 homologs - assessment of genetic interactions in the fly eye. Assessment of specific pairs of homologs towards brain and cellular phenotypes in *X. laevis* | Simultaneous knockdown of 16p12.1 homolog with neurodevelopmental genes and functionally related genes - assessment for genetic interactions in the fly eye | Simultaneous knockdown of 16p12.1 homolog with transcriptional targets or their functionally related genes - assessment for genetic interactions in the fly eye | Simultaneous knockdown of 16p12.1 homologs with homologs of patient-specific “second-hit” genes - assessment for genetic interactions in the fly eye | Assessment of cellular proliferation and apoptosis processes for validated two-hit interactions in the developing fly eye |
|  |  |  | Genes in established neurodevelopmental pathways - 7 genes; Genes functionally related to 16p12.1 homologs - 6 genes | Up and down-regulated genes from transcriptome analysis of 16p12.1 homologs - 25 genes; Genes within enriched GO terms from transcriptome analysis - 14 genes | Homologs of “second-hit” genes from sequencing children with 16p12.1 deletion - 24 genes |  |
|  |  | 12 combinations | 55 combinations | 61 combinations | 96 combinations | 3 combinations |
| **OUTCOME** | Global and homolog-specific phenotypes | 3/12 positive interactions | 22/55 interactions | 42/61 interactions | 37/96 interactions | Alterations of proliferation and apoptosis processes compared to single knockdown of 16p12.1 homolog |
| **EXPERIMENT** | **Individual knockdown of 16p12.1 homologs** | **Pairwise knockdown of 16p12.1 homologs** | **Pairwise interactions of 16p12.1 homologs with "Neurodevelopmental genes"** | **Pairwise interactions of 16p12.1 homologs with "Transcriptome targets"** | **Pairwise interactions of 16p12.1 homologs with "Patient-specific second-hit genes"** | **Cellular phenotypes** |
| **CONCLUSIONS** | Each 16p12.1 homolog contributes towards a range of developmental, neuronal, and cellular defects in flies/*X. laevis* | 16p12.1 homologs contribute towards neurodevelopmental phenotypes through mild genetic interactions and additive effects | 16p12.1 homologs interact with genes in core pathways towards neurodevelopmental phenotypes | Differentially expressed genes interact with 16p12.1 homologs towards neurodevelopmental phenotypes | Patient-specific second-hits can modify phenotypes of 16p12.1 homologs through synergistic and alleviating interactions | Interacting genes modulate proliferation and apoptosis phenotypes of 16p12.1 homologs during development |
| **HYPOTHESIS-GENERATING RESULTS** | Validation of phenotypes associated with each homologs in other model organisms | Assessment of identified interactions in specific neuronal cell types | Follow up of identified interactions (such as those involving homologs of *MOSMO* and *SETD5*) in other model organisms and human cell lines/organoids. | | | Assessment of mechanisms connecting genes and interactions to cellular defects in specific neuronal cell types |

***Q9.*** *“In general, it gets a little confusing to count number of genes (in and out of the CNV) tested, number of total pairs (considering different lines from the same gene), number of unique pairs, etc., so greater clarity in how these are being counted, tested, and considered (and why) would be helpful.”*

**Authors’ response:** We thank the reviewer for seeking clarity on this point. We now provide **S2 Table/Table R2** on **Page 5** of this document indicating all genes and pairwise combinations tested, **and S3 Table**/**Table R6** on **Page 24** of this document, indicating the hypothesis, experiments results, and conclusions drawn from our study. We hope this will help the reader better follow the results in the text.

***Q10****. “Overall, this may be a valuable dataset and interesting theory, but clear delineation of the study design and interpretation and contextualization of the results is needed for the reader to understand it fully. It might be helpful to specify upfront whether some experiments are meant to be hypothesis generating but require additional testing or follow-up, e.g. specific pairs of genes that might interact in humans, and clarify hypotheses and testing approaches for the others, e.g. what were you intending to demonstrate about second-hit genes (vs neurodevelopmental genes?) and how did you specifically test that your results supported that hypothesis.”*

**Authors’ response:** We thank the reviewer for this constructive comment. We started our studies with specific hypotheses, which we have clarified in a new supplemental table (**S3 Table /Table R6** shown in **Page 24** in this document) detailing the hypothesis, experiment, results, and conclusions for each experiment. However, new findings that have resulted from our work can be further validated and expanded in other model organisms.

**A note on data availability**: We note that RNA-sequencing datasets have been uploaded in public repositories, including GEO (acc#GSE151330), SRA (acc#PRJNA635495), and Github (https://github.com/girirajanlab/16p12_fly_project), summary statistics information for all experiments performed has been provided in S6 File, and all experimental data has been provided as individual values in main and supplemental figures. Observed and expected values obtained for all the genetic interaction analyses are provided in S4 File.

**REVIEWER 2**

“*The paper entitled “Functional assessment of the “two-hit” model for neurodevelopmental defects in Drosophila and X. laevis” discusses the potential of the 16p12.1 deletion to sensitize probands to the advent of neurodevelopmental disease. In this manuscript, the authors suggest that the knockdown of 16p12.1 homologs can result in a diverse range of phenotypes, including delayed development, seizure susceptibility, brain malformations, and abnormal neuronal morphology. Two different pairwise assessments were performed examining the interaction of genes within the 16p12.1 deletion with each other and with putative “second-hit” genes identified from patients and examination of associated developmental pathways. In the cases studied, changes in eye phenotypes were observed in putatively interacting homologs from 11/15 families described. In support of their claims, the authors provide the following information:
1. The 16p12.1 deletion is inherited in more than 95% of individuals carrying the deletion, and affected children are more likely to carry another large CNV or deleterious mutation. The authors suggest this indicates that the 16p12.1 deletion confers significant risk for disease/sensitizes the genome, while other variation explains ultimate phenotype.
2. The loss of individual genes from the 16p11.2 and 3q29 regions is insufficient to explain the wide range of defects observed with the loss of the entire region.
3. Previous work with the 16p11.2 and 3q29 regions demonstrated pervasive interactions of homologs within regions associated with neurodevelopmental disease.
4. Four fly homologues (of the 7 human genes in 16p12.1) were identified: UQCR-C2, CG14182, Sin, and Cen. These have homologues in Xenopus as well.
5. Use of RNAi against UQCR-C2, CG14182, Sin, and Cen to induce either ubiquitous or tissue specific knockdown lead to a plethora of phenotypes, depending on the Gal4 line used. Knockdown efficiency of the RNAi was ~40-60%.
6. RNA-seq was performed on fly heads with pan neuronal knockdown of 16p21.1 homologues. Gene ontology analysis of differentially expressed genes demonstrated enrichments for cellular, developmental, and neuronal processes.
7. Xenopus was used to examine developmental phenotypes when homologues were knocked down using morpholinos. Two “strengths” of knockdown were used- partial knockdown (50%) and “further reduced” (approx. 10-40% of baseline). Knockdown resulted in alterations in craniofacial defects, axon outgrowth phenotypes, and decreased forebrain and midbrain size.
8. Use of GMR-Gal4 lines to study the eye revealed that knockdown of the 4 homologues could result in disruptions of the ommatidial organization, as identified by flynotyper. These phenotypes were more severe in the presence of Dicer2. Pairwise interactions (two RNAi lines) resulted in more severe phenotypes with some pairs.
9. Pairwise interactions in Xenopus was performed using partial (50%) knockdowns. Pariwise knockdown of mosmo and polr3e demonstrated reduced forebrain/midbrain size.
10. 166 pairwise gene interactions were attempted using flynotyper. 18 neurodevelopmental genes, 25 genes from the authors transcriptome studies, and 14 genes within functionally related pathways were identified as having an interactive effect.
11. 11. RNAi or knock outs of 24 homologs of patient specific “second hit” genes were tested with the RNAi of UQCR-C2, CG14182, Sin, and Cen. “Second hit” genes were shown to be able to act as both enhancers and suppressors, depending on the context. Similar interactions were observed in the Xenopus model, in particular the authors used the example of mosmo and set5, which had a significant effect on axon length.*

*After evaluating the documents provided, I believe this paper has several significant deficiencies that require further explanation or additional experiments to address. Due to the scope and nature of some of these critiques, it may not be possible to address them within a short period of time. The following should be addressed by the authors:* *Major Critiques:*”

***Q1.*** *“The authors note that RNAi knockdown of the 4 homologues resulted in a decrease in expression level of 40-60%. This calls into question if the remaining proteins are sufficient to restore functionality and if non-significant results may be due to residual functionality.”*

**Authors’ response:** We sincerely thank the reviewer for providing a detailed summary of our manuscript. We appreciate the reviewer’s comment, and agree with the reviewer that non-significant results could be caused by residual functionality of the genes due to 40-60% residual expression of the homologs. The logic behind assessing phenotypes with approximately 50% of residual expression is that the deletion is heterozygous in humans, and therefore 50% of gene expression would be sufficient to sensitize an individual towards neurodevelopmental phenotypes (i.e. heterozygous deletions lead to haploinsufficiency of genes within the deletion). In fact, our results suggest that further reduction of gene expression could lead to more severe phenotypes. *For example*, our *X. laevis* experiments were performed with partial (approximately 50% of expression) and full knockdown (approximately 20% of expression) of the genes, and some results, such as brain size for *polr3e* and axon outgrowth for *mosmo,* were only observed with full knockdown (see **S5 Figure** and **Figure 6**), suggesting dosage sensitivity of the genes towards certain neurodevelopmental phenotypes.

***Q2.*** *“Figure 2A is confusing as it does not address the Gal4 line used to generate the reported phenotypes. Furthermore, pupal lethality is not reported anywhere in this paper save this graph.”*

**Authors’ response:** We apologize the reviewer for not making these points clear. We have modified **Figure 2A** (represented as **Figure R9**) to include the type of tissue-specific knockdown and the drivers used in the figure and the legend. We have also modified the **Results** section to include the pupal lethality phenotype observed with knockdown of *UQCR-C2*.

***Results section, page 8****:* “*We first assessed the global role of 16p12.1 homologs during development by decreasing their expression ubiquitously using the da-GAL4 driver, and detected larval lethality with knockdown of Sin (POLR3E) and larval and pupal lethality with knockdown of UQCR-C2 (UQCRC2) (Fig 2A, S2 Fig).”*

***Figure R9.*** ***Multiple homologs of 16p12.1 genes contribute to neurodevelopmental defects in Drosophila melanogaster and X. laevis.*** *(A) Schematic showing multiple phenotypes affected by tissue-specific knockdown of individual 16p12.1 homologs in Drosophila melanogaster. Ubiquitous knockdown was achieved with da-GAL4, eye-specific knockdown with GMR-GAL4, wing-specific knockdown with bxMS1096-GAL4, and nervous system-specific with ppk-GAL4 or Elav-GAL4. See S2A-C Figure for details on phenotypes observed for individual fly lines.*

***Q3.*** *“The trajectory of the Elav-Gal4 control in figure 2B is hidden within the graph, and therefore impossible to evaluate.”*

**Authors’ response:** We apologize that the control was not easily visible in the graph. We have now moved the control data forward in this figure (shown below as **Figure R10/Figure 2B**).

***Figure R10. Multiple homologs of 16p12.1 genes contribute to neurodevelopmental defects in Drosophila melanogaster and X. laevis.*** *(B) Nervous-system mediated knockdown using Elav-GAL4 with overexpression of Dicer2 at 25°C led to reduced lifespan with knockdown of CG14182GD2738_2 (n=100, one-way repeat measures ANOVA with post-hoc pairwise t-test, days 6-61, p<0.05) and increased lifespan with knockdown of UQCR-C2GD11238 (n=120, days 51-81, p<0.05). Elav-GAL4 mediated knockdown of SinGD7027 at RT without overexpression of Dicer2 led to reduced lifespan of adult flies (n=160, day 1-6, p<0.05). Data represented show mean ± standard deviation of 4-8 independent groups of 20 flies for each line tested.*

***Q4.*** *“Given that multiple controls are used throughout the paper, controls need to be labeled properly/completely throughout the figures, not just as control.”*

**Authors’ response:** We thank the reviewer for this comment. We have modified the Main **Figures 3, 4, 5 and 6** and Supplemental figures to include the appropriate background-specific controls that were crossed with the driver-specific *GAL4* line: *Control^BL^ (BDSC #36303), Control^GD^ (VDRC #60000), and Control^KK^ (VDRC #60100)*.

***Q5.*** *“It is unclear how the authors chose which RNAi lines to display in Fig 2. From looking at the other figures, it is clear that multiple RNAi lines exist for each of the homologues, however only a small number of them are displayed here and in some cases are inconsistent between figures (such as CG14182^GD2738_2^). Furthermore, while the authors consider a trend to be “valid” when confirmed by multiple RNAi lines later in the paper, it is unclear what criterion is used to consider the lines corresponding to the homologues lines “valid”. For example: figure S8 when compared to S7. CG14182^GD2738_2^ showed a significant phenotype by itself when GMR-Gal4 was used to drive the RNAi. However, CG14182^GD2738^ was chosen for all future pair-wise experiments, and it is unclear why.”*

**Authors’ response:** We thank the reviewer for this comment. We would like to clarify the following points:

**(1)** RNAi lines used for 16p12.1 homologs were validated by quantifying their knockdown efficiency. As provided in the **Supplemental S1A Figure (Figure R11)**, all RNAi lines achieved final gene expression levels of 40-60%. The only exceptions were *Sin^KK101936^*, *Sin^HMC03807^*, and *UQCR-C2^KK108812^*, which showed embryonic lethality with *Elav-GAL4*. *Sin^GD7027^* line had ~60% expression and showed early lethality, with progeny dying on day 4-6 (**Figure 2B**). Therefore, we believe that early lethality observed for the non-viable lines is likely caused by stronger knockdown of the 16p12.1 homologs. As all lines presented similar knockdown levels, we decided to select one line to continue with pairwise knockdown experiments. Various factors were considered while selecting RNAi lines to generate recombinant lines, including lethality with *Elav*-*GAL4*, chromosomal insertion of the shRNA, presence of balancers, and severity of eye phenotypes with *GMR*-*GAL4*.

**(2)** In the case of *CG14182^GD2738^* and *CG14182^GD2738_2^*, both lines led to very similar levels of expression (40-50%, see **Supplemental S1A Figure/Figure R11**). These were used indistinctively, as they carried the same shRNA construct and only differed in the genomic insertion site. Moreover, both lines led to similar eye phenotypes when their expression was reduced using *GMR-GAL4* with overexpression of *Dicer2*. For interaction experiments, we sought to follow experimental conditions that would allow a wide range of double knockdown phenotypes (i.e. identify interactions leading to both mild and severe eye phenotypes). Therefore, we generated *GMR-GAL4* recombinant lines of the homologs without overexpression of *Dicer2*. As *CG14182^GD2738_2^* led to eye phenotypes under these conditions, while *CG14182^GD2738^* did not, we used the RNAi line that showed milder eye phenotype for our genetic interaction studies, consistent with our purpose of testing a wider range of effects with double knockdown. We have modified the **Methods** and **Results** section to clarify how the fly lines for the 16p12.1 homologs were validated and selected for the generation of recombinant lines.

***Methods section, page 31****: “Various factors including presence of balancers, chromosomal insertion of the shRNA, lethality with Elav-GAL4, and severity of eye phenotypes with GMR-GAL4 were considered to select RNAi lines for generating recombinant lines. For example, for CG14182, we used the GD RNAi line CG14182GD2738, which showed milder eye phenotypes, in order to test a wider range of potential interactions.”*

***Results section, page 8****: “We authenticated the RNAi lines by confirming 40-60% expression of the four homologs using RT-qPCR (S1 Fig).”*

**Figure R11. Expression levels of 16p12.1 homologs in Drosophila and X. laevis.**

(A) Drosophila homologs of 16p12.1 genes were knocked down using nervous system-specific Elav-GAL4 driver with overexpression of Dicer2 at 25°C. RT-qPCR confirmed 40-60% knockdown of the 16p12.1 homologs (two-tailed student’s t-test, *p<0.05).

***Q6.*** *“What the authors intend to indicate with lines 198-200 (uniquely disrupted GO terms) is not clear. GO terms are descriptive, and the authors should provide information about the genes affected, not a broad argument about systems affected.”*

**Authors’ response:** Our initial analysis focused on the percentage of uniquely disrupted Gene Ontology (GO) terms among all identified GO terms from the differentially expressed genes obtained from knockdown of each 16p12.1 homolog in the nervous system. To better visualize the independent effects of 16p12.1 homologs towards neurodevelopment, we have now included the percentage of unique differentially-expressed genes for each homolog along with their human counterparts. We have modified the **Results** (excerpt below) to add examples of genes; and added **S4 Figure** (**Figure R12**) to show the overlap of fly homologs and human counterparts.

***Results, page 9****: “We found that each 16p12.1 homolog disrupted unique sets of genes and biological functions, as 1,386/1,870 (74%) differentially expressed genes and 28/52 (53.8%) enriched GO biological process terms were uniquely disrupted by one homolog (S4 Fig). Notably, we also observed this trend among the human homologs of differentially-expressed genes, with 654/994 (65.8%) uniquely differentially expressed genes and 353/428 (82.5%) GO terms uniquely disrupted by the 16p12.1 homologs, suggesting that they may act within independent pathways (S4 Fig).”*

***Results, page 10****: “For example, knockdown of CG14182 altered the expression of fly homologs of human genes involved in synapse assembly and transmission (NLGN1, CEL) as well as histone methyltransferase binding (NOP56, CBX1). Similarly, human homologs of genes differentially expressed with knockdown of Sin were involved in neuronal projection, neurotransmitter release (such as CHRNA7, KCNAB2, and multiple solute carrier transport family genes, including SLC6A1), and GABA pathways (such as ADCY2, ADCY4, and ADCY7), as well as in the development of several non-neuronal organ systems, including cardiac, kidney, lung, and muscle, further indicating the importance of Sin towards global development.”*

***Figure R12. Enriched GO terms observed with knockdown of 16p12.1 fly homologs in the nervous system.*** *(A) Clusters of enriched Gene Ontology (GO) Biological Process terms for differentially expressed fly genes observed with nervous system-specific knockdown of 16p12.1 homologs (left) and their human homologs (right). While some clusters of terms overlap among 16p12.1 homologs, genes dysregulated with knockdown of individual homologs exhibit unique enrichments for GO terms, suggesting their independent action towards neuronal development. Venn diagrams show overlaps of enriched GO Complete Biological Processes terms for (B) differentially-expressed fly genes observed with knockdown of individual 16p12.1 homologs, or (C) human homologs of the fly genes. We also observed that most of the (D) fly homologs or (E) human counterparts of the differentially-expressed genes were unique to each 16p12.1 homolog. A list of differentially expressed genes with knockdown of 16p12.1 homologs, as well as a list of all enriched GO terms for these gene sets, is detailed in S2 File. Venn diagrams were constructed using Venny 2.1 software (https://bioinfogp.cnb.csic.es/ tools/venny).*

***Q7.*** *“30 degrees C is a high temperature to raise flies at, and may accentuate phenotypes.”*

**Authors’ response:** We agree that 30°C is a high temperature to raise flies. However, this temperature has been previously used by our work (*Iyer et al., Nat Comm, 2018; Iyer et al., G3, 2016*) for rearing flies with eye and nervous-system specific RNAi knockdowns. While this temperature leads to more severe eye phenotypes, these experiments are performed in comparison to controls which are also exposed to the same conditions.

***Q8.*** *“It is possible that the expression of any RNAi (or combination or RNAi’s) may be deleterious in the fly eye. The authors do not appear to have a control which can account for this. It would be optimal to show that an RNAi which does not have a potential target (RNAi against LacZ or similar) causes no effect.”*

**Authors’ response:** We thank the reviewer for this comment. Previous studies have shown that the fly eye is a powerful tool to assess for genetic interactions, as about 2/3^rd^ of the genome is involved in *Drosophila* eye development, with about 75% of human disease genes having orthologs in flies (*Thaker and Kankel, Genetics, 1992; Reiter et al., Genome Res, 2001*).

**(a)** The fly eye has been classically used for modifier screens and to identify genetic interactions for several neurological disorders (*Karim et al., Genetics, 1996; Bilen and Bonini, PLOS Genet., 2007*). For example, high throughput assays for the Rett syndrome gene, *MeCP2*, identified several interacting genes that compensated for the defects caused by increased activity of *MeCP2* (*Cukier et al, PLOS Genet., 2008*).

**(b)** To address this comment, we assessed the number of RNAi lines that showed significant eye phenotypes versus no eye phenotypes using *Flynotyper* which is sensitive to small changes in ommatidial disorderliness (*Iyer et al., G3 2016*). We analyzed a total of 103 fly lines for 69 genes, including 31 genes for which we evaluated two or more RNAi lines. We found that 44 out of 103 lines showed a significant eye phenotype with a higher *Flynotyper* score compared to controls (two-tailed Mann-Whitney, Benjamini-Hochberg correction, p<0.05). Interestingly, 14 out of 31 genes did not show a phenotype in two or more RNAi lines, while 12 out of 31 genes developed a significant eye phenotype that was validated in multiple lines. Furthermore, multiple studies have shown that the use of scramble RNAi does not cause any phenotypic effect in the *Drosophila* eye or other tissues (*Fulga et al., Nat Comm. 2015; Mills et al., eLIFE 2019*). Thus, the mere presence of an RNAi line is not enough to cause deleterious effects in the fly eye compared with control, and we can attribute eye defects directly to the reduced expression of each gene in multiple lines. Results from this analysis are represented in **Table R7**.

**Table R7. Phenotypic assessment of RNAi lines**

| Total RNAi lines analyzed | 103 |
| --- | --- |
| RNAi lines with phenotype | 44 |
| Total number of genes assessed | 69 |
| Number of genes assessed with >2 RNAi lines | 31 |
| Number of genes with >2 lines that have a phenotype | 12 |
| Number of genes with >2 lines that DO NOT have a phenotype | 14 |

***Q9.*** *“As stated by the authors, “second-hit” genes were tested with multiple RNAi, mutant or overexpression lines. No information about the efficiency of knockdown on these RNAi lines is available.”*

**Authors’ response:** We thank the reviewer for this comment. We have available RT-qPCR data from selected lines used for our screening experiments, shown below in **Figure R13**. While these only represent a few lines used in our study, each of the lines we used in our study have been extensively used in other publications. We present a table citing studies that have previously used the RNAi, overexpression, and mutant lines used in our study (**Table R8**).

***Figure R13. Expression data of selected RNAi lines for interacting genes tested in this study.***

**Table R8. Studies that have used the *Drosophila* lines used in this study.**

| ***Gene name*** | ***Studies that have used the RNAi line*** |
| --- | --- |
| *kis^GD16331^* | *Singh et al., Plos Genet 2020; Iyer et al., G3 2016; Zhu et al., eLife 2017; Ghosh et al., PLoS ONE* |
| *kis^KK100890^* | *Singh et al., Plos Genet 2020* |
| *para^GD3392_1^* | *Singh et al., Plos Genet 2020; Iyer et al., G3 2016; Xiao et al,; Front Mol Neuro 2017* |
| *para^GD3392_2^* | *Singh et al., Plos Genet 2020; Iyer et al., G3 2016* |
| ***Gene name*** | ***Studies that have used the RNAi line*** |
| *Pten^GD13500^* | *Singh et al., Plos Genet 2020; Iyer et al., G3 2016; Hevia et al., Genetics 2017* |
| *Pten^KK109278^* | *Singh et al., Plos Genet 2020; Iyer et al., G3 2016; Gerlach et al., Cell Rep 2018* |
| *Prosap^GD10101^* | *Singh et al., Plos Genet 2020; Iyer et al., G3 2016; Schwartz et al., Genetics 2016* |
| *Prosap^KK101537^* | *Singh et al., Plos Genet 2020; Iyer et al., G3 2016; Schwartz et al., Genetics 2016* |
| *Ube3a^GD450^* | *Singh et al., Plos Genet 2020; Iyer et al., G3 2016; Straub et al., Sci Rep 2020* |
| *Ube3a^KK104898^* | *Singh et al., Plos Genet 2020; Iyer et al., G3 2016* |
| *CG10465^KK105756^* | *Singh et al., Plos Genet 2020; Yusuff et al, Plos Genet 2020* |
| *rl^GD12910_1^* | *Iyer et al., Nat Comm 2018* |
| *rl^GD12910_2^* | *Iyer et al., Nat Comm 2018* |
| *rl^KK115768^* | *Iyer et al., Nat Comm 2018* |
| *mt:ColI^KK116296^* | *N/A* |
| *mt:CoI^KK115361^* | *N/A* |
| *mt:ND2^KK116148^* | *N/A* |
| *mt:ND3^KK115930^* | *N/A* |
| *mt:ND4^KK115665^* | *N/A* |
| *Myc^4^* | *N/A* |
| *Myc^Overexp Myc.Z132^* | *Kim et al., J Biol Chem 2015; Thevenon et al Front Cell Dev Biol 2020* |
| *Myc^Overexp Myc.Z42^* | *N/A* |
| *Myc^JF01761^* | *Gamberi et al., Plos Genet 2017* |
| *Myc^GL01314^* | *Thevenon et al Front Cell Dev Biol 2020* |
| *upSET^HMC03177^* | *Zhou et al., Sci Adv 2019* |
| *upSET^HMJ23045^* | *Rotelli et al., G3 2019* |
| *LanB2^HMJ23628^* | *Rotelli et al., G3 2019* |
| *LanB2^HMC04076^* | *Dai et al., Plos Genet 2018* |
| *Dys^HMC03789^* | *Rotelli et al., G3 2019* |
| *Dys^JF01118^* | *Yan et al., Dev Cell 2014* |
| *Dpm1^GLC01664^* | *N/A* |
| *Dpm1^HMS02947^* | *Pletcher et al., G3 2019; Zhang et al., eLife 2015* |
| *Dhc36C^HMC03270^* | *Zhou et al., Sci Adv 2019* |
| *Dhc36C^HMJ23543^* | *Rotelli et al., G3 2019* |
| *Cep135^HMC06109^* | *N/A* |
| *Cep135^GD6121_1^* | *Balmer et al., Dev Cell 2015; Neely et al., Cell 2010* |
| *Cep135^GD6121_2^* | *Neely et al., Cell 2010* |
| *Zasp52^HMJ22168^* | *N/A* |
| ***Gene name*** | ***Studies used the line*** |
| *Zasp52^JF01133^* | *Liu et al., J Cell Sci. 2013* |
| *osa^GL00372^* | *Yan et al., Dev Cell 2014* |
| *osa^HMS01738^* | *Chubak et al., Dis Mod Mech 2019* |
| *osa^JF01207^* | *Dietz et al., Hum Mol Genet 2015* |
| *Pex1^HM05190^* | *Zhou et al., Sci Adv 2019* |
| *Pex1^HMC03252^* | *Zhou et al., Sci Adv 2019* |
| *GlyP^k07918^* | *N/A* |
| *GlyP^HMS00032^* | *Bai et al., Plos Genet 2013* |
| *shot^GL01286^* | *Baskar et al., Biol Open 2019; Hassan et al., G3 2018* |
| *shot^HMJ23381^* | *Watanabe et al., G3 Feb 2020* |
| *shot^JF02971^* | *Hassan et al., G3 2018* |
| *Rpn2^HMS00533^* | *Zhou et al., Sci Adv 2019* |
| *PDZ-GEF^HM05139^* | *Raza et al., Plos Genet 2018* |
| *PDZ-GEF^JF01765^* | *Yan et al., Dev Cell 2014* |
| *na^har38^* | *Nash et al., Curr Biol 2022* |
| *na^JF01826^* | *Ghezzi et al., Front Mol Neuro 2014* |
| *CalpA^JF03391^* | *Buffolo et al., Genesis 2015* |
| *Pde6^HMS01485^* | *Yan et al., Dev Cell 2014* |
| *Pde6^JF01846^* | *Kuntz et al., Curr Biol 2017* |
| *Usp16-45^EY19835^* | *Yagi et al., Genes Cells 2013* |
| *AstC-R2^GL01063^* | *Yan et al., Dev Cell 2014* |
| *AstC-R2^JF01960^* | *Yu et al., eLife 2016* |
| *Dhc98D^MB03402^* | *N/A* |
| *cac^JF02572^* | *Raza et al., Plos Genet 2018* |
| *Cadps^GD9502_1^* | *Singh et al., Plos Genet 2020; Iyer et al., G3 2016; Scholz et al JCB 2019* |
| *Cadps^GD9502_2^* | *Singh et al., Plos Genet 2020; Iyer et al., G3 2016* |
| *nAChRα7^KK108471^* | *Singh et al., Plos Genet 2020; Tsubouchi et al., Curr Biol, 2012* |
| *nAChRα7^GD3742^* | *Singh et al., Plos Genet 2020; Tsubouchi et al., Curr Biol, 2012* |
| *nAChRα6^GD1045^* | *Singh et al., Plos Genet 2020; Tsubouchi et al., Curr Biol, 2012* |
| *Nrx-1^GD2619^* | *Singh et al., Plos Genet 2020; Neely et al., Cell 2010* |
| *Nrx-1^GD14451_1^* | *Singh et al., Plos Genet 2020; Neely et al., Cell 2010* |
| *Nrx-1^GD14451_2^* | *Singh et al., Plos Genet 2020; Neely et al., Cell 2010* |
| *CG32301^GD16147^* | *Chen et al, Science 2010* |
| *Actn^GD1354^* | *Clark et al., Cytoskeleton (Hoboken), 2013* |
| *Asp^GD1375^* | *Singh et al., Plos Genet 2020; Nauert et al., Dev. 2017* |
| ***Gene name*** | ***Studies used the line*** |
| *CG42260^KK111286^* | *Lee et al., Arch. Insect Biochem. Physiol 2020* |
| *ck^KK102922^* | *Orme et al., Nat Commun 2016* |
| *CG9150^GD6560^* | *Neely et al., Cell 2010* |
| *Drice^17_1^* | *Xu D et al, Cell Death Differ. 2006* |
| *Drice^17_2^* | *Xu D et al, Cell Death Differ. 2006* |
| *Dscam1^GD14362^* | *Neely et al., Cell 2010* |
| *Dscam1^KK100296^* | *Kang et al., Mol Brain 2019* |
| *Dscam1^HMS01859^* | *Kamiyama et al., Dev Cell 2015* |
| *Dscam4^HMC03277^* | *Zhou et al., Sci Adv 2019* |
| *sn^KK108890^* | *N/A* |
| *sn^GD8993^* | *Neuert et al., Development 2017* |
| *f^KK102771^* | *N/A* |
| *CG14515^KK110660^* | *N/A* |
| *GluRIIB^Overexp EGFP^* | *Schmid A. et al, Nat Neuroscience, 2008.* |
| *CG30059^GD4893^* | *Neely et al., Cell 2010* |
| *Hsp23^LL01535^* | *Kawasaki et al., Dis Model Mech 2016* |
| *Hsp23^Df(3L)AC1^* | *N/A* |
| *Hsp23^Overexp Hsp23.K2^* | *Kawasaki et al., Dis Model Mech 2016* |
| *Hsp26^Overexp Hsp26^* | *N/A* |
| *Hsp70Ab^Overexp Hsp70-9.1^* | *Xiao C. et al, Dev Neurobiol, 2007.; Kawasaki et al., Dis Model Mech 2016* |
| *Hsp70Aa-Hsp70Bb^Df(3r)Hsp70A-Hsp70B^* | *Štětina, et al., Plos One 2015* |
| *Hsp70Ab^Overexp Hsp70-4.3^* | *Xiao C. et al, Dev Neurobiol, 2007* |
| *CG34195^KK103308^* | *Iyer et al., Nat Comm 2018* |
| *mGluR^JF01958^* | *Yan et al., Dev Cell 2014* |
| *Myo10A^KK108171^* | *N/A* |
| *didum^GD1848^* | *Harris et al., eLife 2016* |
| *CG18233^KK104071^* | *Katz et al., PNAS 2014* |
| *Bace^GD5366^* | *Penserga et al., Fron Cell Neurosci 2019* |
| *NaCP60E^KK105476^* | *Tsubouchi et al., Curr Biol 2012* |
| *NaCP60E^GD2219^* | *Tsubouchi et al., Curr Biol 2012* |
| *NaCP60E^GD9590^* | *Neely et al., Cell 2010* |
| *Gat^KK107895^* | *Frenkel et al., Cell Rep 2017* |
| *Wnt2^JF03377^* | *Trinh et al., Mol Metab 2019* |
| *Wnt4^JF03378^* | *Trinh et al., Mol Metab 2019* |
| *Wnt5^HM05020^* | *Yan et al., Dev Cell 2014* |
| *CG10348^JF02421^* | *Rogers et al., Dev Biol 2014* |
| *CG10348^HMS02808^* | *Tegeder et all., J Neurooncol 2019* |
| ***Gene name*** | ***Studies used the line*** |
| *Nipped-A^HMS00167^* | *Tauc et al., Development 2017; McCarthy et al., Molecular Biology of Cell 2018; Flegel et al. Genetics 2016* |
| *rhi^HMS00069^* | *Zhou et al., Sci Adv 2019* |
| *rhi^GL00041^* | *Theron et al., Nuc Ac Res, 2018; Kneuss et al., Gen and Dev, 2019* |
| *Chd3^GL00320^* | *Yan et al., Dev Cell 2014* |
| *Chd3^HMS00302^* | *Ellis et al., Plos One 2015* |
| *aurB^JF03107^* | *Wang et al., Curr Biol 2015* |
| *aurB^GL00202^* | *Wang et al., Curr Biol 2015; Radford et al., Plos Gen 2015* |
| *Caf1-105^0870-G4^* | *N/A* |
| *Caf1-105^HMC06575^* | *N/A* |
| *Taf12L^HMC05336^* | *Neves et al., Biology Open 2019* |
| *CG10445^GL01475^* | *Yan et al., PNAS 2015* |
| *Phf7^GL00455^* | *Yan et al., Dev Cell 2014* |
| *Kdm4B^GLV21041^* | *Zhou et al., Sci Adv 2019* |
| *Kdm4B^HMC04910^* | *Rotelli et al., G3 2019* |

***Q10.*** *“An interaction was considered to be validated when the observed trend was reproduced by multiple RNAi lines when available.” How validation was accomplished when multiple lines were unavailable is unclear.”*

**Authors’ response:** We thank the reviewer for this comment. We agree with the reviewer that we were not able to validate interactions in cases where only one fly line was available for interaction studies. We have modified the text to refer to results from these experiments as “*potential positive interaction*”, “*potential negative interaction*” and “*potential no interaction*”. These changes in nomenclature were clarified in **Methods** and adjusted in **S3 File**.

***Materials and Methods, Page 33****: ”Interactions assessed with only one fly line were considered as “potential negative”, “potential positive” or “potential no interaction” (S3 File).”*

***Q11.*** *“When epistatic effects were not confirmed with more than two RNAi lines, if available, the interaction was considered to be additive.” If epistatic effects cannot be confirmed with multiple lines, that should be noted and assigned a separate category”*

**Authors’ response:** We agree with this comment from the reviewer. Based on previous reviewer comments, we have changed our definition of genetic interactions to only include those where the observed values are different compared with the expected prediction, calculated using a multiplicative model (**see page 2 of this document**). In cases where more than one fly line was available to test the interaction, the discordant results were labeled as “*not validated*”. These changes in our labeling are updated in **S3 File**.

**REFERENCES**

- Bai, H., P. Kang, A. M. Hernandez and M. Tatar (2013). "Activin signaling targeted by insulin/dFOXO regulates aging and muscle proteostasis in Drosophila." PLoS Genet **9**(11): e1003941.
- Balmer, S., A. Dussert, G. M. Collu, E. Benitez, C. Iomini and M. Mlodzik (2015). "Components of Intraflagellar Transport Complex A Function Independently of the Cilium to Regulate Canonical Wnt Signaling in Drosophila." Dev Cell **34**(6): 705-718.
- Baryshnikova, A., M. Costanzo, Y. Kim, H. Ding, J. Koh, K. Toufighi, J. Y. Youn, J. Ou, B. J. San Luis, S. Bandyopadhyay, M. Hibbs, D. Hess, A. C. Gingras, G. D. Bader, O. G. Troyanskaya, G. W. Brown, B. Andrews, C. Boone and C. L. Myers (2010). "Quantitative analysis of fitness and genetic interactions in yeast on a genome scale." Nat Methods **7**(12): 1017-1024.
- Baskar, R., A. Bakrhat and U. Abdu (2019). "GFP-Forked, a genetic reporter for studying Drosophila oocyte polarity." Biol Open **8**(1).
- Bilen, J. and N. M. Bonini (2007). "Genome-wide screen for modifiers of ataxin-3 neurodegeneration in Drosophila." PLoS Genet **3**(10): 1950-1964.
- Branco, J., I. Al-Ramahi, L. Ukani, A. M. Perez, P. Fernandez-Funez, D. Rincon-Limas and J. Botas (2008). "Comparative analysis of genetic modifiers in Drosophila points to common and distinct mechanisms of pathogenesis among polyglutamine diseases." Hum Mol Genet **17**(3): 376-390.
- Buffolo, M., A. C. Batista Possidonio, C. Mermelstein and H. Araujo (2015). "A conserved role for calpains during myoblast fusion." Genesis **53**(7): 417-430.
- Chen, S., Y. E. Zhang and M. Long (2010). "New genes in Drosophila quickly become essential." Science **330**(6011): 1682-1685.
- Chubak, M. C., K. C. J. Nixon, M. H. Stone, N. Raun, S. L. Rice, M. Sarikahya, S. G. Jones, T. A. Lyons, T. E. Jakub, R. L. M. Mainland, M. J. Knip, T. N. Edwards and J. M. Kramer (2019). "Individual components of the SWI/SNF chromatin remodelling complex have distinct roles in memory neurons of the Drosophila mushroom body." Dis Model Mech **12**(3).
- Clark, A. G. and L. Wang (1997). "Epistasis in measured genotypes: Drosophila P-element insertions." Genetics **147**(1): 157-163.
- Clark, K. A. and J. L. Kadrmas (2013). "Drosophila melanogaster muscle LIM protein and alpha-actinin function together to stabilize muscle cytoarchitecture: a potential role for Mlp84B in actin-crosslinking." Cytoskeleton (Hoboken) **70**(6): 304-316.
- Copeland, J. M., J. Cho, T. Lo, Jr., J. H. Hur, S. Bahadorani, T. Arabyan, J. Rabie, J. Soh and D. W. Walker (2009). "Extension of Drosophila life span by RNAi of the mitochondrial respiratory chain." Curr Biol **19**(19): 1591-1598.
- Cukier, H. N., A. M. Perez, A. L. Collins, Z. Zhou, H. Y. Zoghbi and J. Botas (2008). "Genetic modifiers of MeCP2 function in Drosophila." PLoS Genet **4**(9): e1000179.
- Cziko, A. M., C. T. McCann, I. C. Howlett, S. A. Barbee, R. P. Duncan, R. Luedemann, D. Zarnescu, K. E. Zinsmaier, R. R. Parker and M. Ramaswami (2009). "Genetic modifiers of dFMR1 encode RNA granule components in Drosophila." Genetics **182**(4): 1051-1060.
- Dai, J., B. Estrada, S. Jacobs, B. J. Sanchez-Sanchez, J. Tang, M. Ma, P. Magadan-Corpas, J. C. Pastor-Pareja and M. D. Martin-Bermudo (2018). "Dissection of Nidogen function in Drosophila reveals tissue-specific mechanisms of basement membrane assembly." PLoS Genet **14**(9): e1007483.
- Dietz, K. N., L. Di Stefano, R. C. Maher, H. Zhu, M. E. Macdonald, J. F. Gusella and J. A. Walker (2015). "The Drosophila Huntington's disease gene ortholog dhtt influences chromatin regulation during development." Hum Mol Genet **24**(2): 330-345.
- Dixon, S. J., M. Costanzo, A. Baryshnikova, B. Andrews and C. Boone (2009). "Systematic mapping of genetic interaction networks." Annu Rev Genet **43**: 601-625.
- Duncan, A. M., T. Ozawa, H. Suzuki and R. Rozen (1993). "Assignment of the gene for the core protein II (UQCRC2) subunit of the mitochondrial cytochrome bc1 complex to human chromosome 16p12." Genomics **18**(2): 455-456.
- Elena, S. F. and R. E. Lenski (1997). "Test of synergistic interactions among deleterious mutations in bacteria." Nature **390**(6658): 395-398.
- Ellis, K., C. Friedman and B. Yedvobnick (2015). "Drosophila domino Exhibits Genetic Interactions with a Wide Spectrum of Chromatin Protein-Encoding Loci." PLoS One **10**(11): e0142635.
- Flegel, K., O. Grushko, K. Bolin, E. Griggs and L. Buttitta (2016). "Roles for the Histone Modifying and Exchange Complex NuA4 in Cell Cycle Progression in Drosophila melanogaster." Genetics **203**(3): 1265-1281.
- Frenkel, L., N. I. Muraro, A. N. Beltran Gonzalez, M. S. Marcora, G. Bernabo, C. Hermann-Luibl, J. I. Romero, C. Helfrich-Forster, E. M. Castano, C. Marino-Busjle, D. J. Calvo and M. F. Ceriani (2017). "Organization of Circadian Behavior Relies on Glycinergic Transmission." Cell Rep **19**(1): 72-85.
- Fulga, T. A., E. M. McNeill, R. Binari, J. Yelick, A. Blanche, M. Booker, B. R. Steinkraus, M. Schnall-Levin, Y. Zhao, T. DeLuca, F. Bejarano, Z. Han, E. C. Lai, D. P. Wall, N. Perrimon and D. Van Vactor (2015). "A transgenic resource for conditional competitive inhibition of conserved Drosophila microRNAs." Nat Commun **6**: 7279.
- Gamberi, C., D. R. Hipfner, M. Trudel and W. D. Lubell (2017). "Bicaudal C mutation causes myc and TOR pathway up-regulation and polycystic kidney disease-like phenotypes in Drosophila." PLoS Genet **13**(4): e1006694.
- Gerlach, S. U., T. Eichenlaub and H. Herranz (2018). "Yorkie and JNK Control Tumorigenesis in Drosophila Cells with Cytokinesis Failure." Cell Rep **23**(5): 1491-1503.
- Ghezzi, A., B. J. Liebeskind, A. Thompson, N. S. Atkinson and H. H. Zakon (2014). "Ancient association between cation leak channels and Mid1 proteins is conserved in fungi and animals." Front Mol Neurosci **7**: 15.
- Ghosh, R., S. Vegesna, R. Safi, H. Bao, B. Zhang, D. R. Marenda and F. L. Liebl (2014). "Kismet positively regulates glutamate receptor localization and synaptic transmission at the Drosophila neuromuscular junction." PLoS One **9**(11): e113494.
- Girirajan, S., J. A. Rosenfeld, B. P. Coe, S. Parikh, N. Friedman, A. Goldstein, R. A. Filipink, J. S. McConnell, B. Angle and W. S. Meschino (2012). "Phenotypic heterogeneity of genomic disorders and rare copy-number variants." New England Journal of Medicine **367**(14): 1321-1331.
- Gomez-Roman, N., C. Grandori, R. N. Eisenman and R. J. White (2003). "Direct activation of RNA polymerase III transcription by c-Myc." Nature **421**(6920): 290-294.
- Greene, C. S., A. Krishnan, A. K. Wong, E. Ricciotti, R. A. Zelaya, D. S. Himmelstein, R. Zhang, B. M. Hartmann, E. Zaslavsky, S. C. Sealfon, D. I. Chasman, G. A. FitzGerald, K. Dolinski, T. Grosser and O. G. Troyanskaya (2015). "Understanding multicellular function and disease with human tissue-specific networks." Nat Genet **47**(6): 569-576.
- Grice, S. J., J. L. Liu and C. Webber (2015). "Synergistic interactions between Drosophila orthologues of genes spanned by de novo human CNVs support multiple-hit models of autism." PLoS Genet **11**(3): e1004998.
- Grossman, T. R., A. Gamliel, R. J. Wessells, O. Taghli-Lamallem, K. Jepsen, K. Ocorr, J. R. Korenberg, K. L. Peterson, M. G. Rosenfeld, R. Bodmer and E. Bier (2011). "Over-expression of DSCAM and COL6A2 cooperatively generates congenital heart defects." PLoS Genet **7**(11): e1002344.
- Harris, K. P., Y. V. Zhang, Z. D. Piccioli, N. Perrimon and J. T. Littleton (2016). "The postsynaptic t-SNARE Syntaxin 4 controls traffic of Neuroligin 1 and Synaptotagmin 4 to regulate retrograde signaling." Elife **5**.
- Hassan, A., Y. Timerman, R. Hamdan, N. Sela, A. Avetisyan, N. Halachmi and A. Salzberg (2018). "An RNAi Screen Identifies New Genes Required for Normal Morphogenesis of Larval Chordotonal Organs." G3 (Bethesda) **8**(6): 1871-1884.
- Hevia, C. F., A. Lopez-Varea, N. Esteban and J. F. de Celis (2017). "A Search for Genes Mediating the Growth-Promoting Function of TGFbeta in the Drosophila melanogaster Wing Disc." Genetics **206**(1): 231-249.
- Horn, T., T. Sandmann, B. Fischer, E. Axelsson, W. Huber and M. Boutros (2011). "Mapping of signaling networks through synthetic genetic interaction analysis by RNAi." Nat Methods **8**(4): 341-346.
- Huang, N., I. Lee, E. M. Marcotte and M. E. Hurles (2010). "Characterising and predicting haploinsufficiency in the human genome." PLoS Genet **6**(10): e1001154.
- Iyer, J., M. D. Singh, M. Jensen, P. Patel, L. Pizzo, E. Huber, H. Koerselman, A. T. Weiner, P. Lepanto, K. Vadodaria, A. Kubina, Q. Wang, A. Talbert, S. Yennawar, J. Badano, J. R. Manak, M. M. Rolls, A. Krishnan and S. Girirajan (2018). "Pervasive genetic interactions modulate neurodevelopmental defects of the autism-associated 16p11.2 deletion in Drosophila melanogaster." Nat Commun **9**(1): 2548.
- Iyer, J., Q. Wang, T. Le, L. Pizzo, S. Gronke, S. S. Ambegaokar, Y. Imai, A. Srivastava, B. L. Troisi, G. Mardon, R. Artero, G. R. Jackson, A. M. Isaacs, L. Partridge, B. Lu, J. P. Kumar and S. Girirajan (2016). "Quantitative Assessment of Eye Phenotypes for Functional Genetic Studies Using Drosophila melanogaster." G3 (Bethesda) **6**(5): 1427-1437.
- Ji, X., R. L. Kember, C. D. Brown and M. Bucan (2016). "Increased burden of deleterious variants in essential genes in autism spectrum disorder." Proc Natl Acad Sci U S A **113**(52): 15054-15059.
- Jonikas, M. C., S. R. Collins, V. Denic, E. Oh, E. M. Quan, V. Schmid, J. Weibezahn, B. Schwappach, P. Walter, J. S. Weissman and M. Schuldiner (2009). "Comprehensive characterization of genes required for protein folding in the endoplasmic reticulum." Science **323**(5922): 1693-1697.
- Kamiyama, D., R. McGorty, R. Kamiyama, M. D. Kim, A. Chiba and B. Huang (2015). "Specification of Dendritogenesis Site in Drosophila aCC Motoneuron by Membrane Enrichment of Pak1 through Dscam1." Dev Cell **35**(1): 93-106.
- Kang, H., J. Zhao, X. Jiang, G. Li, W. Huang, H. Cheng and R. Duan (2019). "Drosophila Netrin-B controls mushroom body axon extension and regulates courtship-associated learning and memory of a Drosophila fragile X syndrome model." Mol Brain **12**(1): 52.
- Karim, F. D., H. C. Chang, M. Therrien, D. A. Wassarman, T. Laverty and G. M. Rubin (1996). "A screen for genes that function downstream of Ras1 during Drosophila eye development." Genetics **143**(1): 315-329.
- Katz, M. J., J. M. Acevedo, C. Loenarz, D. Galagovsky, P. Liu-Yi, M. Perez-Pepe, A. Thalhammer, R. Sekirnik, W. Ge, M. Melani, M. G. Thomas, S. Simonetta, G. L. Boccaccio, C. J. Schofield, M. E. Cockman, P. J. Ratcliffe and P. Wappner (2014). "Sudestada1, a Drosophila ribosomal prolyl-hydroxylase required for mRNA translation, cell homeostasis, and organ growth." Proc Natl Acad Sci U S A **111**(11): 4025-4030.
- Kawasaki, F., N. L. Koonce, L. Guo, S. Fatima, C. Qiu, M. T. Moon, Y. Zheng and R. W. Ordway (2016). "Small heat shock proteins mediate cell-autonomous and -nonautonomous protection in a Drosophila model for environmental-stress-induced degeneration." Dis Model Mech **9**(9): 953-964.
- Kim, W., H. D. Kim, Y. Jung, J. Kim and J. Chung (2015). "Drosophila Low Temperature Viability Protein 1 (LTV1) Is Required for Ribosome Biogenesis and Cell Growth Downstream of Drosophila Myc (dMyc)." J Biol Chem **290**(21): 13591-13604.
- Kneuss, E., M. Munafo, E. L. Eastwood, U. S. Deumer, J. B. Preall, G. J. Hannon and B. Czech (2019). "Specialization of the Drosophila nuclear export family protein Nxf3 for piRNA precursor export." Genes Dev **33**(17-18): 1208-1220.
- Krishnan, A., R. Zhang, V. Yao, C. L. Theesfeld, A. K. Wong, A. Tadych, N. Volfovsky, A. Packer, A. Lash and O. G. Troyanskaya (2016). "Genome-wide prediction and functional characterization of the genetic basis of autism spectrum disorder." Nat Neurosci **19**(11): 1454-1462.
- Kuntz, S., B. Poeck and R. Strauss (2017). "Visual Working Memory Requires Permissive and Instructive NO/cGMP Signaling at Presynapses in the Drosophila Central Brain." Curr Biol **27**(5): 613-623.
- Lee, S., W. D. Jones and D. H. Kim (2020). "A cyclic nucleotide-gated channel in the brain regulates olfactory modulation through neuropeptide F in fruit fly Drosophila melanogaster." Arch Insect Biochem Physiol **103**(1): e21620.
- Liu, Z. C., N. Odell and E. R. Geisbrecht (2013). "Drosophila importin-7 functions upstream of the Elmo signaling module to mediate the formation and stability of muscle attachments." J Cell Sci **126**(Pt 22): 5210-5223.
- Mackay, T. F. (2014). "Epistasis and quantitative traits: using model organisms to study gene-gene interactions." Nat Rev Genet **15**(1): 22-33.
- Mahmoudi, S., L. Xu and A. Brunet (2019). "Turning back time with emerging rejuvenation strategies." Nat Cell Biol **21**(1): 32-43.
- Mani, R., R. P. St Onge, J. L. t. Hartman, G. Giaever and F. P. Roth (2008). "Defining genetic interaction." Proc Natl Acad Sci U S A **105**(9): 3461-3466.
- McCammon, J. M., A. Blaker-Lee, X. Chen and H. Sive (2017). "The 16p11.2 homologs fam57ba and doc2a generate certain brain and body phenotypes." Hum Mol Genet **26**(19): 3699-3712.
- McCarthy, A., A. Deiulio, E. T. Martin, M. Upadhyay and P. Rangan (2018). "Tip60 complex promotes expression of a differentiation factor to regulate germline differentiation in female Drosophila." Mol Biol Cell **29**(24): 2933-2945.
- Mills, W. K., Y. C. G. Lee, A. M. Kochendoerfer, E. M. Dunleavy and G. H. Karpen (2019). "RNA from a simple-tandem repeat is required for sperm maturation and male fertility in Drosophila melanogaster." Elife **8**.
- Nash, H. A., R. L. Scott, B. C. Lear and R. Allada (2002). "An unusual cation channel mediates photic control of locomotion in Drosophila." Curr Biol **12**(24): 2152-2158.
- Neely, G. G., K. Kuba, A. Cammarato, K. Isobe, S. Amann, L. Zhang, M. Murata, L. Elmen, V. Gupta, S. Arora, R. Sarangi, D. Dan, S. Fujisawa, T. Usami, C. P. Xia, A. C. Keene, N. N. Alayari, H. Yamakawa, U. Elling, C. Berger, M. Novatchkova, R. Koglgruber, K. Fukuda, H. Nishina, M. Isobe, J. A. Pospisilik, Y. Imai, A. Pfeufer, A. A. Hicks, P. P. Pramstaller, S. Subramaniam, A. Kimura, K. Ocorr, R. Bodmer and J. M. Penninger (2010). "A global in vivo Drosophila RNAi screen identifies NOT3 as a conserved regulator of heart function." Cell **141**(1): 142-153.
- Neuert, H., Y. Yuva-Aydemir, M. Silies and C. Klambt (2017). "Different modes of APC/C activation control growth and neuron-glia interaction in the developing Drosophila eye." Development **144**(24): 4673-4683.
- Neves, A. and R. N. Eisenman (2019). "Distinct gene-selective roles for a network of core promoter factors in Drosophila neural stem cell identity." Biol Open **8**(4).
- O'Donovan, K. J., J. Diedler, G. C. Couture, J. J. Fak and R. B. Darnell (2010). "The onconeural antigen cdr2 is a novel APC/C target that acts in mitosis to regulate c-myc target genes in mammalian tumor cells." PLoS One **5**(4): e10045.
- Oortveld, M. A., S. Keerthikumar, M. Oti, B. Nijhof, A. C. Fernandes, K. Kochinke, A. Castells-Nobau, E. van Engelen, T. Ellenkamp, L. Eshuis, A. Galy, H. van Bokhoven, B. Habermann, H. G. Brunner, C. Zweier, P. Verstreken, M. A. Huynen and A. Schenck (2013). "Human intellectual disability genes form conserved functional modules in Drosophila." PLoS Genet **9**(10): e1003911.
- Orme, M. H., G. Liccardi, N. Moderau, R. Feltham, S. Wicky-John, T. Tenev, L. Aram, R. Wilson, K. Bianchi, O. Morris, C. Monteiro Domingues, D. Robertson, M. Tare, A. Wepf, D. Williams, A. Bergmann, M. Gstaiger, E. Arama, P. S. Ribeiro and P. Meier (2016). "The unconventional myosin CRINKLED and its mammalian orthologue MYO7A regulate caspases in their signalling roles." Nat Commun **7**: 10972.
- Penserga, T., S. R. Kudumala, R. Poulos and T. A. Godenschwege (2019). "A Role for Drosophila Amyloid Precursor Protein in Retrograde Trafficking of L1-Type Cell Adhesion Molecule Neuroglian." Front Cell Neurosci **13**: 322.
- Pizzo, L., M. Jensen, A. Polyak, J. A. Rosenfeld, K. Mannik, A. Krishnan, E. McCready, O. Pichon, C. Le Caignec, A. Van Dijck, K. Pope, E. Voorhoeve, J. Yoon, P. Stankiewicz, S. W. Cheung, D. Pazuchanics, E. Huber, V. Kumar, R. L. Kember, F. Mari, A. Curro, L. Castiglia, O. Galesi, E. Avola, T. Mattina, M. Fichera, L. Mandara, M. Vincent, M. Nizon, S. Mercier, C. Beneteau, S. Blesson, D. Martin-Coignard, A. L. Mosca-Boidron, J. H. Caberg, M. Bucan, S. Zeesman, M. J. M. Nowaczyk, M. Lefebvre, L. Faivre, P. Callier, C. Skinner, B. Keren, C. Perrine, P. Prontera, N. Marle, A. Renieri, A. Reymond, R. F. Kooy, B. Isidor, C. Schwartz, C. Romano, E. Sistermans, D. J. Amor, J. Andrieux and S. Girirajan (2018). "Rare variants in the genetic background modulate cognitive and developmental phenotypes in individuals carrying disease-associated variants." Genet Med.
- Pletcher, R. C., S. L. Hardman, S. F. Intagliata, R. L. Lawson, A. Page and J. M. Tennessen (2019). "A Genetic Screen Using the Drosophila melanogaster TRiP RNAi Collection To Identify Metabolic Enzymes Required for Eye Development." G3 (Bethesda) **9**(7): 2061-2070.
- Qiu, Y., T. Arbogast, S. M. Lorenzo, H. Li, S. C. Tang, E. Richardson, O. Hong, S. Cho, O. Shanta, T. Pang, C. Corsello, C. K. Deutsch, C. Chevalier, E. E. Davis, L. M. Iakoucheva, Y. Herault, N. Katsanis, K. Messer and J. Sebat (2019). "Oligogenic Effects of 16p11.2 Copy-Number Variation on Craniofacial Development." Cell Rep **28**(13): 3320-3328 e3324.
- Radford, S. J., T. L. Hoang, A. A. Gluszek, H. Ohkura and K. S. McKim (2015). "Lateral and End-On Kinetochore Attachments Are Coordinated to Achieve Bi-orientation in Drosophila Oocytes." PLoS Genet **11**(10): e1005605.
- Raza, Q., J. Y. Choi, Y. Li, R. M. O'Dowd, S. C. Watkins, M. Chikina, Y. Hong, N. L. Clark and A. V. Kwiatkowski (2019). "Evolutionary rate covariation analysis of E-cadherin identifies Raskol as a regulator of cell adhesion and actin dynamics in Drosophila." PLoS Genet **15**(2): e1007720.
- Reiter, L. T., L. Potocki, S. Chien, M. Gribskov and E. Bier (2001). "A systematic analysis of human disease-associated gene sequences in Drosophila melanogaster." Genome Res **11**(6): 1114-1125.
- Rogers, W. A., S. Grover, S. J. Stringer, J. Parks, M. Rebeiz and T. M. Williams (2014). "A survey of the trans-regulatory landscape for Drosophila melanogaster abdominal pigmentation." Dev Biol **385**(2): 417-432.
- Rotelli, M. D., A. M. Bolling, A. W. Killion, A. J. Weinberg, M. J. Dixon and B. R. Calvi (2019). "An RNAi Screen for Genes Required for Growth of Drosophila Wing Tissue." G3 (Bethesda) **9**(10): 3087-3100.
- Scholz, N., N. Ehmann, D. Sachidanandan, C. Imig, B. H. Cooper, O. Jahn, K. Reim, N. Brose, J. Meyer, M. Lamberty, S. Altrichter, A. Bormann, S. Hallermann, M. Pauli, M. Heckmann, C. Stigloher, T. Langenhan and R. J. Kittel (2019). "Complexin cooperates with Bruchpilot to tether synaptic vesicles to the active zone cytomatrix." J Cell Biol **218**(3): 1011-1026.
- Schwartz, S., M. Truglio, M. J. Scott and H. L. Fitzsimons (2016). "Long-Term Memory in Drosophila Is Influenced by Histone Deacetylase HDAC4 Interacting with SUMO-Conjugating Enzyme Ubc9." Genetics **203**(3): 1249-1264.
- Singh, M. D., M. Jensen, M. Lasser, E. Huber, T. Yusuff, L. Pizzo, B. Lifschutz, I. Desai, A. Kubina, S. Yennawar, S. Kim, J. Iyer, D. E. Rincon-Limas, L. A. Lowery and S. Girirajan (2020). "NCBP2 modulates neurodevelopmental defects of the 3q29 deletion in Drosophila and Xenopus laevis models." PLoS Genet **16**(2): e1008590.
- Stetina, T., V. Kostal and J. Korbelova (2015). "The Role of Inducible Hsp70, and Other Heat Shock Proteins, in Adaptive Complex of Cold Tolerance of the Fruit Fly (Drosophila melanogaster)." PLoS One **10**(6): e0128976.
- Straub, J., A. Gregor, T. Sauerer, A. Fliedner, L. Distel, C. Suchy, A. B. Ekici, F. Ferrazzi and C. Zweier (2020). "Genetic interaction screen for severe neurodevelopmental disorders reveals a functional link between Ube3a and Mef2 in Drosophila melanogaster." Sci Rep **10**(1): 1204.
- Tegeder, I., K. Thiel, S. Erkek, P. D. Johann, J. Berlandi, V. Thatikonda, M. C. Fruhwald, M. Kool, A. Jeibmann and M. Hasselblatt (2019). "Functional relevance of genes predicted to be affected by epigenetic alterations in atypical teratoid/rhabdoid tumors." J Neurooncol **141**(1): 43-55.
- Thaker, H. M. and D. R. Kankel (1992). "Mosaic analysis gives an estimate of the extent of genomic involvement in the development of the visual system in Drosophila melanogaster." Genetics **131**(4): 883-894.
- Theron, E., S. Maupetit-Mehouas, P. Pouchin, L. Baudet, E. Brasset and C. Vaury (2018). "The interplay between the Argonaute proteins Piwi and Aub within Drosophila germarium is critical for oogenesis, piRNA biogenesis and TE silencing." Nucleic Acids Res **46**(19): 10052-10065.
- Thevenon, D., I. Seffouh, C. Pillet, X. Crespo-Yanez, M. O. Fauvarque and E. Taillebourg (2020). "A Nucleolar Isoform of the Drosophila Ubiquitin Specific Protease dUSP36 Regulates MYC-Dependent Cell Growth." Front Cell Dev Biol **8**: 506.
- Trinh, I., O. B. Gluscencova and G. L. Boulianne (2019). "An in vivo screen for neuronal genes involved in obesity identifies Diacylglycerol kinase as a regulator of insulin secretion." Mol Metab **19**: 13-23.
- Tsubouchi, A., J. C. Caldwell and W. D. Tracey (2012). "Dendritic filopodia, Ripped Pocket, NOMPC, and NMDARs contribute to the sense of touch in Drosophila larvae." Curr Biol **22**(22): 2124-2134.
- Veltman, J. A. and H. G. Brunner (2010). "Understanding variable expressivity in microdeletion syndromes." Nat Genet **42**(3): 192-193.
- Wang, H. D., P. Kazemi-Esfarjani and S. Benzer (2004). "Multiple-stress analysis for isolation of Drosophila longevity genes." Proc Natl Acad Sci U S A **101**(34): 12610-12615.
- Wang, Y., M. Antunes, A. E. Anderson, J. L. Kadrmas, A. Jacinto and M. J. Galko (2015). "Integrin Adhesions Suppress Syncytium Formation in the Drosophila Larval Epidermis." Curr Biol **25**(17): 2215-2227.
- Watanabe, L. P., C. Gordon, M. Y. Momeni and N. C. Riddle (2020). "Genetic Networks Underlying Natural Variation in Basal and Induced Activity Levels in Drosophila melanogaster." G3 (Bethesda) **10**(4): 1247-1260.
- Xiao, X., C. Chen, T. M. Yu, J. Ou, M. Rui, Y. Zhai, Y. He, L. Xue and M. S. Ho (2017). "Molecular Chaperone Calnexin Regulates the Function of Drosophila Sodium Channel Paralytic." Front Mol Neurosci **10**: 57.
- Yagi, Y., Y. M. Lim, L. Tsuda and Y. Nishida (2013). "fat facets induces polyubiquitination of Imd and inhibits the innate immune response in Drosophila." Genes Cells **18**(11): 934-945.
- Yan, D., R. A. Neumuller, M. Buckner, K. Ayers, H. Li, Y. Hu, D. Yang-Zhou, L. Pan, X. Wang, C. Kelley, A. Vinayagam, R. Binari, S. Randklev, L. A. Perkins, T. Xie, L. Cooley and N. Perrimon (2014). "A regulatory network of Drosophila germline stem cell self-renewal." Dev Cell **28**(4): 459-473.
- Yu, Y., R. Huang, J. Ye, V. Zhang, C. Wu, G. Cheng, J. Jia and L. Wang (2016). "Regulation of starvation-induced hyperactivity by insulin and glucagon signaling in adult Drosophila." Elife **5**.
- Zang, Y., M. Wan, M. Liu, H. Ke, S. Ma, L. P. Liu, J. Q. Ni and J. C. Pastor-Pareja (2015). "Plasma membrane overgrowth causes fibrotic collagen accumulation and immune activation in Drosophila adipocytes." Elife **4**: e07187.
- Zhou, J., L. Xu, X. Duan, W. Liu, X. Zhao, X. Wang, W. Shang, X. Fang, H. Yang, L. Jia, J. Bai, J. Zhao, L. Wang and C. Tong (2019). "Large-scale RNAi screen identified Dhpr as a regulator of mitochondrial morphology and tissue homeostasis." Sci Adv **5**(9): eaax0365.
- Zhu, J. Y., Y. Fu, M. Nettleton, A. Richman and Z. Han (2017). "High throughput in vivo functional validation of candidate congenital heart disease genes in Drosophila." Elife **6**.
