# Supplementary material for: Functional assessment of the “two-hit” model for neurodevelopmental defects in Drosophila and X. laevis
Source: PLoS Genet. 2021 Apr 5;17(4):e1009112. doi: 10.1371/journal.pgen.1009112 (PMC8049494; doi:10.1371/journal.pgen.1009112)
Supplement: Attachment — Submitted filename: Rebuttal_FINAL2.docx [file pgen.1009112.s038.docx]

**RESPONSE TO EDITORS’ AND REVIEWERS’ COMMENTS**

We thank the reviewers for their comments on our manuscript. Please find below **a summary of new analyses and revisions**, followed by a **point-by-point response to Reviewer’s 1 comments**.

(1) **Improved clarity of text**: We have modified the text and removed redundant sentences to improve the clarity of the presentation.
(2) **Multiplicative model for testing interactions**: We clarify that we only used a multiplicative model to identify genetic interactions as this model has been shown to be robust for quantitatively assessing functional interactions (*Mani et al, PNAS, 2008; Elena and Lenski, 1997; Gao et al, Genetics, 2010*). We modified the text to clarify this point.

**(3) Interactions of 16p12.1 homologs with second-hits:** Using interaction scores, we find that the interactions of 16p12.1 homologs with “second-hits” *are stronger* than interactions of 16p12.1 homologs with each other. We modified the **Abstract** and the **Discussion**, and included a **Supplemental S21** Figure.

**(4) We tested genetic interactions after accounting for gene counts and “controls”:** We compared our tested interactions with permuted gene sets to account for gene counts and appropriate “controls” in a human brain-specific interaction network.

- We find that 16p12.1 genes are weakly connected to each other compared to the connectivity of permuted sets of 16p11.2 genes or consecutive genes from the genome. We have modified the **Discussion** and included a **Supplementary S23 Figure** to address this comment.
- We found that the connectivity of 16p12.1 genes with the neurodevelopmental and functionally-related genes tested in our study was stronger than the matched gene sets (random genes, preferentially brain-expressed, and genes with no preferential brain expression).
- We clarify that we only intended to identify specific interacting pairs and not enrichment within gene-sets. We show that modulation of neurodevelopmental phenotypes by “second-hits” may occur through genetic interactions (such as interactions with *SETD5*), and that not all effects are additive.

**(5) Ameliorating interactions of 16p12.1 homologs with second-hits**: We clarify the comment regarding alleviating interactions of 16p12.1 homologs with second-hit genes.

**(6) Statistical analyses:** All new analyses have been updated in **S6 File**.

**(7) Data availability**: We note that RNA-sequencing datasets are uploaded in public repositories, including GEO (acc#GSE151330), SRA (acc#PRJNA635495), and Github (https://github.com/girirajanlab/16p12_fly_project), summary statistics for all experiments has been provided in **S6 File**, and all experimental data are provided as individual values in main and supplemental figures. Observed and expected values for the genetic interaction analyses are provided in **S4 File**.

**POINT-BY-POINT RESPONSE TO REVIEWER COMMENTS**

**REVIEWER 1**

***Q1****. “Thank you for the detailed responses to reviewer concerns. I think the paper is improved, but the root of most of the concerns has not been addressed. It is also still extremely dense and difficult to parse, and the study design limitations continue to impact the overall conclusions and interpretation.”*

**Authors’ response:** We have modified the text and removed redundant sentences to improve the clarity. We have also revised the manuscript to address the reviewer’s specific comments regarding study design limitations .

***Q2****. “It’s very helpful to specifically test the multiplicative model, but still unclear in places the meaning of additive, multiplicative, and interaction. If you are testing both additive and multiplicative models for each pair and calling it interaction if both are rejected, that could be clarified. In your example of 120% and 140% - additive is 160% and multiplicative is 168%, so are you calling interactions those that are significantly below 160% or above 168%? In the response you indicate that multiplicative is the best model of ‘interaction’ for functional effects, but in your example, you consider interaction to be deviation from the multiplicative model, so how you are using these could be further clarified.”*

**Authors’ response:** We thank the reviewer for appreciating the utility of the multiplicative model in our analyses. We would like to clarify that we only used the multiplicative model and not an “additive” model, as it has been shown to have better performance than other methods to identify functional interactions (*Mani et al*., *PNAS*, 2008, *Elena et al., Nature 1997; Gao et al, Genetics, 2010*). We also demonstrate in **Supplementary Figure S9** that both the expected and observed values fit within the scales of our phenotypic measures. The model tests whether the observed effects are different from the *expected* combined independent effects of knockdown of individual genes. The expected outcome is calculated as a product of the normalized effects observed with individual gene knockdowns. The expectation of the multiplicative model is that there will be *no* interaction between pairs of genes. Any deviation from the expected result is considered a genetic interaction. This can be either *positive*, where the observed phenotype is significantly alleviated than expected, or *negative*, where the observed phenotype is significantly worse than expected.

We included the term “additive” in the manuscript, as it is widely used in the genetics literature to refer to the *combined independent effects of individual genetic variants* (*Veltman and Brunner, Nature Genet., 2010*). However, we understand that using the term “additive” may be confusing, since we used a multiplicative model for the analysis. Therefore, we have replaced the word “*additive*” with “*combined independent effect of the individual genes*” at key areas in the main text.

*For example*, **Results, page 17**: “*was more severe compared to the phenotype observed for the knockdown of an individual 16p12.1 homolog, but significantly less severe than the expected effects, suggesting potential buffering against deleterious combined independent effects (62, 63) (Fig 4C, Fig 5D, S3 File).*”

***Q3****. “The big-picture interpretation of your data as 16p12.1 genes being less likely to interact with each other and more likely to interact with second-hits is still difficult to support with the comparisons you have made.”*

**Authors’ response:** In order to address this comment, we formally compared the *magnitude of genetic interactions* between 16p12.1 homologs and second-hits with those observed between 16p12.1 homologs. The interaction scores were significantly higher for interactions between 16p12.1 homologs and second-hits compared to interactions of 16p12.1 homologs with each other (Mann-Whitney two-tailed, p=0.0032, **Figure R1/ S21 Figure**). We also note that our analysis is based on ascertainment of rare functionally intolerant second-hit genes with conserved *Drosophila* homologs from severely affected children. We have modified the **Abstract**, **Introduction**, and **Discussion** to clarify this limitation in our interpretation. We have also added a comparison of interaction scores in **Supplemental S21 Figure**.

**Abstract, Page 2:** “*..interaction network, suggesting that interactions with “second-hit” genes may confer higher impact towards neurodevelopmental phenotypes.”*

**Discussion, Page 21.** “*Based on these observations, we propose that while genes carrying “second-hit” variants may additively contribute towards more severe phenotypes, they may also interact towards developmental phenotypes, conferring high impact towards variable defects associated with the 16p12.1 deletion (S21 Fig). The ultimate nature of the interactions will depend on the role of the individual CNV genes towards specific phenotypes, the identity of genes carrying “second-hits”, as well as the molecular complexity associated with each phenotypic domain (50).*”

**Figure R1.** **Interactions between 16p12.1 homologs and “second-hits” are of higher magnitude than those between 16p12.1 homologs.** (**A**) Absolute values of scores of interactions identified between 16p12.1 homologs are lower compared to those identified between the 16p12.1 homologs and “second-hit” homologs (n=3 for 16p12.1, n=37 for interactions with “second-hits”, two-tailed Mann-Whitney, *p=0.0032). (**B**) Comparison of absolute values of interaction scores between 16p12.1 homologs to those observed between the homologs and homologs of “second-hits” split by proband. Only one RNAi line per interacting gene is shown.

*Q4. “For example, the 16p11.2 region has more genes tested for interactions, so you might randomly sample 4 at a time and then compare 16p12.1 with the median of that set.”*

**Authors’ response:** To address the reviewer’s comment, we performed connectivity analyses in a human brain-specific interaction network and compared the median connectivity of the six human 16p12.1 genes present in the network to a distribution of median connectivity of 1000 permutations of six 16p11.2 genes (out of 25 total 16p11.2 genes in the network) or six random genes in the network (out of 14,759 total network genes). We observed that the median connectivity of 16p12.1 genes was located at the 0.4^th^ percentile compared to a distribution of 1000 simulations of six 16p11.2 genes, although this difference did not reach statistical significance (p=0.117, one-tailed z-score test, **Figure R2/S23A Figure**). In other words, 99.6% of simulated sets of six 16p11.2 genes had a higher connectivity than the six 16p12.1 genes in a human brain interaction network. Similarly, the connectivity of 16p12.1 genes was at the 9.2 percentile compared to 1000 sets of six random genes drawn from the genome (p=0.143, one-tailed z-score test). We have modified the **Discussion** section to include these observations and **S23 Figure**:

**Discussion, page 22**: “*Furthermore, the connectivity of 16p12.1 genes was lower than 99.6% of connectivity values of simulated sets of six 16p11.2 genes (0.4th percentile). In fact, the connectivity values of genes within 16p12.1 and 16p11.2 were in the lower (~6th) and higher (~79th) percentiles of connectivity values from simulated sets of contiguous genes in the genome, respectively (S23 Fig).*”

**Figure R2.** Cumulative frequency plot shows low connectivity of 16p12.1 genes (observed value shown as red dotted line) in a human brain-specific interaction network when compared with 1000 permutations of six 16p11.2 genes (blue, 0.4^th^ percentile, p=0.117, one-tailed z-score test) and random set of genes in the genome (green, 9.2^nd^ percentile, p=0.143, one-tailed z-score test). Cumulative frequency plot of 1000 simulations of bins of 25 contiguous genes (green)

*Q5. “Even more problematic is the ‘second-hit’ hypothesis expanded to candidate gene sets and no control sets. If you have selected genes by those most likely to interact because of their developmental role, etc., you need a matched comparison set to really test a specific hypothesis, e.g. are developmental genes more likely to interact than matched brain-expressed genes? are the second-hits observed in families more likely to interact than other genes that would be classified as ‘pathogenic’ in clinical testing for developmental disorders? What is the comparison for your selected gene lists?”*

**Authors’ response:** We apologize to the reviewer for the confusion, and clarify with the following points:

(1) We did not seek to identify enrichments but only intended to identify neurodevelopmental and functionally-related genes that may interact with 16p12.1 homologs (detailed in **S3 Table**) towards modulating neurodevelopmental phenotypes. We used two or more lines for testing 133 out of the 224 pairwise knockdowns tested, and we strongly believe that phenotypic effects validated using multiple fly lines have biological validity. We have modified the text to clarify this point.

**Results, Page 15**: “*Next, to identify interactors of 16p12.1 homologs towards developmental functions and pathways (****S10 Fig, S3 Table)****, we screened for interactions of the homologs with 25 dysregulated fly genes...*”

**Results, Page 16**: “*Overall, we identified 64 pairwise interactions between the 16p12.1 homologs and genes from established neuronal functions and transcriptome targets (Fig 4C-D, S2 Table), suggesting that phenotypes of 16p12.1 homologs can be modulated by genes within multiple neurodevelopmental pathways through genetic interactions.*”

(2) Although we did not perform the suggested control experiments with flies, we did assess whether 16p12.1 genes are more likely to interact with developmental genes or genes carrying second-hits compared to random sets of “control” genes in a human brain-specific interaction network. We compared the connectivity between four tested 16p12.1 genes and eight homologs of neurodevelopmental and functionally-related genes or 21 patient-specific second-hit genes tested in our *Drosophila* functional experiments with the connectivity between the four 16p12.1 genes and an equal number of genes from appropriate control lists as detailed below:

(a) We analyzed the median connectivity between the four tested 16p12.1 genes and 1000 simulated sets of eight genes that were (i) preferentially expressed in the human brain (n=3,865 genes); (ii) preferentially expressed in non-brain tissues (n=9,155 genes); and (iii) random network genes (n=14,759 genes), to compare with the observed connectivity of 16p12.1 genes with the eight neurodevelopmental and functionally-related genes tested in our study (**Figure R3A**).

*Note: Preferential gene expression was determined from GTEx Consortium data (GTEx Consortium, Science, 2015). Genes whose expression in a particular tissue was greater than the median+2*SD of its expression across all tissues were considered to be preferentially expressed in that tissue.*

We observed that 16p12.1 genes were significantly strongly connected to our tested neurodevelopmental and functionally-related genes compared to all “control” sets, i.e., random sets of brain-expressed genes (p=0.00159, one-tailed z-score test), non-brain-expressed genes (p=0.0145), and random genes in the network (p=0.0235).

(b) We calculated the connectivity between 16p12.1 genes and permutations of 21 genes from the Developmental Brain Disorder Gene (DBD) database, which curates a gene list for neurodevelopmental disorders including intellectual disability/developmental delay, autism, schizophrenia, and epilepsy (Gonzalez-Mantilla *et al*, *JAMA Psych*, 2016). We compared the median connectivity between 16p12.1 genes and the 21 tested second-hit genes with the connectivity of 16p12.1 genes with 1000 random draws of 21 genes from the DBD database, or random picks of 21 genes from the brain network (**Figure R3B**). *Note: DBD curates a gene list for neurodevelopmental disorders including intellectual disability/developmental delay, autism, schizophrenia, and epilepsy (Gonzalez-Mantilla et al, JAMA Psych, 2016) (out of n=419 total DBD genes).*

We observed that 16p12.1 genes were weakly connected to patient second-hit genes compared to simulated sets of DBD genes (7.4th percentile), although this did not reach statistical significance (p=0.0856, one-tailed z-score test). Also, 16p12.1 genes were weakly connected to patient second-hit genes than random gene sets (22.4th percentile, p=0.2137). These analyses show that 16p12.1 genes are more connected with the known neurodevelopmental and functionally-related genes compared to “control’ sets of preferentially brain expressed or non-brain expressed genes. Also, we did not observe strong connectivity with patient second-hits, suggesting that several of the second hits may not be functionally related to 16p12.1 genes and their combinatorial effects may be mostly “additive”.

**Figure R3.** Cumulative frequency plot of 1000 permutations of connectivity values in a human brain-specific interaction network between 16p12.1 genes with (**A**) eight random preferential brain expressed genes (blue), preferential non-brain expressed genes (orange), and random set of genes in the genome (green); or (**B**) 21 random candidate disease-associated genes from DBD (blue) or random genes (green). The median connectivity of 16p12.1 genes with neurodevelopmental and functionally-related genes or second-hit genes is represented by the red dotted lines.

*Q6. “How do you explain the attenuation effect if your hypothesis is that the 16p12.1 genes sensitize the genome to developmental effects? The majority of your selected genes seem to enhance, but patient 2nd hits seem to suppress- how do you interpret that with respect to your hypotheses?”*

**Authors’ response:** We thank the reviewer for this comment. We would like to clarify a few points regarding the analyses and interpretations in the manuscript:

(1) We first assessed whether a simultaneous knockdown of 16p12.1 genes with a patient-specific “second-hit” gene would lead to any phenotypic change compared to knockdown of individual 16p12.1 homologs. As detailed in **S2 Table**, we observed that 26/96 (27%) pairs tested led to an *enhanced* phenotype compared to knockdown of individual 16p12.1 homolog, while 6/96 (6%) led to a *suppression*. In contrast, we observed enhancements for 6/55 (11%) and 19/61 (31%) tested combinations with neurodevelopmental/functionally-related genes and transcriptome targets, respectively. We identified 12/55 (22%) suppressions for neurodevelopmental/functionally-related genes and 10/61 (16%) suppressions for transcriptome targets. Therefore, in line with our hypothesis that “second-hits” contribute to increased severity, we observed that a majority of phenotypic changes corresponded with enhancements for this gene category. The phenotypic changes for each pair are detailed in **S3 File**.
(2) We performed a second and independent analysis to quantitatively assess whether phenotypes altered with pairwise knockdowns could be attributed to genetic interactions. Using the multiplicative model, we identified pairs of genes whose knockdown showed a milder phenotype than expected (*positive interactions*) or worse than expected (*negative interactions*). *Please note that these results are based on comparisons with the expected outcome according to the multiplicative model, and therefore comprise a separate analysis from the identification of enhancers and suppressors of 16p12.1 homolog.* Therefore, a *positive or alleviating interaction* does not necessarily correspond with a *suppression* of the phenotype of the 16p12.1 homolog (*as described in (1)*). *For example*, we observed a *positive interaction* for knockdown of *Cen* with *shot* i.e., double knockdown leading to milder eye compared to the *expected*. However, the double knockdown of both *Cen* and *shot* still showed a more severe phenotype than with knockdown of just *Cen*, representing an *enhancement*. Similarly, *Rpn2* and *Dhc98D* enhanced the phenotype observed for *Sin* and *UQCRC-C2*, respectively, although these pairs were deemed as *positive or alleviating* interactions in a multiplicative model.

**Figure R4.** Positive or alleviating interactions, where the observed effect is less than expected, may also lead to enhancement of the phenotype observed for individual 16p12.1 homologs. Three examples are shown of positive interactions where the observed phenotype (in green) is milder than expected (in blue), but pairwise knockdown shows more severe phenotype compared to knockdown of individual homologs (represented as *Cen/C*, *Sin/C* or *UQCR-C2/C*).

(3) As stated by the reviewer, we observed a high number (31/96, 32%) of positive or alleviating interactions for patient-specific “second-hit” genes. While this gene category showed the highest percentage of positive interactions, this value was comparable to that observed for other gene sets (12/55 or 22% for neurodevelopmental/functionally related genes, and 17/61 or 28% for transcriptome targets). We don’t know exactly what this could be due to, but propose that the reason behind this higher number of positive interactions with patient-specific “second-hit” genes could be due to a potential buffering effect, which has been previously suggested to mitigate the combinatorial effect of disease-associated variants that otherwise would be too deleterious for the organism (*Hartman et al., Science 2001; Queitsch et al., PLoS Genet, 2012*). We have modified the **Results** section to clarify the two distinct analyses performed, and noted our explanation of potential “buffering” effects of second-hits:

**Results, Page 12**: “*We performed two independent analyses to interpret the combinatorial effect of 16p12.1 homologs on eye phenotypes. First, we assessed whether the phenotypic severity observed with knockdown of a 16p12.1 homolog is enhanced or suppressed with simultaneous knockdown of a second homolog…. Second, to quantitatively assess whether the change in phenotypic severity due to pairwise knockdowns could be attributed to genetic interactions, we applied a “multiplicative” model to the Flynotyper scores.*”

**Results, Page 17**: “*Interestingly, for 11/96 combinations tested with the multiplicative model, we found that the phenotype of the pairwise knockdown was more severe compared to the phenotype observed for the knockdown of an individual 16p12.1 homolog, but significantly less severe than the expected effects, suggesting potential buffering against deleterious combined independent gene effects (62, 63) (****Fig 4C, Fig 5D, S3 File****)*.”

*Q7. “I still have trouble sorting out well-rationalized a priori hypotheses from post hoc interpretation of observations. I appreciate the intention of the table delineating hypotheses, but these are still pretty vague to me. If your hypothesis is “16p12.1 homologs interact towards neurodevelopmental phenotypes,” does 3/12 support this? Is that more than non-CNV sets of 4 consecutive genes? You identified at least one interaction, but a smaller proportion were interacting than for 16p11.2, so a more specific hypothesis and comparison to a ‘control’ or ‘alternative’ condition would be helpful in clarifying the hypothesis.”*

**Authors’ response:** To address the reviewer comment, we sampled 1000 windows of 6 and 25 contiguous genes in the genome, after excluding regions overlapping 39 neurodevelopmental-associated CNVs (*Girirajan et al., NEJM 2012*), and calculated the network connectivity among the genes within each window. Then, we compared the median connectivity value of all 16p12.1 and 16p11.2 genes in the network to the distribution of connectivity values of the 1000 random sets of contiguous genes. We found that the connectivity of 16p12.1 genes was on the lower end of the distribution of connectivity of randomly drawn six contiguous genes (5.7^th^ percentile; p=0.130, one-tailed z-score test, **Figure R5/S23B-C Figure**). In contrast, the 16p11.2 genes was on the higher end of the 25-gene windows (78.6^th^ percentile) (p=0.246, one-tailed z-score test, **Figure R5/ S23B-C Figure**). We have modified the **Discussion** section to include these observations and **S23 Figure**:

**Discussion, page 22**: “*Furthermore, the connectivity of 16p12.1 genes was lower than 99.6% of connectivity values of simulated sets of six 16p11.2 genes (0.4th percentile). In fact, the connectivity values of genes within 16p12.1 and 16p11.2 were in the lower (~6th) and higher (~79th) percentiles of connectivity values from simulated sets of contiguous genes in the genome, respectively (S23 Fig).*”

**Figure R5**. *Cumulative frequency plot of 1000 simulations of bins of 25 contiguous genes (green) (****B****) or six contiguous genes (blue) (****C****), compared with the median connectivity values for 16p11.2 genes (green dotted line, 78.6^th^ percentile, one-tailed z-score test, p=0.246) and 16p12.1 genes (blue dotted line, 5.7^th^ percentile, p=0.130).*

**References**

- Consortium, G. T. (2015). "Human genomics. The Genotype-Tissue Expression (GTEx) pilot analysis: multitissue gene regulation in humans." Science 348(6235): 648-660.
- Elena, S. F. and R. E. Lenski (1997). "Test of synergistic interactions among deleterious mutations in bacteria." Nature 390(6658): 395-398.
- Gao, H., J. M. Granka and M. W. Feldman (2010). "On the classification of epistatic interactions." Genetics 184(3): 827-837.
- Girirajan, S., J. A. Rosenfeld, B. P. Coe, S. Parikh, N. Friedman, A. Goldstein, R. A. Filipink, J. S. McConnell, B. Angle and W. S. Meschino (2012). "Phenotypic heterogeneity of genomic disorders and rare copy-number variants." New England Journal of Medicine 367(14): 1321-1331.
- Gonzalez-Mantilla, A. J., A. Moreno-De-Luca, D. H. Ledbetter and C. L. Martin (2016). "A Cross-Disorder Method to Identify Novel Candidate Genes for Developmental Brain Disorders." JAMA Psychiatry 73(3): 275-283.
- Hartman, J. L. t., B. Garvik and L. Hartwell (2001). "Principles for the buffering of genetic variation." Science 291(5506): 1001-1004.
- Mani, R., R. P. St Onge, J. L. t. Hartman, G. Giaever and F. P. Roth (2008). "Defining genetic interaction." Proc Natl Acad Sci U S A 105(9): 3461-3466.
- Queitsch, C., K. D. Carlson and S. Girirajan (2012). "Lessons from model organisms: phenotypic robustness and missing heritability in complex disease." PLoS Genet 8(11): e1003041.
- Veltman, J. A. and H. G. Brunner (2010). "Understanding variable expressivity in microdeletion syndromes." Nat Genet 42(3): 192-193.
